# Correction: Ivanova, B. Stochastic Dynamic Mass Spectrometric Quantitative and Structural Analyses of Pharmaceutics and Biocides in Biota and Sewage Sludge. *Int. J. Mol. Sci.* 2023, *24*, 6306

**DOI:** 10.3390/ijms26083797

**Published:** 2025-04-17

**Authors:** Bojidarka Ivanova

**Affiliations:** Independent Researcher, 44139 Dortmund, Germany; bojidarka.ivanova@yahoo.com

Following a request from Dortmund University, the previous affiliation of the Guest Editor, Bojidarka Ivanova, in the original publication [1] has been updated to “Independent Researcher, 44139 Dortmund, Germany”. The email of author should be “bojidarka.ivanova@yahoo.com”. The author states that the scientific conclusions are unaffected. This correction was approved by the Academic Editor. The original publication has also been updated.
