# Supplementary material for: Stochastic Dynamic Mass Spectrometric Quantitative and Structural Analyses of Pharmaceutics and Biocides in Biota and Sewage Sludge
Source: Int J Mol Sci. 2023 Mar 27;24(7):6306. doi: 10.3390/ijms24076306 (PMC10094044; doi:10.3390/ijms24076306)
Supplement: Supplementary file 1 [file ijms-24-06306-s001.zip › ijms-2292505-supplementary.pdf]

Supplementary Materials

Stochastic dynamic quantitative and structural analyses of common used pharmaceutics and biocides in sewage sludge and biota

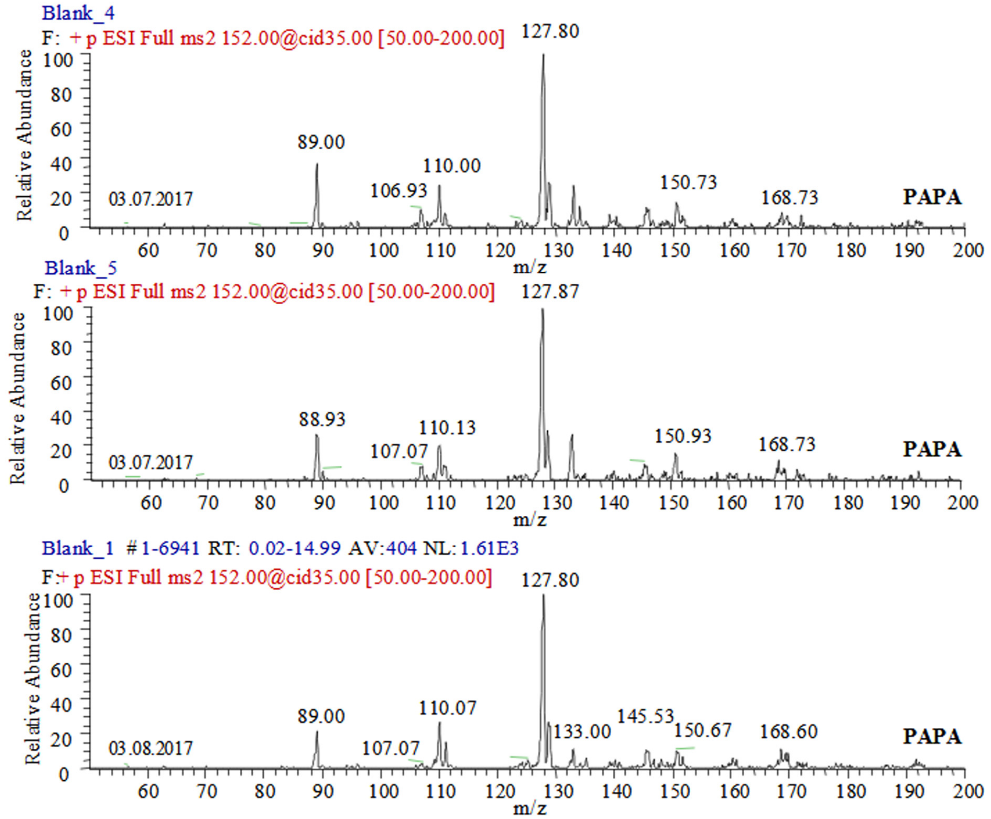

Figure S1. CID-MS/MS spectra of molecular cation $[M+H]^+$ of paracetamol at m/z 152 assessing within-day and between-days variability of mass spectrometric measurands.

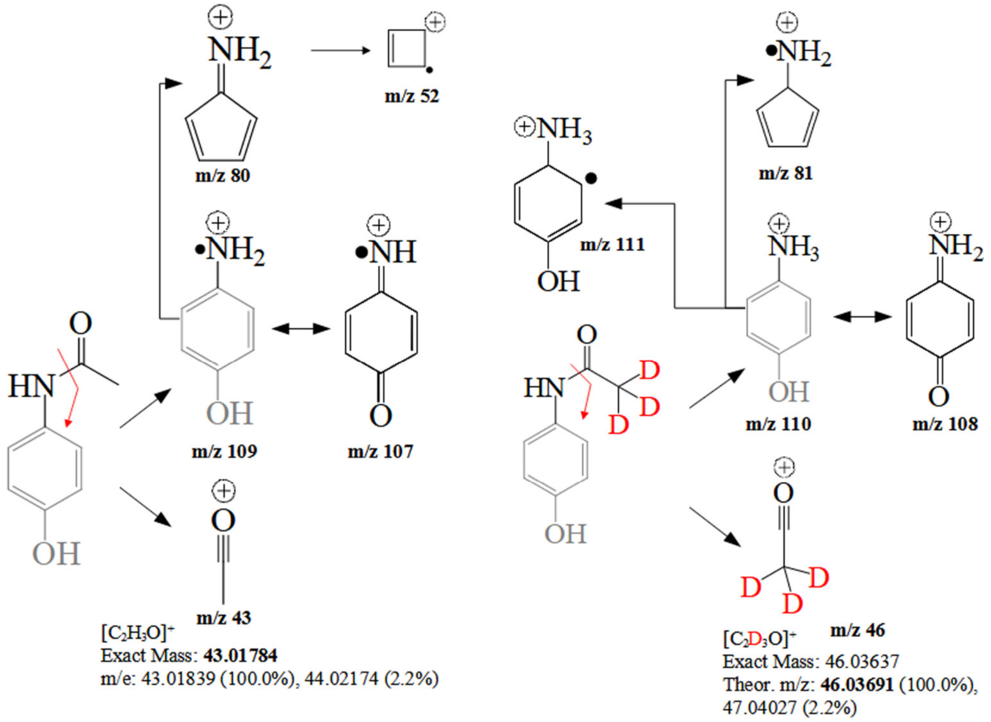

m/z 52
m/z 80
m/z 109
m/z 107
m/z 43
[C2H3O]+
Exact Mass: 43.01784
m/e: 43.01839 (100.0%), 44.02174 (2.2%)
m/z 111
m/z 81
m/z 110
m/z 108
m/z 46
[C2D3O]+
Exact Mass: 46.03637
Theor. m/z: 46.03691 (100.0%), 47.04027 (2.2%)

(Continued)

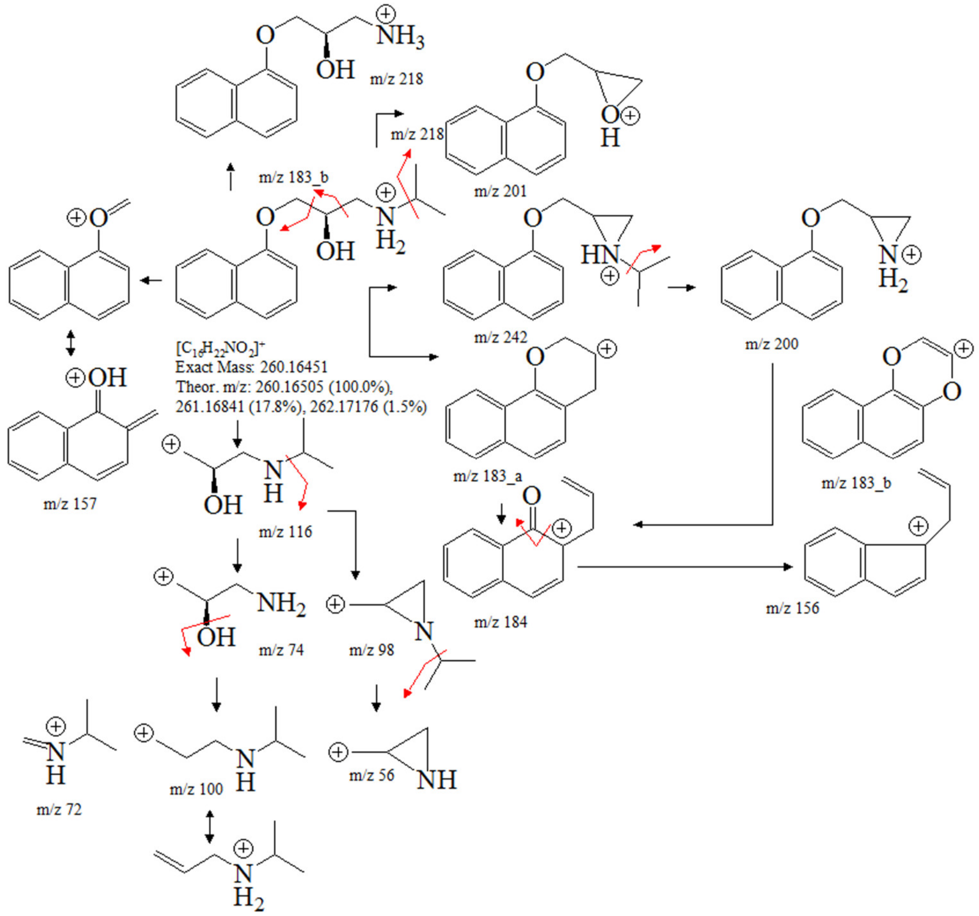
m/z 218
m/z 218
m/z 183_b
m/z 201
m/z 242
m/z 200
[C16H22NO2]+
Exact Mass: 260.16451
Theor. m/z: 260.16505 (100.0%), 261.16841 (17.8%), 262.17176 (1.5%)
m/z 157
m/z 116
m/z 183_a
m/z 183_b
m/z 184
m/z 156
m/z 74
m/z 98
m/z 72
m/z 100
m/z 56

(Continued)

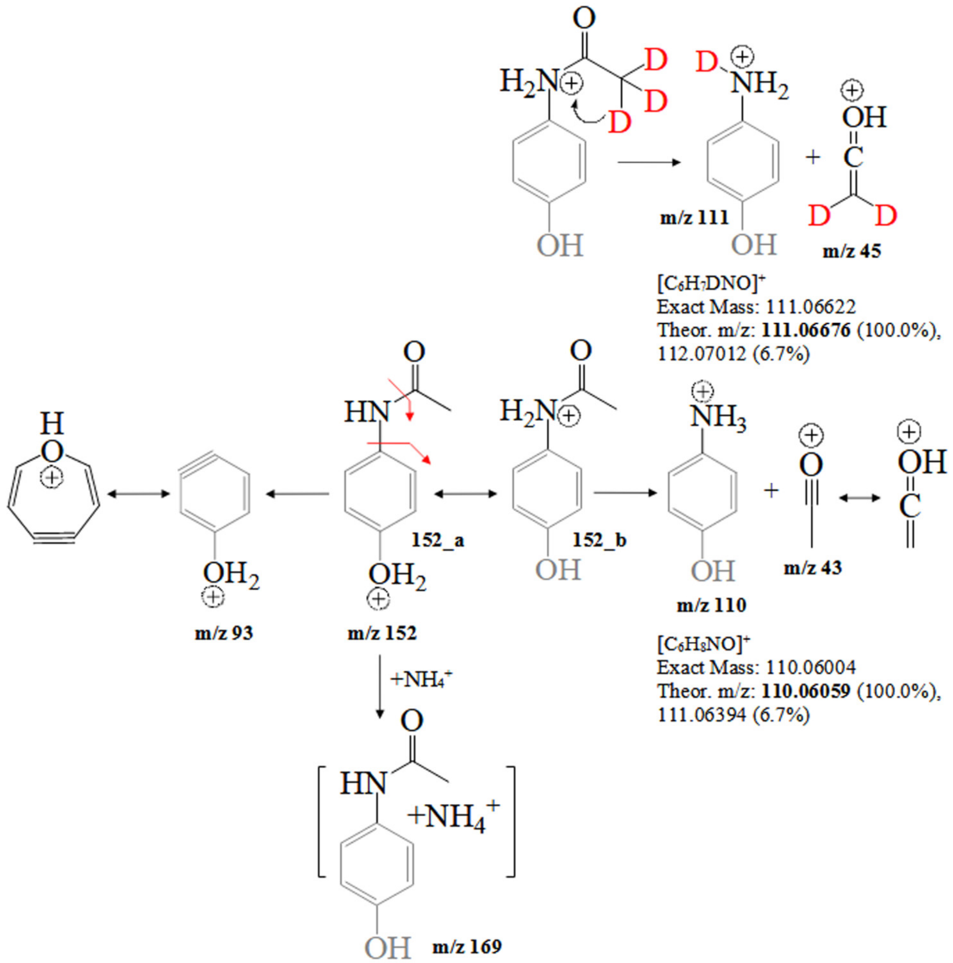

Figure S2. Fragmentation scheme of paracetamol and its d_3-derivative according to [77]; proposed thautomeric equilibria of ions at m/z 110/108, 109/107, 111, 80, 52, 46 and 43 of cations and cation-radicals; exact mass and theoretical m/z data on mass spectrometric isotope shape of the species; intensity ratio of the isotope mass spectrometric shape peaks***;*** Fragmentation scheme of paracetamol and its d_3-derivative according to [77]; proposed thautomeric equilibria of ions at m/z 169, 152, 111/110, 93, 45 and 43 of cations and cation-radicals; exact mass and theoretical m/z data on mass spectrometric isotope shape of the species; intensity ratio of the isotope mass spectrometric shape peaks; Chemical diagrams of fragmentation ions of propranolol according to [80].

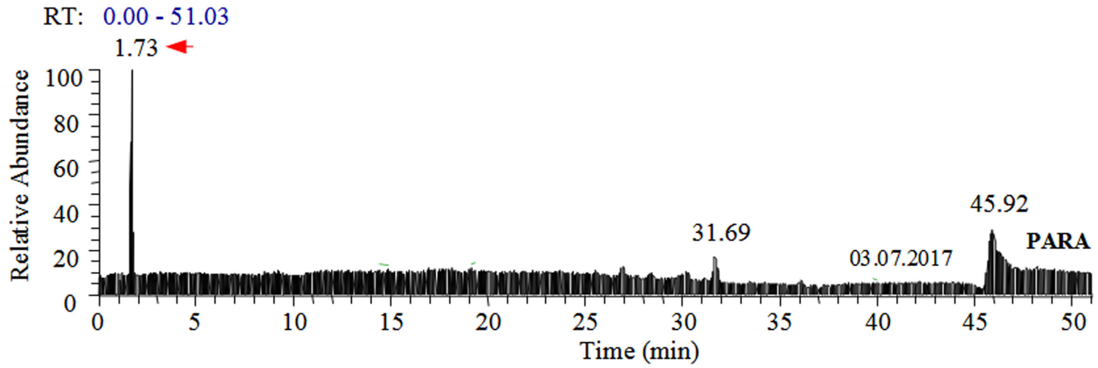
RT: 0.00 - 51.03
1.73
31.69
45.92
03.07.2017
PARA
Relative Abundance
Time (min)

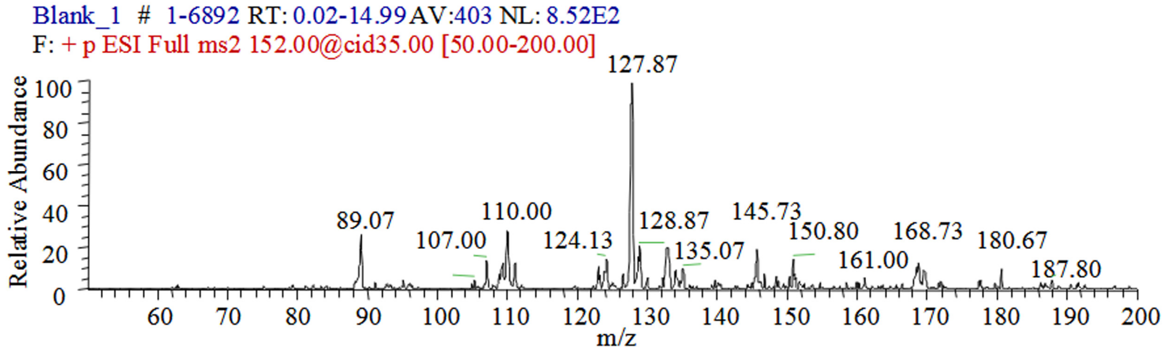
Blank_1 # 1-6892 RT: 0.02-14.99 AV:403 NL: 8.52E2
F: + p ESI Full ms2 152.00@cid35.00 [50.00-200.00]
127.87
89.07
107.00
110.00
124.13
128.87
135.07
145.73
150.80
161.00
168.73
180.67
187.80
Relative Abundance
m/z

(Continued)

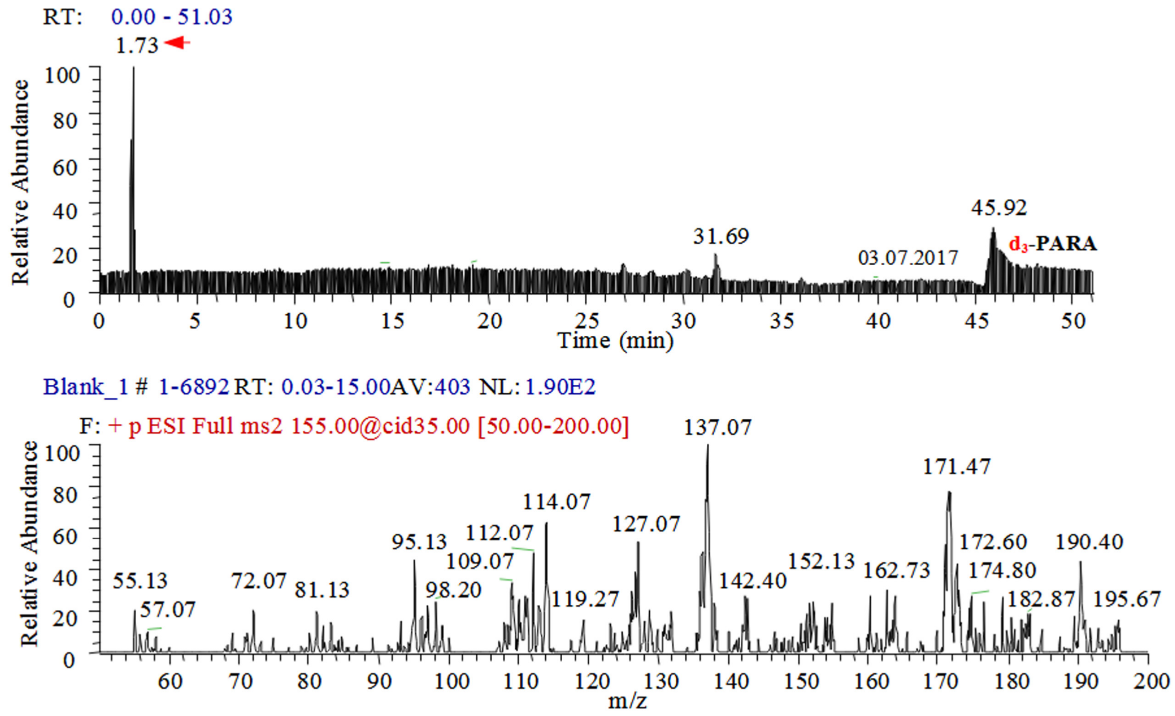

RT: 0.00 - 51.03
1.73
Relative Abundance
31.69
03.07.2017
45.92
d3-PARA
Time (min)
Blank_1 # 1-6892 RT: 0.03-15.00 AV: 403 NL: 1.90E2
F: + p ESI Full ms2 155.00@cid35.00 [50.00-200.00]
137.07
171.47
114.07
127.07
95.13
112.07
109.07
98.20
55.13
57.07
72.07
81.13
119.27
142.40
152.13
162.73
172.60
174.80
182.87
190.40
195.67
m/z

(Continued)

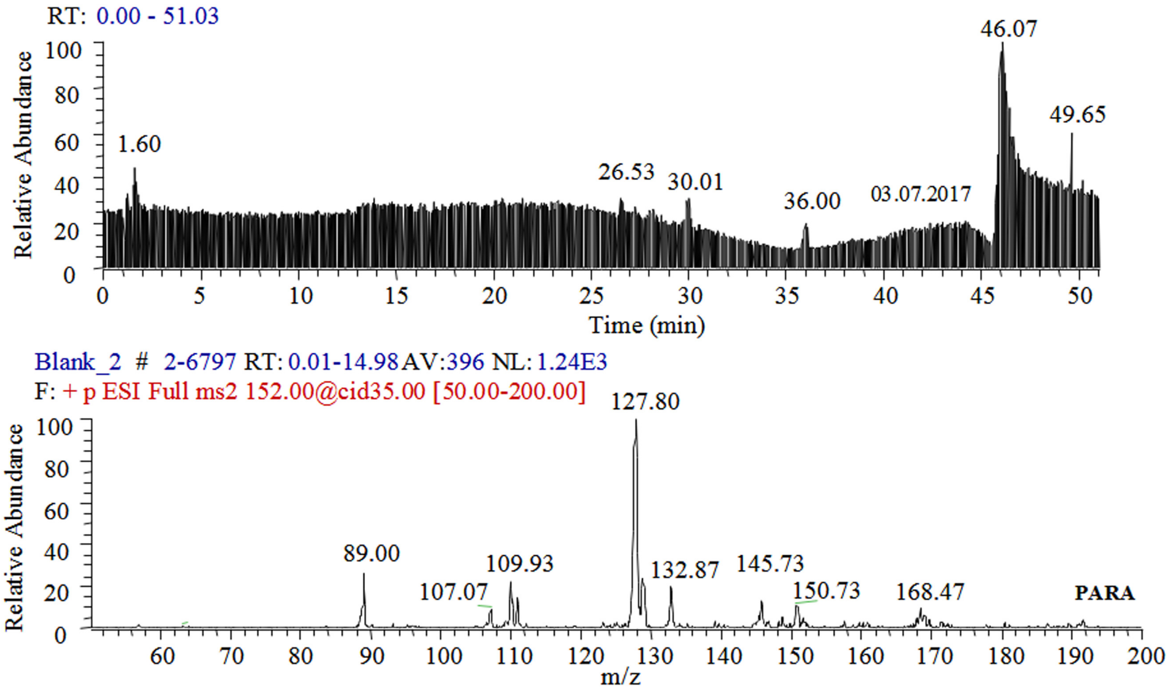
RT: 0.00 - 51.03
Relative Abundance
Time (min)
1.60
26.53
30.01
36.00
03.07.2017
46.07
49.65
Blank_2 # 2-6797 RT: 0.01-14.98 AV:396 NL: 1.24E3
F: + p ESI Full ms2 152.00@cid35.00 [50.00-200.00]
Relative Abundance
m/z
127.80
89.00
107.07
109.93
132.87
145.73
150.73
168.47
PARA

(Continued)

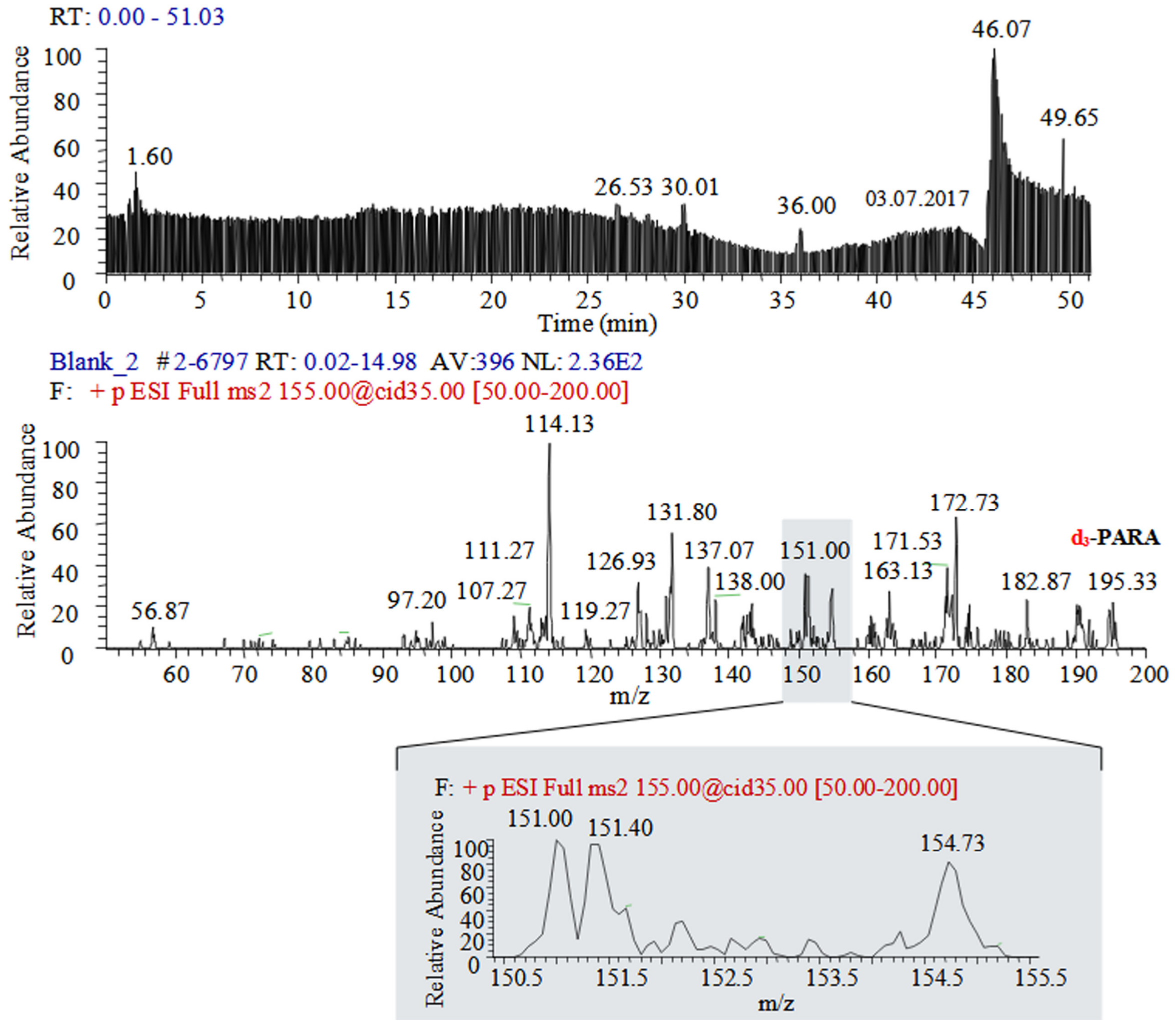
RT: 0.00 - 51.03
Relative Abundance
Time (min)
1.60
26.53 30.01
36.00
03.07.2017
46.07
49.65
Blank_2 #2-6797 RT: 0.02-14.98 AV:396 NL: 2.36E2
F: + p ESI Full ms2 155.00@cid35.00 [50.00-200.00]
m/z
114.13
131.80
172.73
111.27
126.93
137.07
151.00
171.53
163.13
138.00
97.20
107.27
119.27
182.87
195.33
56.87
d3-PARA
F: + p ESI Full ms2 155.00@cid35.00 [50.00-200.00]
151.00
151.40
154.73

(Continued)

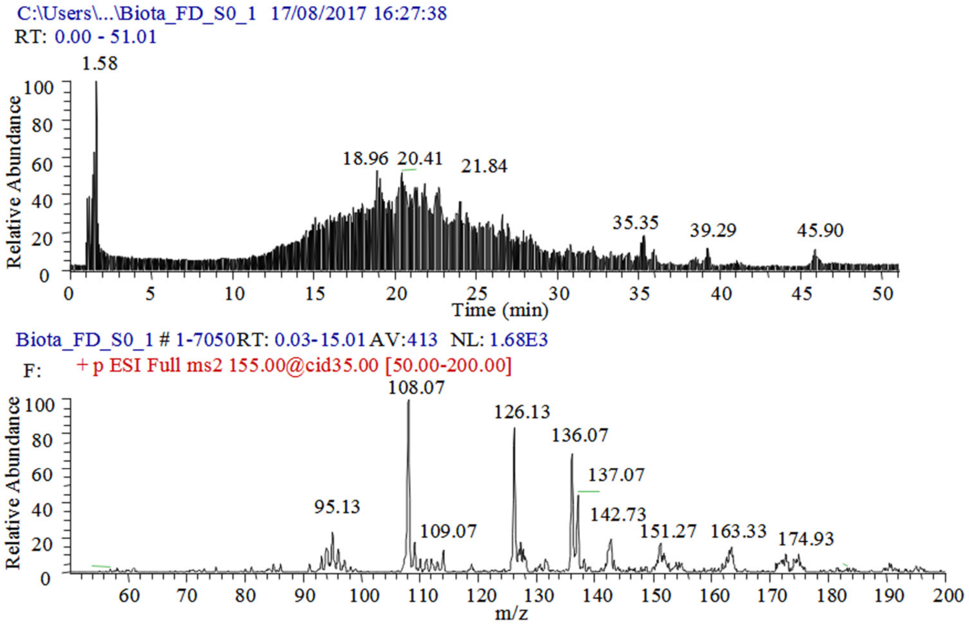

Figure S3. Total ion current and tandem MS/MS spectra of blank samples of paracetamol and its d_3-derivative using molecular cations $[M+H]^+$ and $[d_3\text{-}M+H]^+$ at m/z 152 and 155.

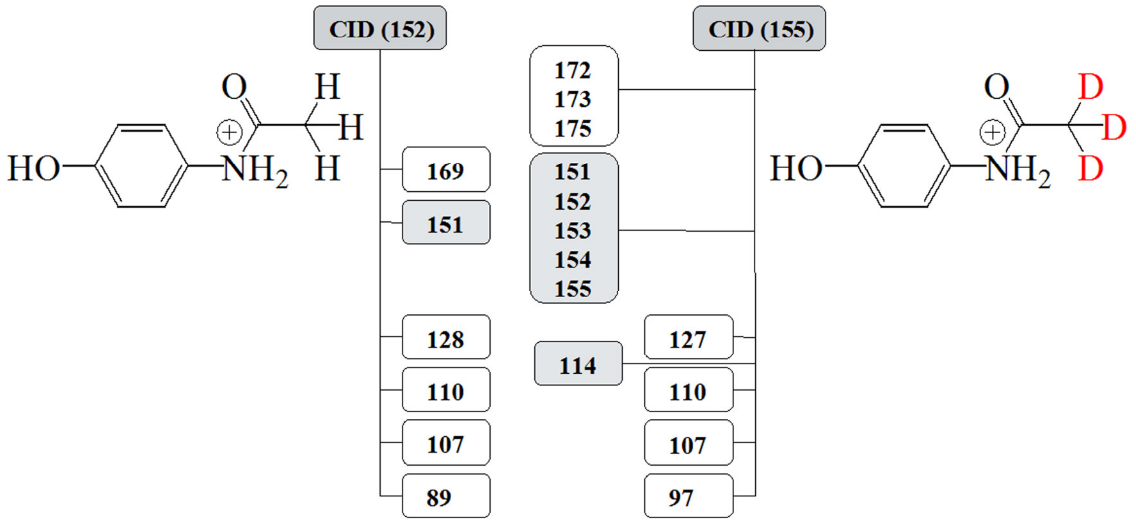

Figure S4. Chemical diagrams of paracetamol and its d$_3$-derivative; selected fragmentation ions of CID-MS/MS spectra of molecular $[M+H]^+$ cation at m/z 152 and 155.

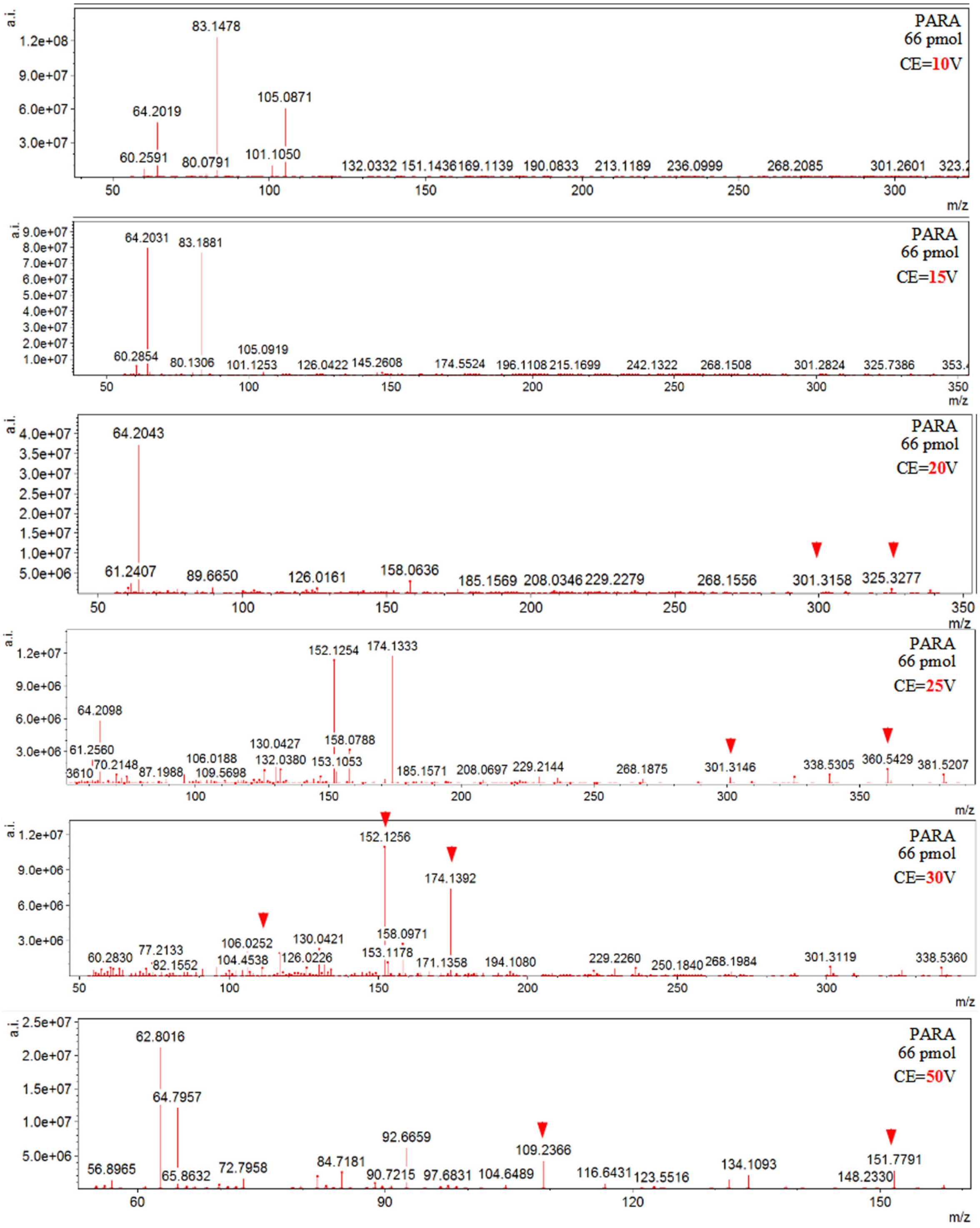

Figure S5. ESI(+)-MS/MS tandem mass spectra of molecular ion $[M+H]^+$ of standard sample paracetamol at m/z 152 depending on collision energy (CE) ranging from 10–50 V.

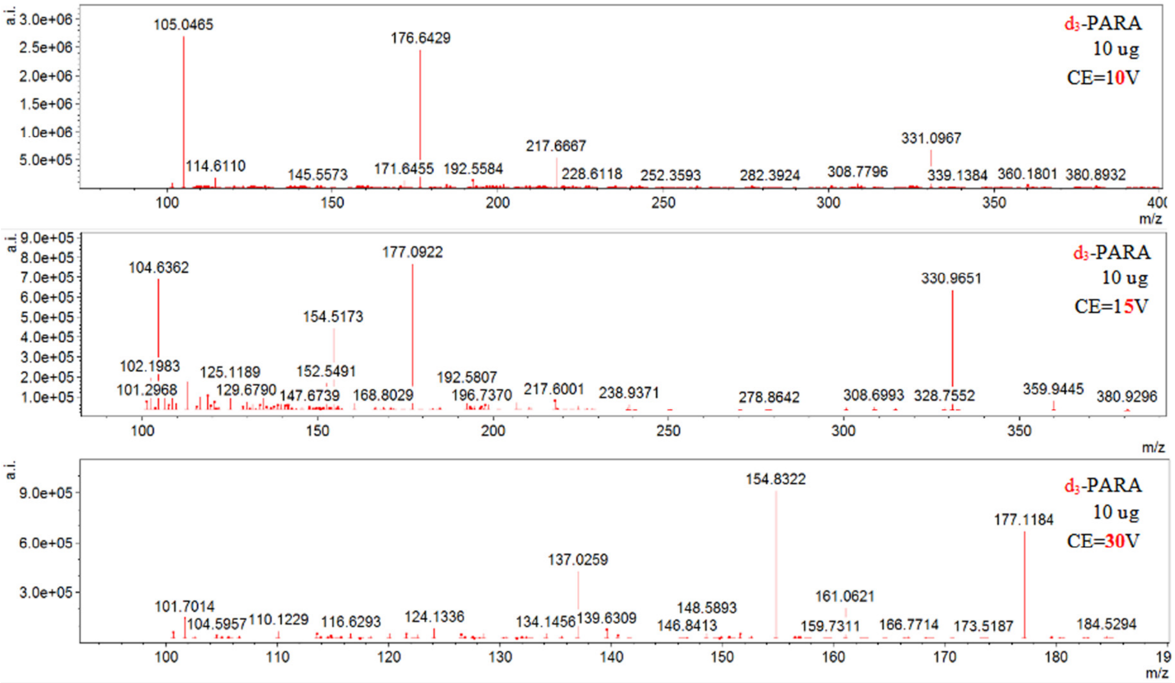

Figure S6. ESI(+)-MS/MS tandem mass spectra of molecular ion $[M+H]^+$ of standard sample of d_3-paracetamol at m/z 155 depending on collision energy (CE) ranging from 10–30 V.

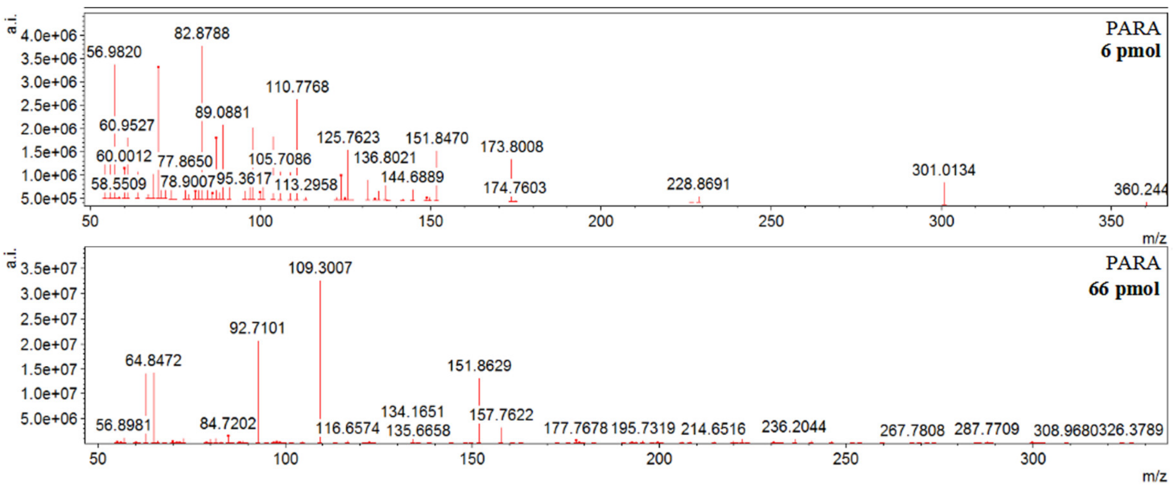

Figure S7. ESI(+)-MS/MS tandem mass spectra of molecular ion $[M+H]^+$ of standard sample paracetamol at m/z 152 depending analyte concentration (6 and 66 pmol).

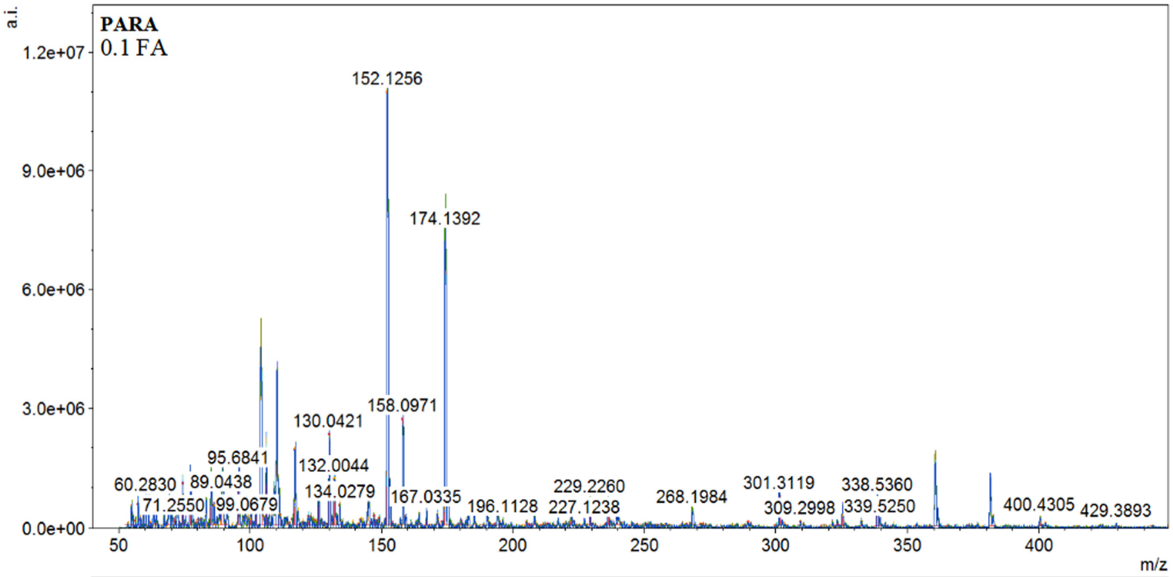
PARA
0.1 FA
a.i.
1.2e+07
9.0e+06
6.0e+06
3.0e+06
0.0e+00
152.1256
174.1392
158.0971
130.0421
95.6841
132.0044
60.2830
89.0438
71.2550
99.0679
134.0279
167.0335
196.1128
229.2260
227.1238
268.1984
301.3119
309.2998
338.5360
339.5250
400.4305
429.3893
50
100
150
200
250
300
350
400
m/z

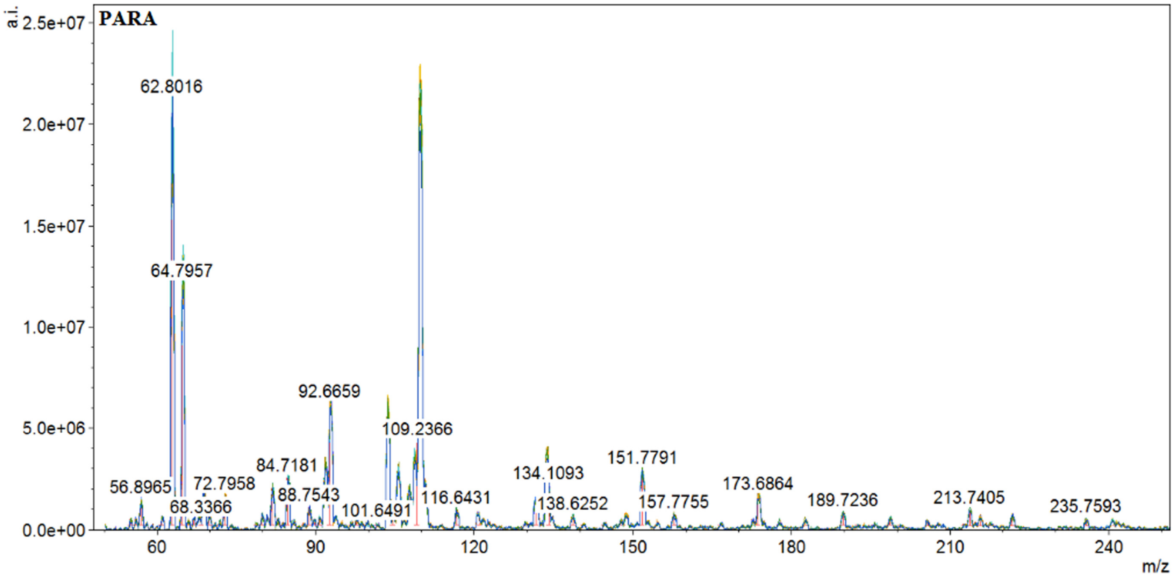
PARA
a.i.
2.5e+07
2.0e+07
1.5e+07
1.0e+07
5.0e+06
0.0e+00
62.8016
64.7957
92.6659
109.2366
151.7791
134.1093
84.7181
72.7958
56.8965
88.7543
68.3366
101.6491
116.6431
138.6252
157.7755
173.6864
189.7236
213.7405
235.7593
60
90
120
150
180
210
240
m/z

(Continued)

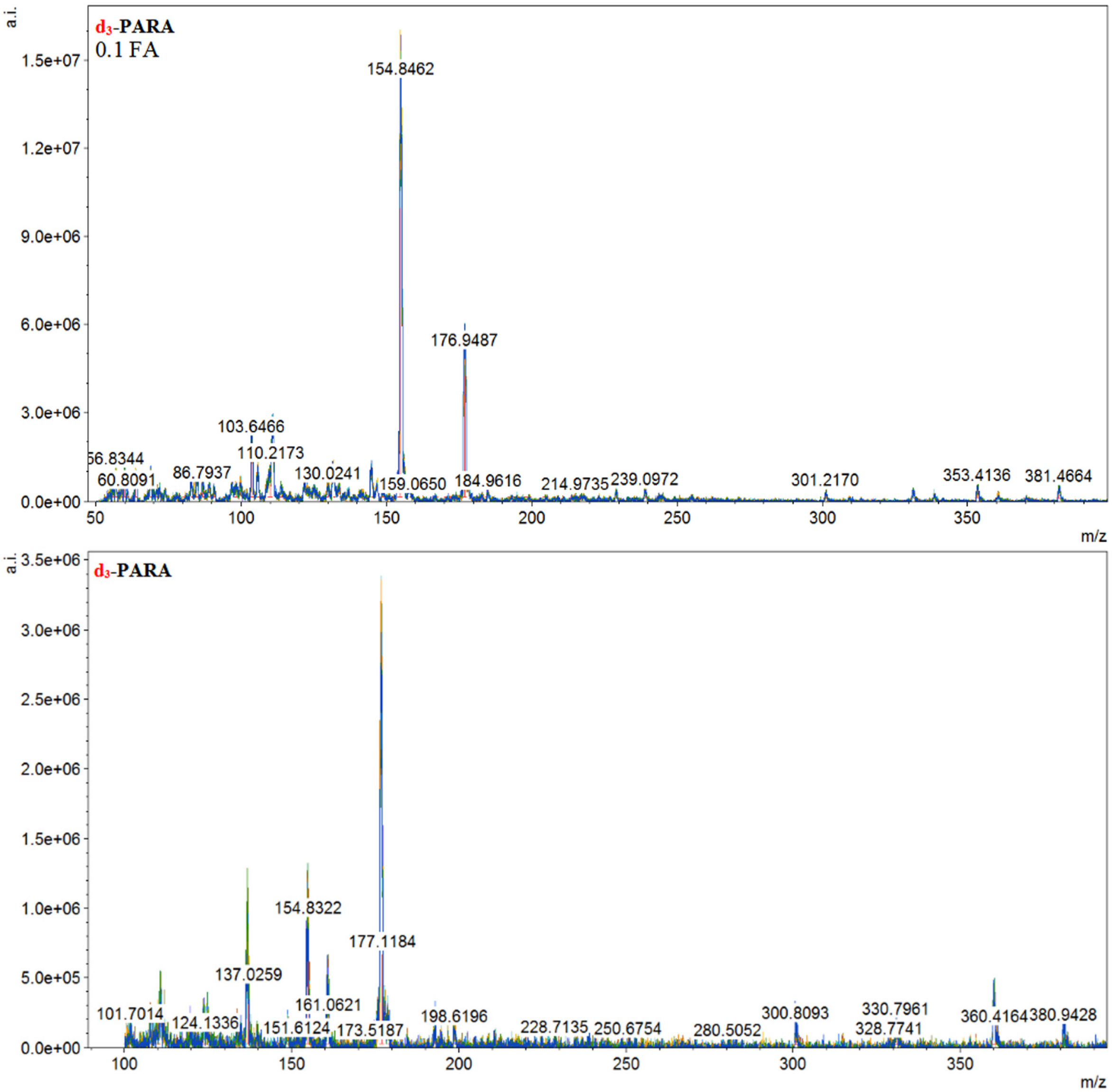

Figure S8. ESI(+)-MS/MS tandem mass spectra of molecular ion $[M+H]^+$ of standard sample of paracetamol and its d_3-derivative at m/z 152 and 155 depending on presence of formic acid (FA).

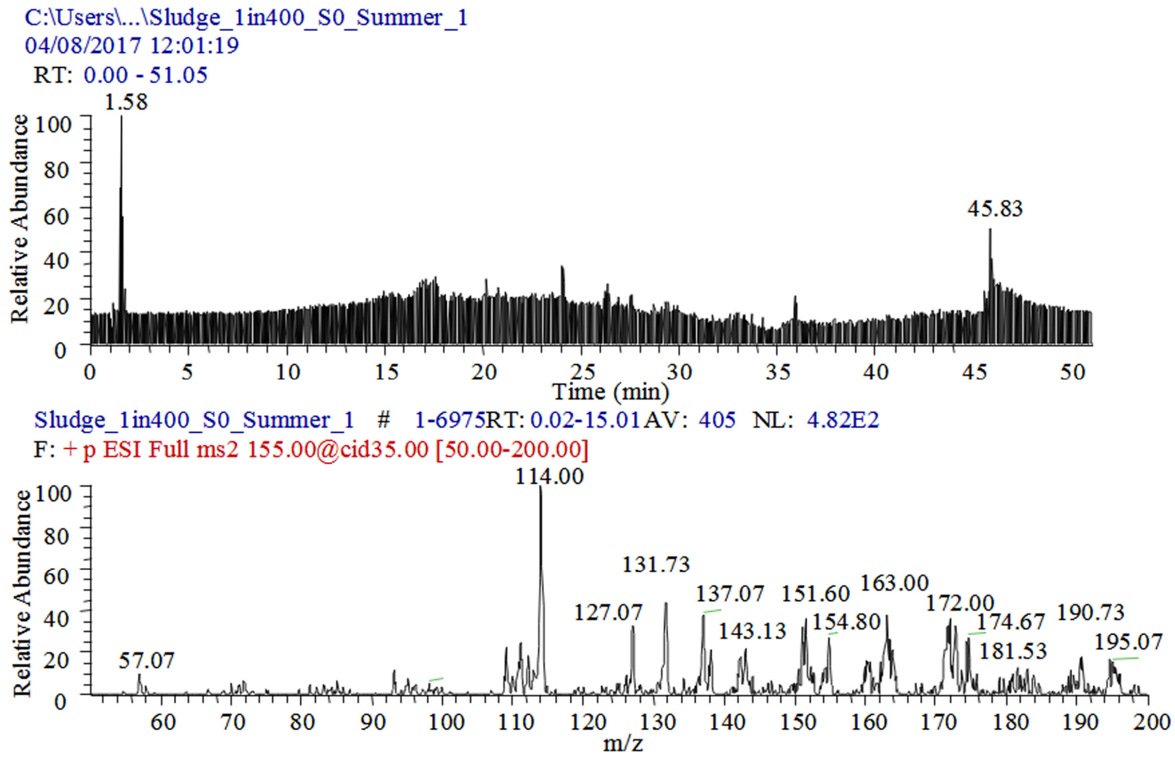

C:\Users\...\Sludge_1in400_S0_Summer_1
04/08/2017 12:01:19
RT: 0.00 - 51.05
1.58
45.83
Relative Abundance
Time (min)
Sludge_1in400_S0_Summer_1 # 1-6975RT: 0.02-15.01AV: 405 NL: 4.82E2
F: + p ESI Full ms2 155.00@cid35.00 [50.00-200.00]
114.00
131.73
127.07
137.07
151.60
163.00
172.00
143.13
154.80
174.67
190.73
181.53
195.07
57.07
Relative Abundance
m/z

(Continued)

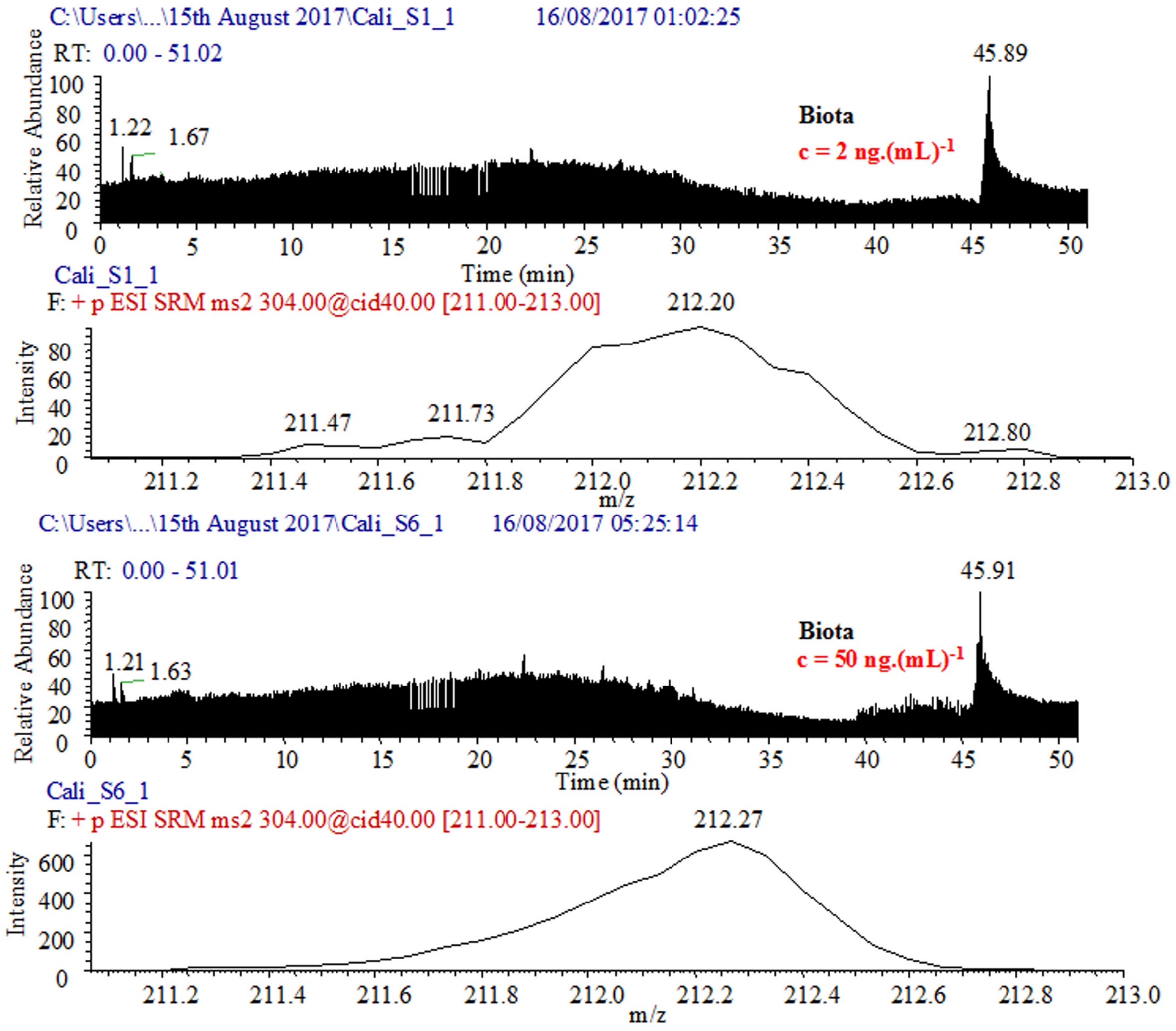

Figure S9. The total ion current for standard sample of treated sludgecake (top); CID-MS/MS spectrum of molecular cation $[M+H]^+$ at m/z 155 of d_3-PARA (bottom;) The total ion current of standard calibration samples of biota depending on analyte BAC-C12 concentration [ng.$(mL)^{-1}$]; CID-MS/MS spectra of molecular cation $[M]^+$ at m/z 304 of BAC-C12.

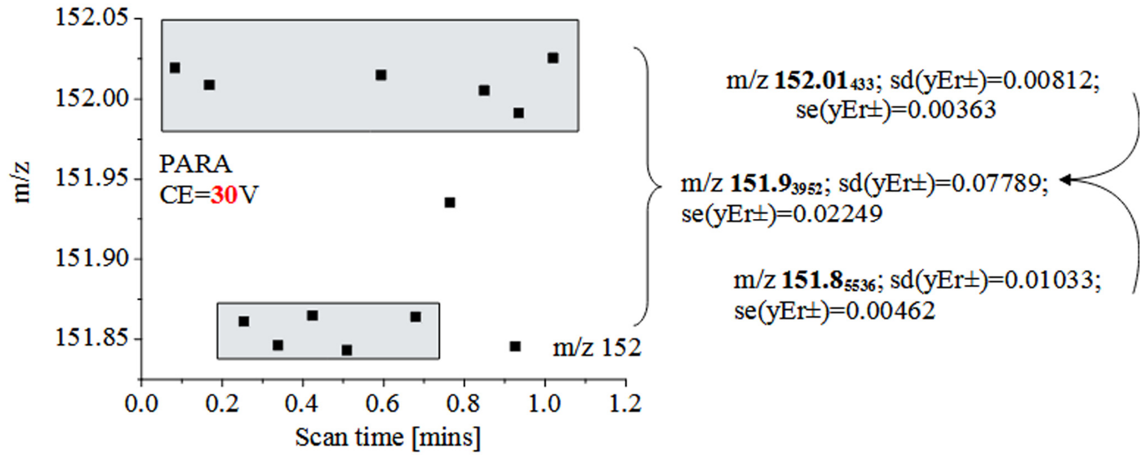

	Value	Error	
A	$4.68.10^{-11}$	$3.024.10^{-12}$	
B	$-2.45.10^{-12}$	$4.43.10^{-13}$	
|r|	SD	N	*p*
**0.98411**	$2.51.10^{-12}$	3	0.912

(Continued)

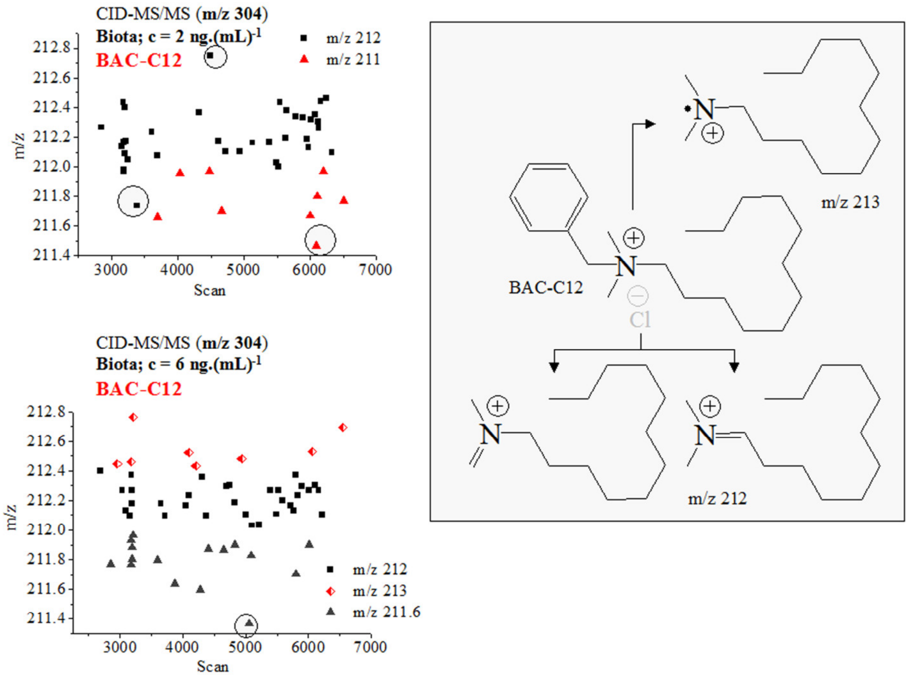

Figure S10. Temporal distribution of m/z measurands of ESI(+)-MS/MS spectrum of molecular ion $[M+H]^+$ at m/z 152 of standard sample paracetamol measured at CE=30V; mean values, standard deviation and standard error of the measurable variables; chemometrics; Temporal distribution of m/z data on fragmentation ions at m/z 211, 212 and 213 of SRM spectra of BAC-C12 of its molecular cation $[M]^+$ at m/z 304; chemical diagrams of BAC-C12 and its major fragmentation product ions.

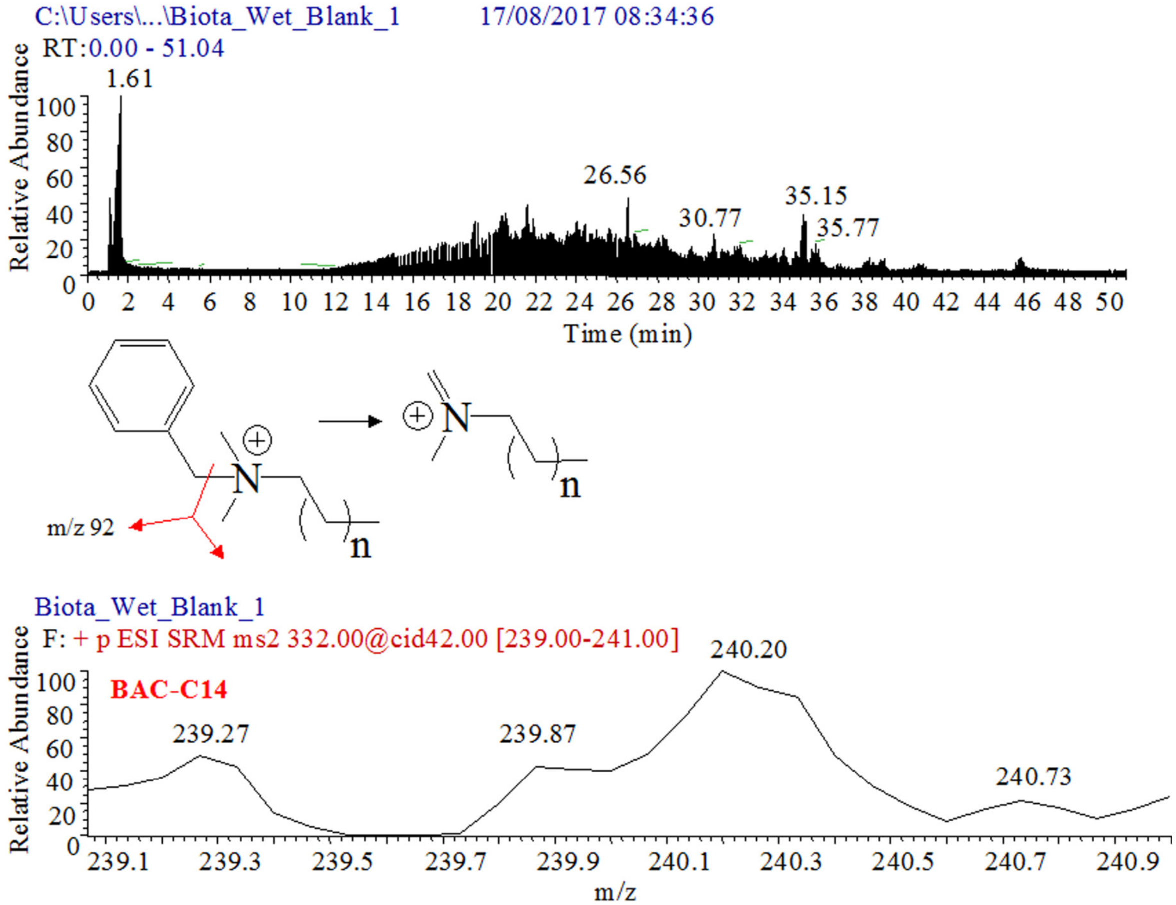

Figure S11. The total ion current of blank wet sample of biota (top); SRM spectrum of molecular cation $[M+H]^+$ at m/z 332 of BAC-C14 (bottom;) chemical diagram of common fragmentation path of benzalkonium cations.

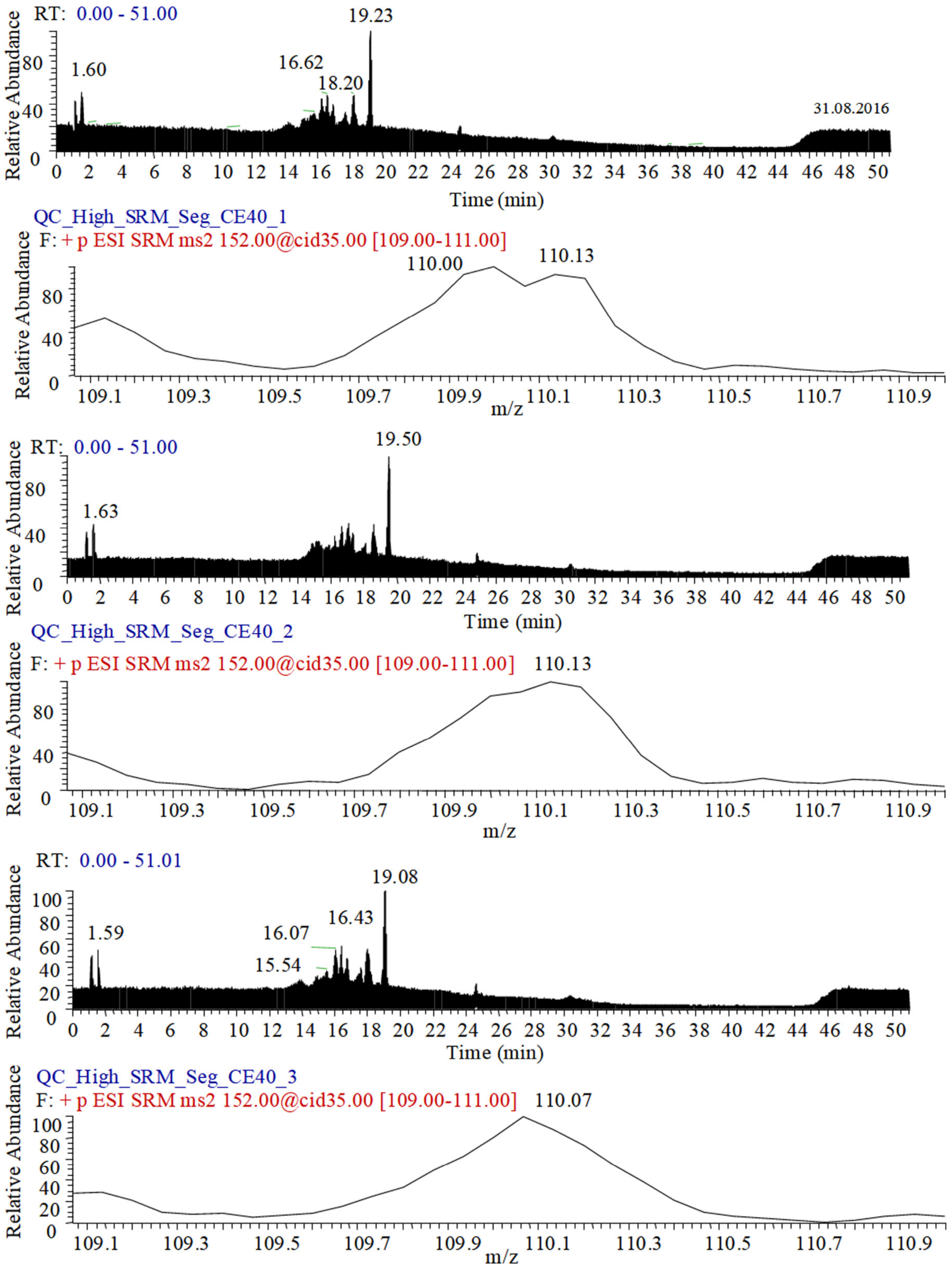

Figure S12. Chromatographic data and segment SRM spectra of molecular ion $[M+H]^+$ of paracetamol at m/z 152 (i=–3) measured at Δt=1.2 s and CE = 40 V.

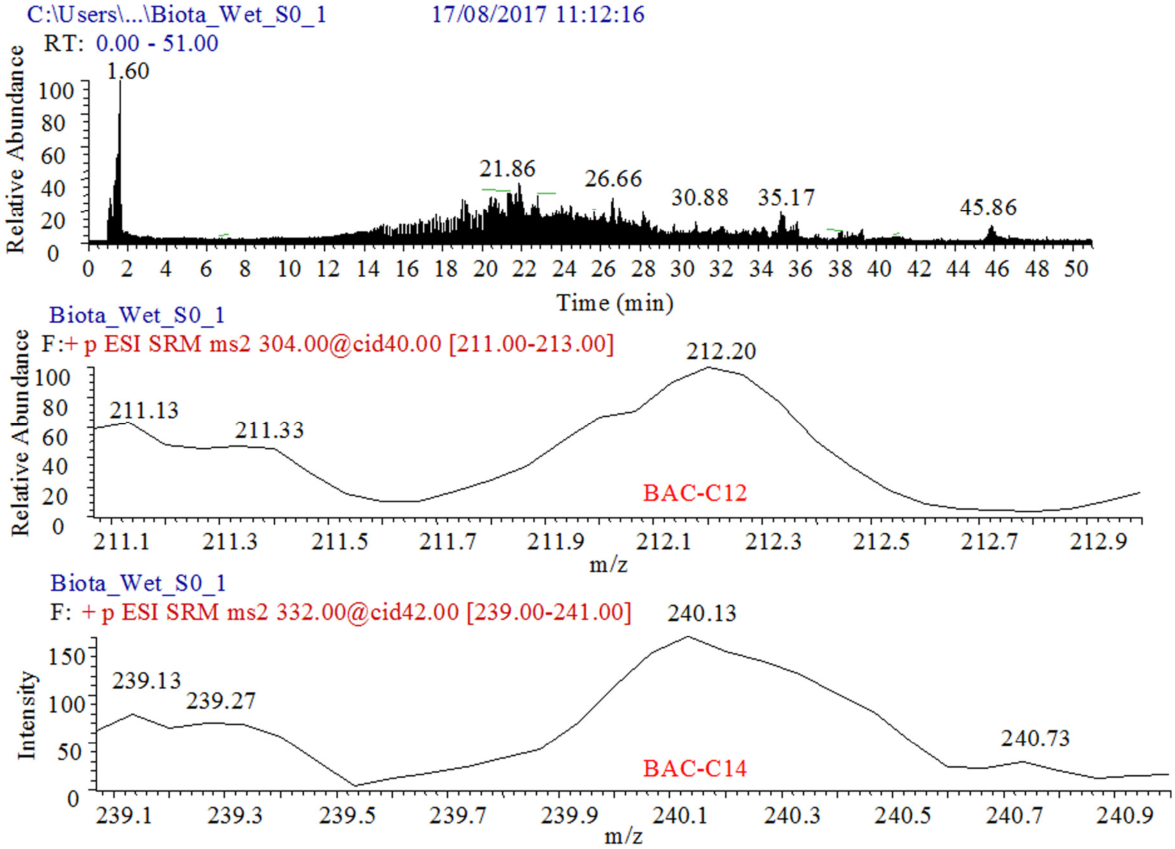

Figure S13. Chromatographic data and segment SRM spectra of molecular ion $[M+H]^+$ of BAC-C12 and BAC-C14 at m/z 304 and 332 of wet biota sample.

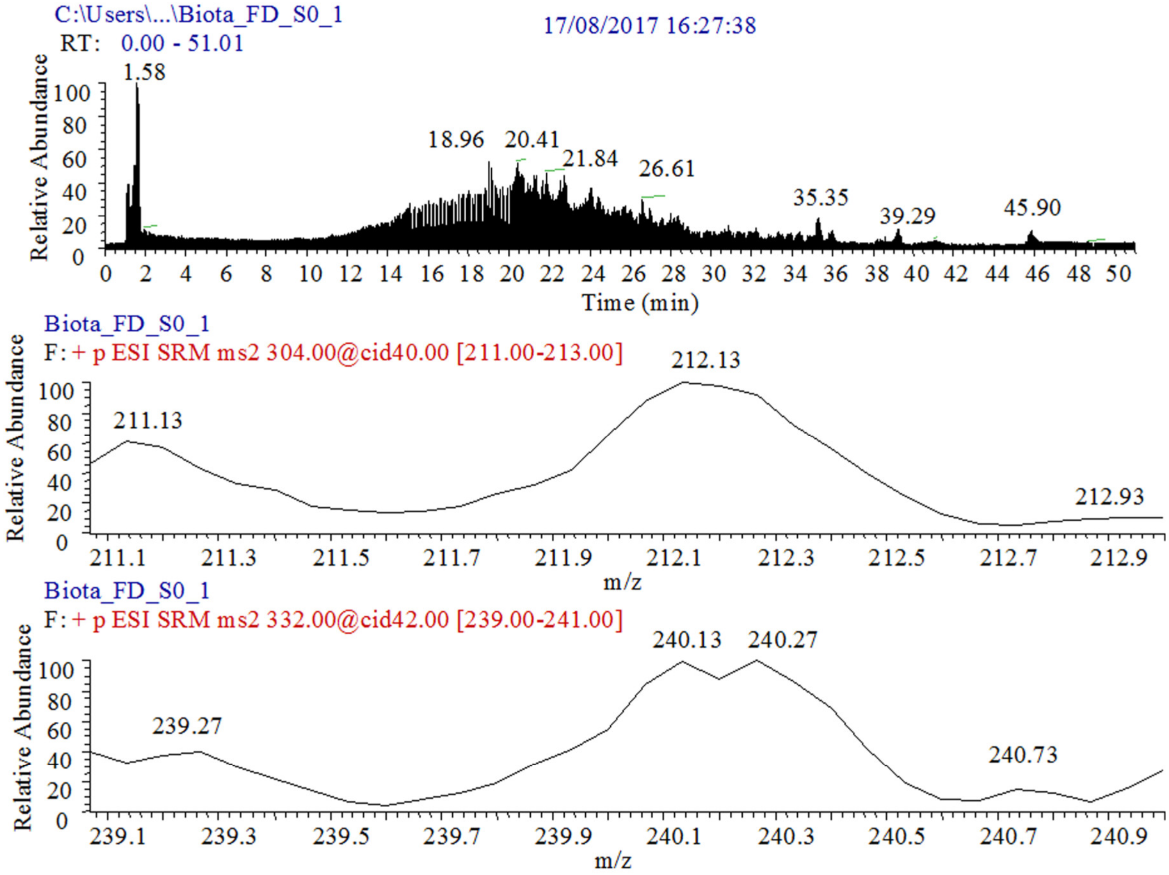

Figure S14. Chromatographic data and segment SRM spectra of molecular ion $[M+H]^+$ of BAC-C12 and BAC-C14 at m/z 304 and 332 of freeze-dried biota sample.

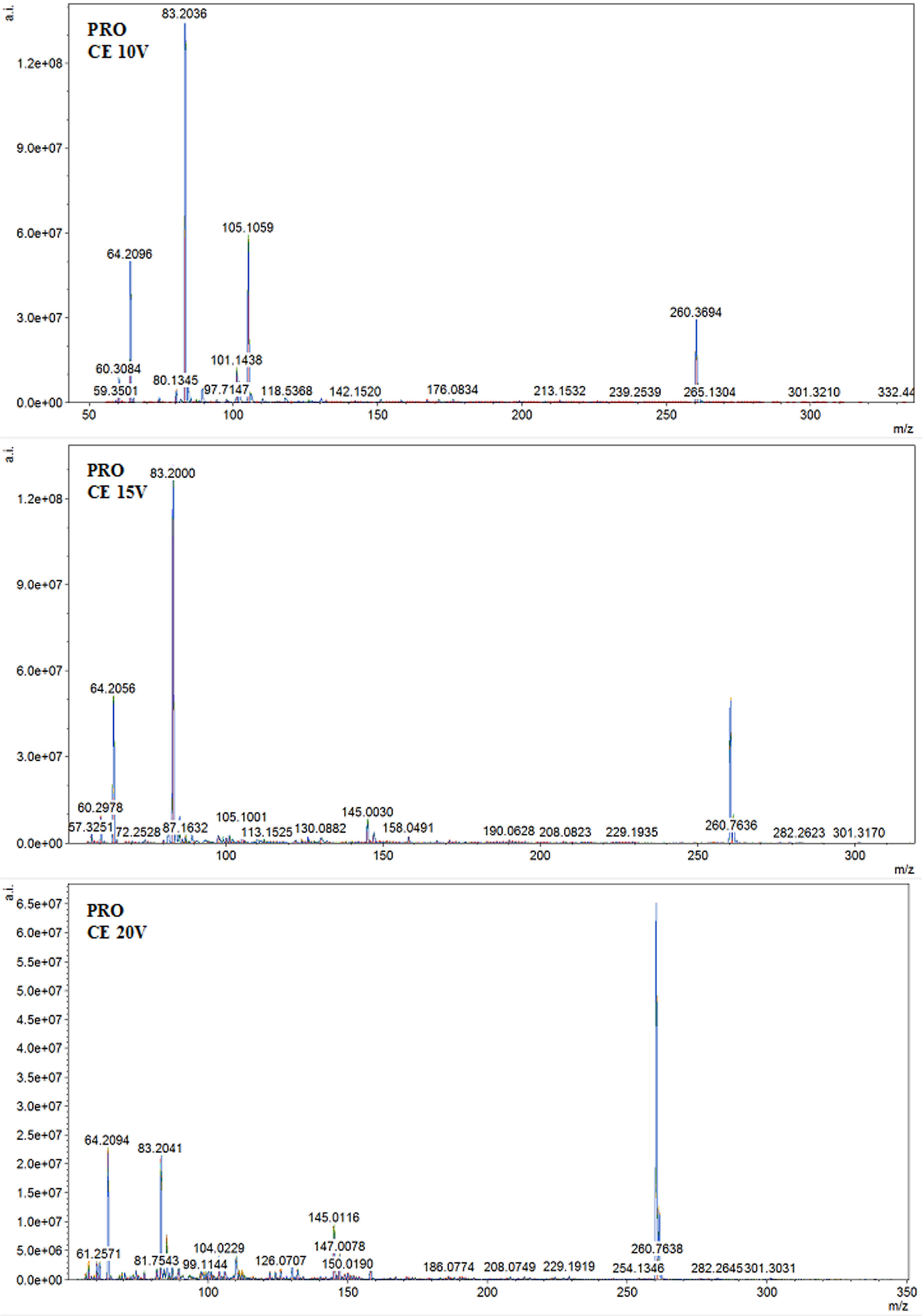
PRO
CE 10V
a.i.
83.2036
105.1059
64.2096
260.3694
60.3084
101.1438
59.3501
80.1345
97.7147
118.5368
142.1520
176.0834
213.1532
239.2539
265.1304
301.3210
m/z
PRO
CE 15V
83.2000
64.2056
60.2978
57.3251
72.2528
87.1632
105.1001
113.1525
130.0882
145.0030
158.0491
190.0628
208.0823
229.1935
260.7636
282.2623
301.3170
PRO
CE 20V
64.2094
83.2041
145.0116
147.0078
150.0190
104.0229
61.2571
81.7543
99.1144
126.0707
186.0774
208.0749
229.1919
260.7638
254.1346
282.2645
301.3031

(Continued)

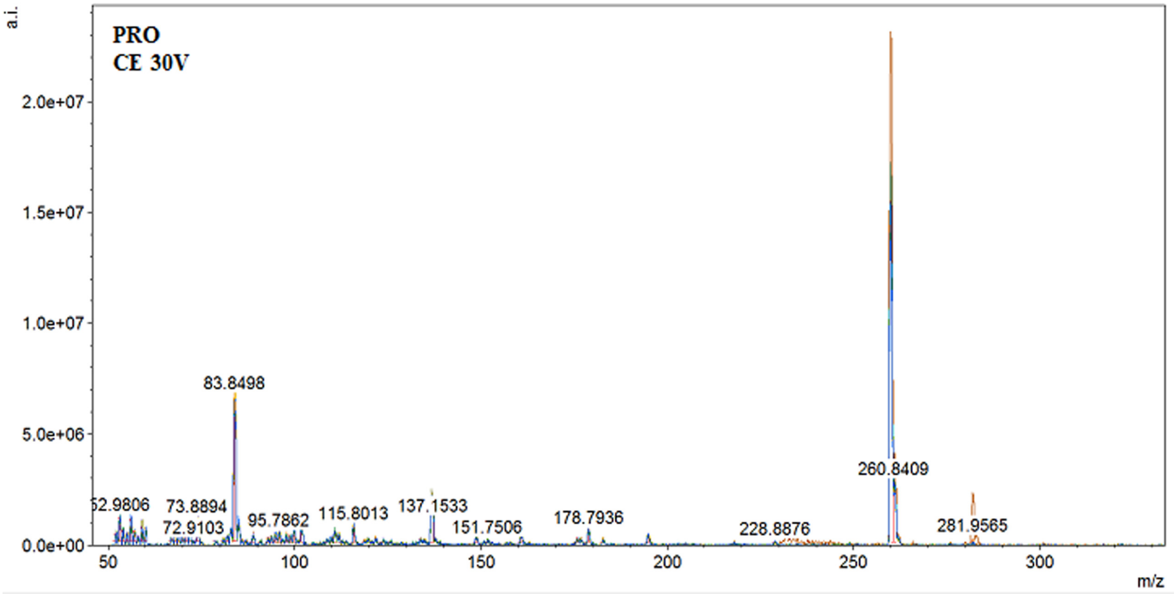

Figure S15. ESI(+)-MS spectra of standard sample propranolol, depending on collision energy (CE) ranging from 10–30 V.

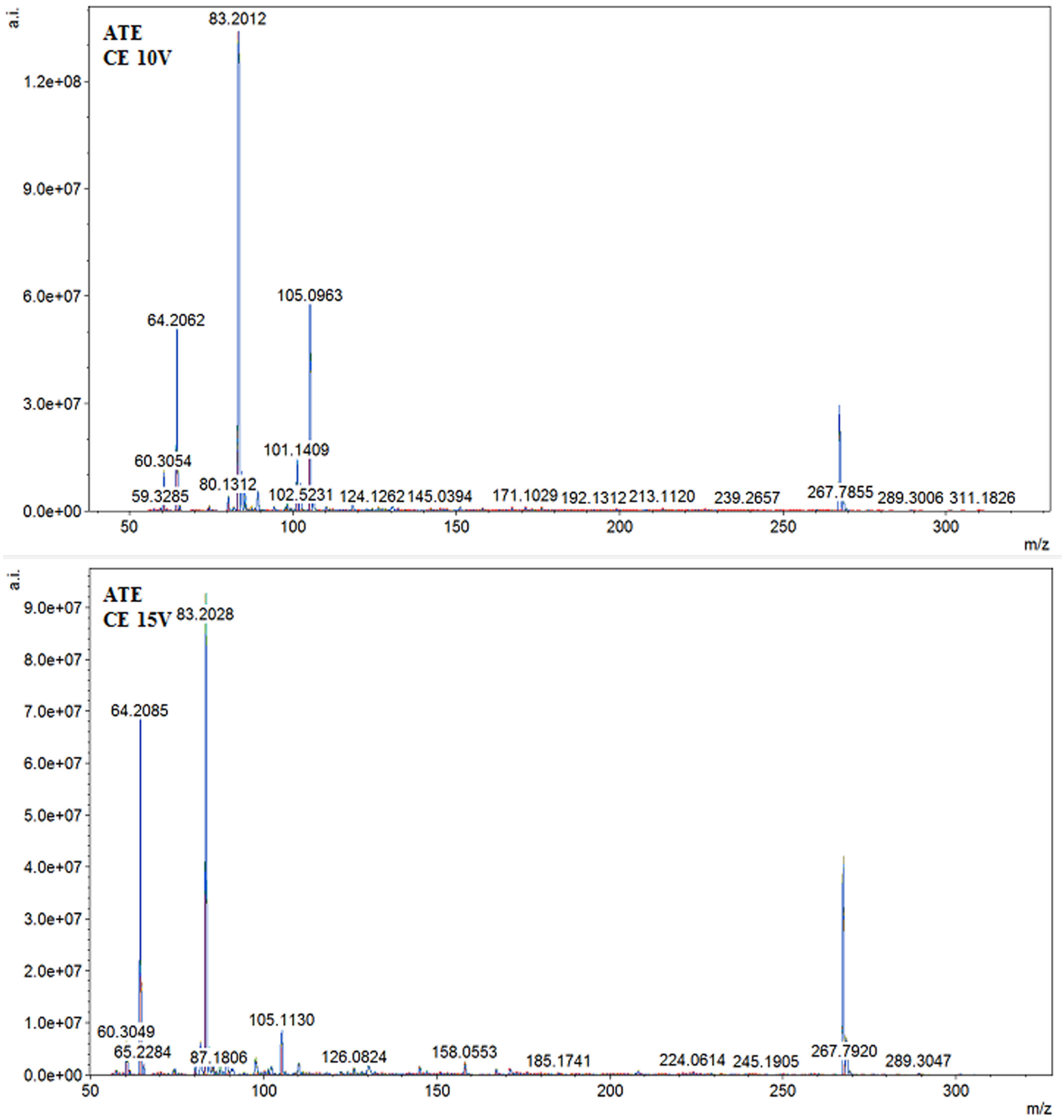
ATE
CE 10V
a.i.
1.2e+08
9.0e+07
6.0e+07
3.0e+07
0.0e+00
83.2012
105.0963
64.2062
101.1409
60.3054
80.1312
59.3285
102.5231
124.1262
145.0394
171.1029
192.1312
213.1120
239.2657
267.7855
289.3006
311.1826
50
100
150
200
250
300
m/z
ATE
CE 15V
a.i.
9.0e+07
8.0e+07
7.0e+07
6.0e+07
5.0e+07
4.0e+07
3.0e+07
2.0e+07
1.0e+07
0.0e+00
83.2028
64.2085
105.1130
60.3049
65.2284
87.1806
126.0824
158.0553
185.1741
224.0614
245.1905
267.7920
289.3047
50
100
150
200
250
300
m/z

(Continued)

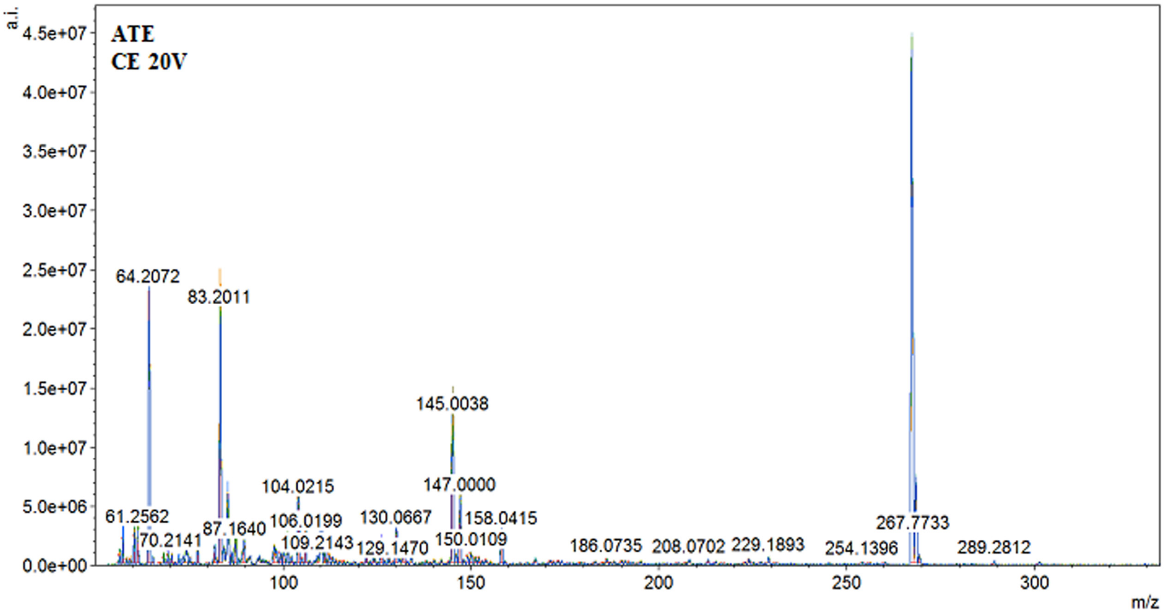
ATE
CE 20V
a.i.
m/z
64.2072
83.2011
145.0038
104.0215
147.0000
61.2562
106.0199
130.0667
158.0415
267.7733
70.2141
87.1640
109.2143
129.1470
150.0109
186.0735
208.0702
229.1893
254.1396
289.2812

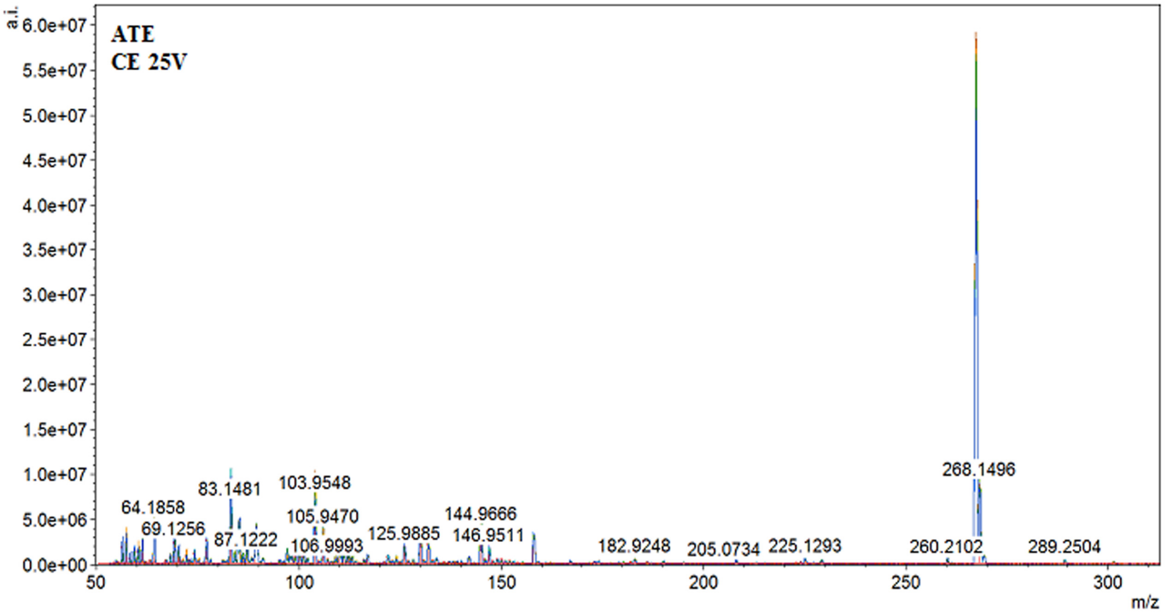
ATE
CE 25V
a.i.
m/z
268.1496
83.1481
103.9548
64.1858
105.9470
144.9666
69.1256
87.1222
106.9993
125.9885
146.9511
182.9248
205.0734
225.1293
260.2102
289.2504

(Continued)

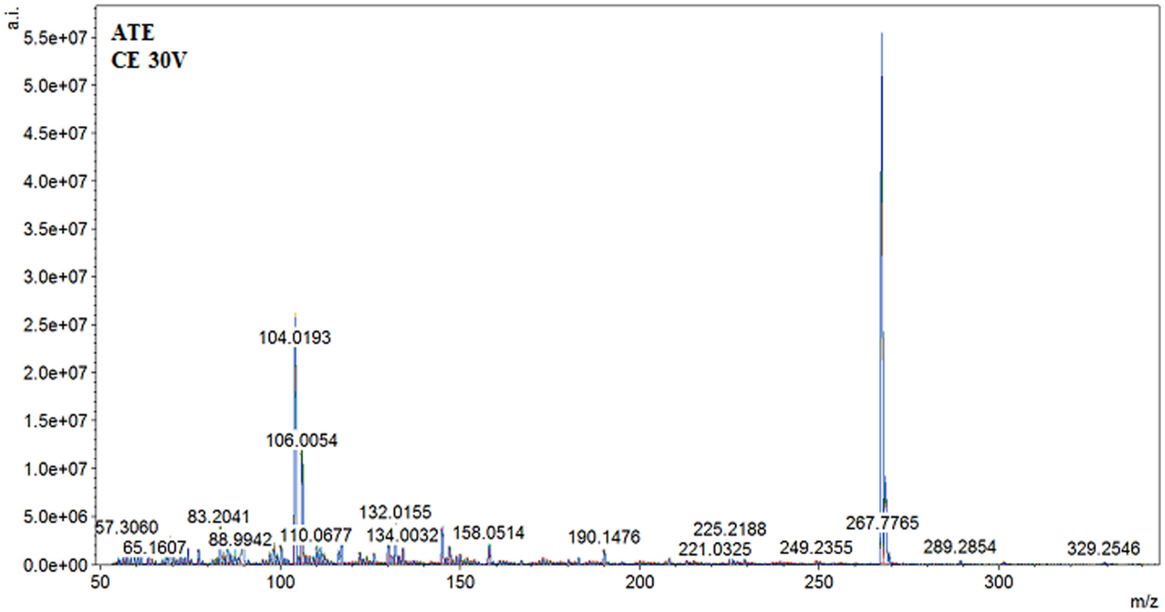

Figure S16. ESI(+)-MS spectra of standard sample atenolol, depending on collision energy (CE) ranging from 10–30 V.

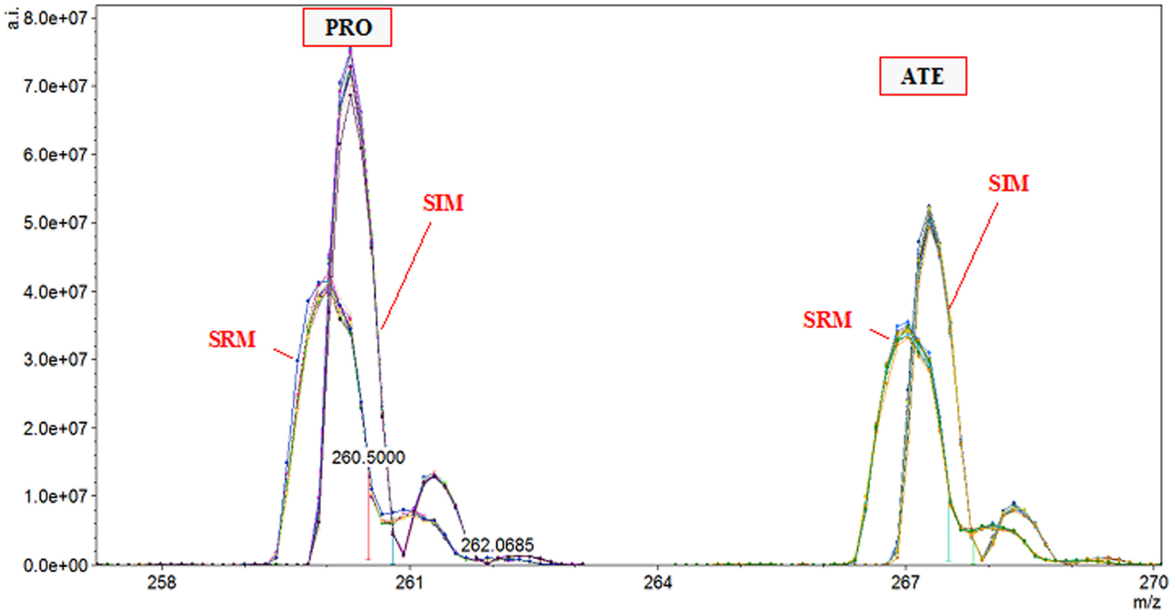

Figure S17. SRM and SIM mass spectra of standard samples of propranolol and atenolol of molecular cation $[M+H]^+$ at m/z 260 and 267.

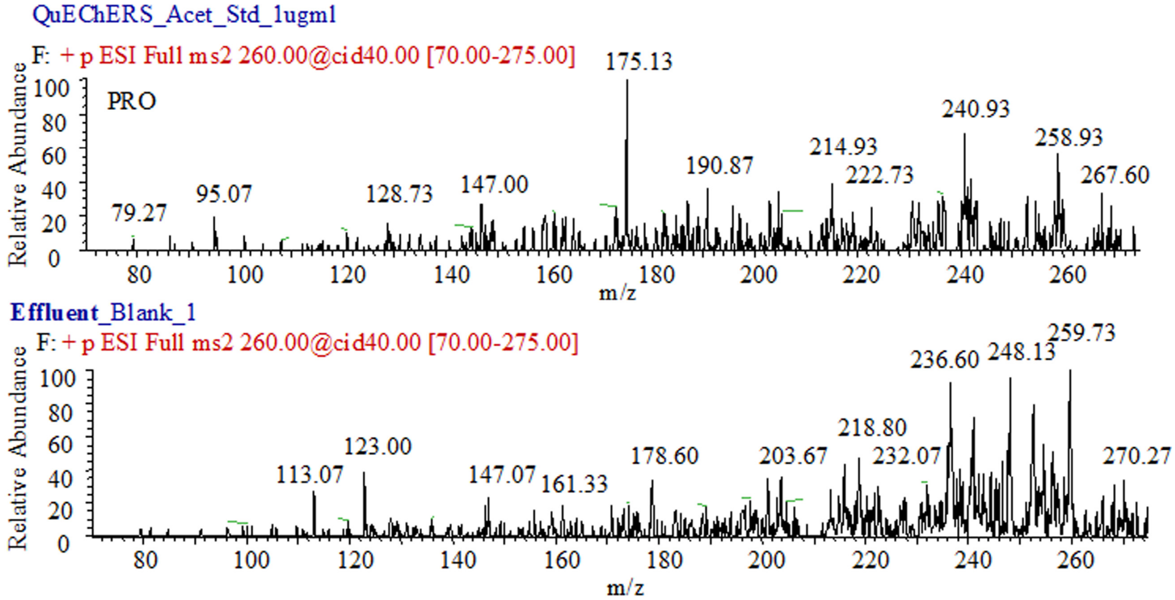
QuEChERS_Acet_Std_1ugml
F: + p ESI Full ms2 260.00@cid40.00 [70.00-275.00]
PRO
Relative Abundance
m/z
175.13
240.93
258.93
214.93
222.73
267.60
190.87
147.00
128.73
95.07
79.27
Effluent_Blank_1
F: + p ESI Full ms2 260.00@cid40.00 [70.00-275.00]
259.73
236.60
248.13
218.80
232.07
270.27
123.00
113.07
147.07
161.33
178.60
203.67

(Continued)

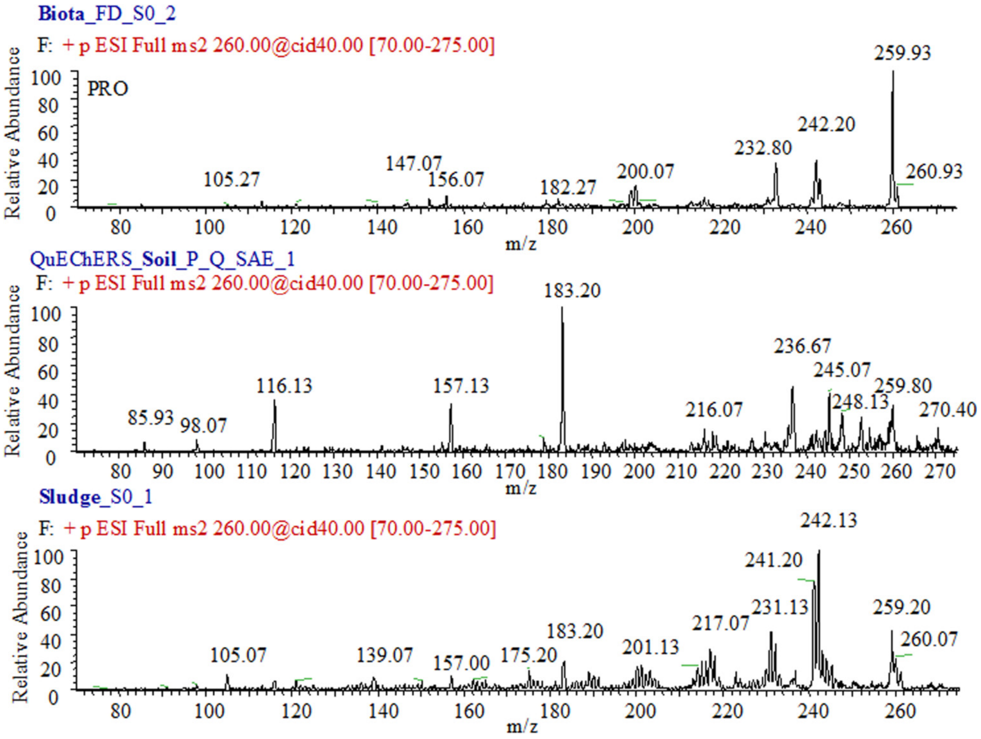

Figure S18. CID-MS/MS spectra of molecular ion $[M+H]^+$ of propranolol at m/z 260 in standard mixture separated *via* QuEChERS approach and in environmental effluent blank, freeze dried biota (FD), soil and standard sludge samples sample.

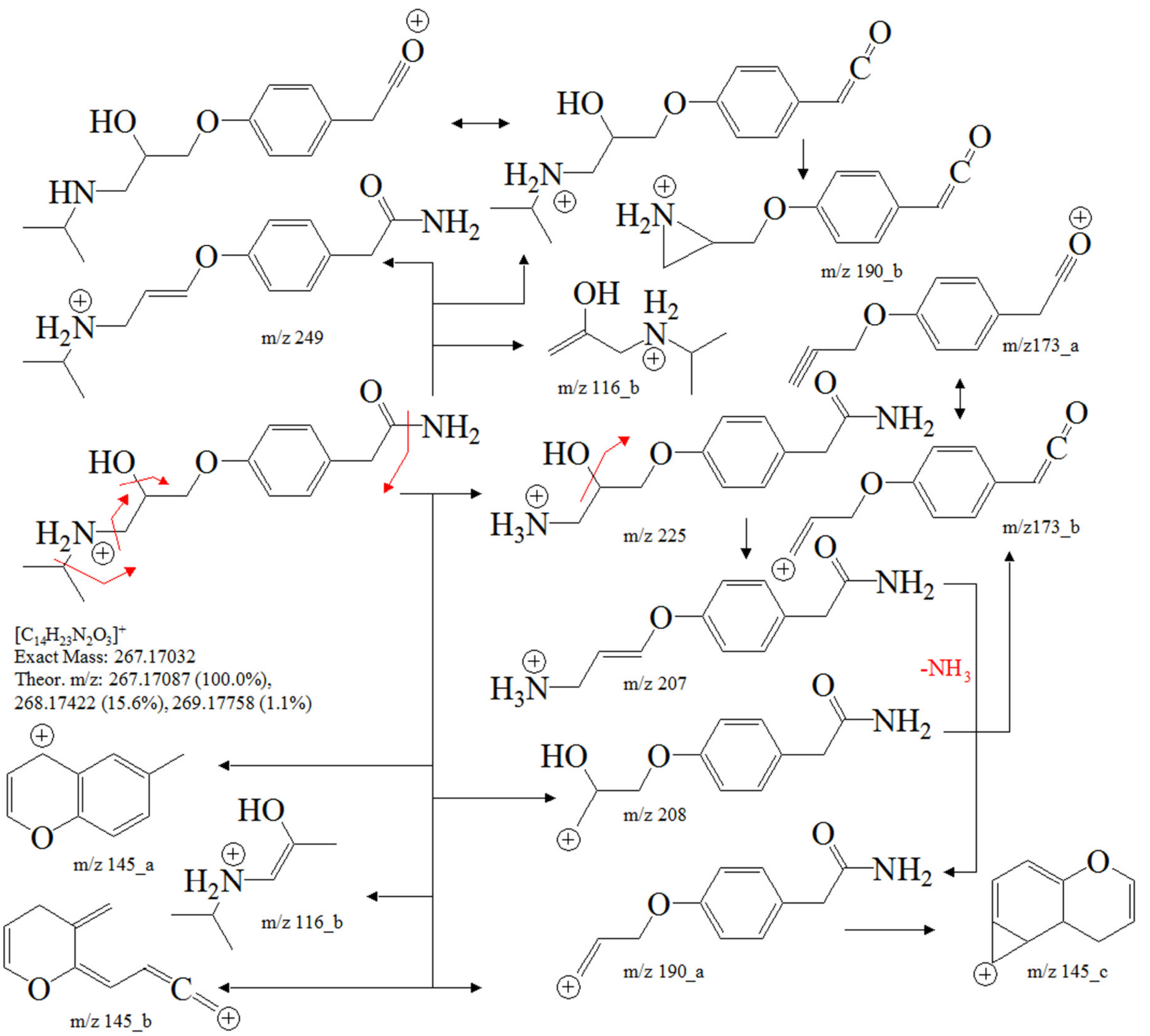

Figure S19. Fragmentation mass spectrometric reactions of atenolol according to [96–100].

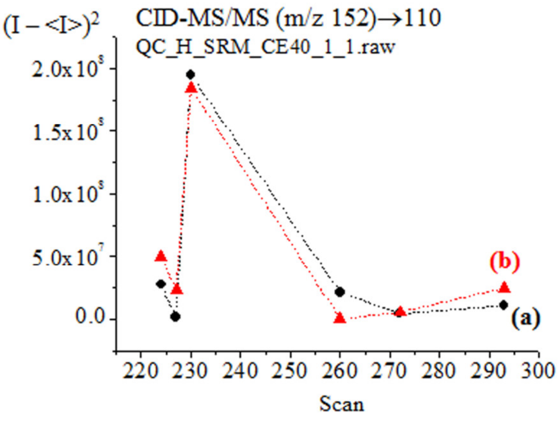

	Value	Error
x_c	340.59904	0.50994
ω	0.4453	0.00044
A^i	219695423.04113	28894348.848
r^2	0.9386	

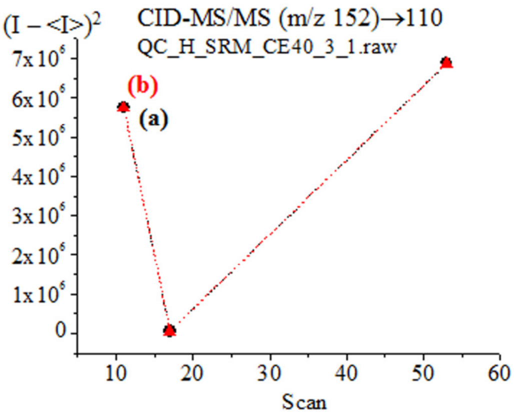

	Value	Error
x_c	57.87137	-
ω	2.15341	-
A^i	12793516.46748	-
r^2	**1**	

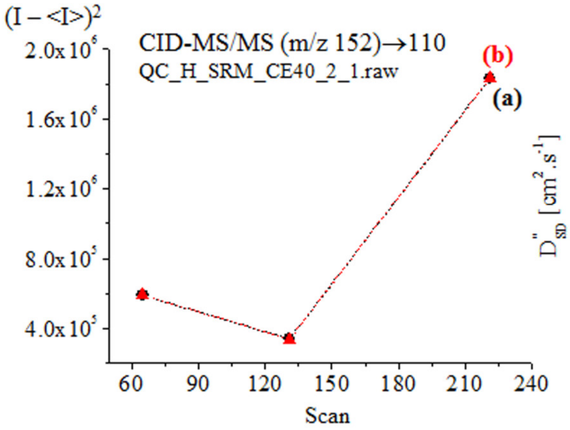

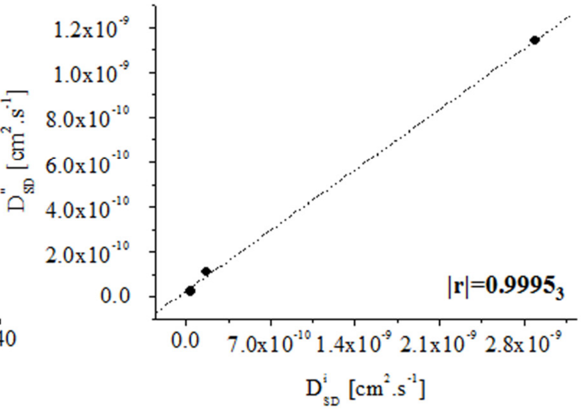

	Value	Error
x_c	255.79845	-
ω	5.17681	-
A^i	3117520.79637	-
r^2	**1**	

Figure S20. Functional relationships of experimental (a) and theoretical (b) $(I–<I>)^2=f(t)$ dependency of measurand intensity (I) with respect of scan time, shown as scan; theoretical fitting off approach uses SineSqr function; relationship between D'_{SD} and D''_{SD} parameters of equations (1) and (2); chemometrics.

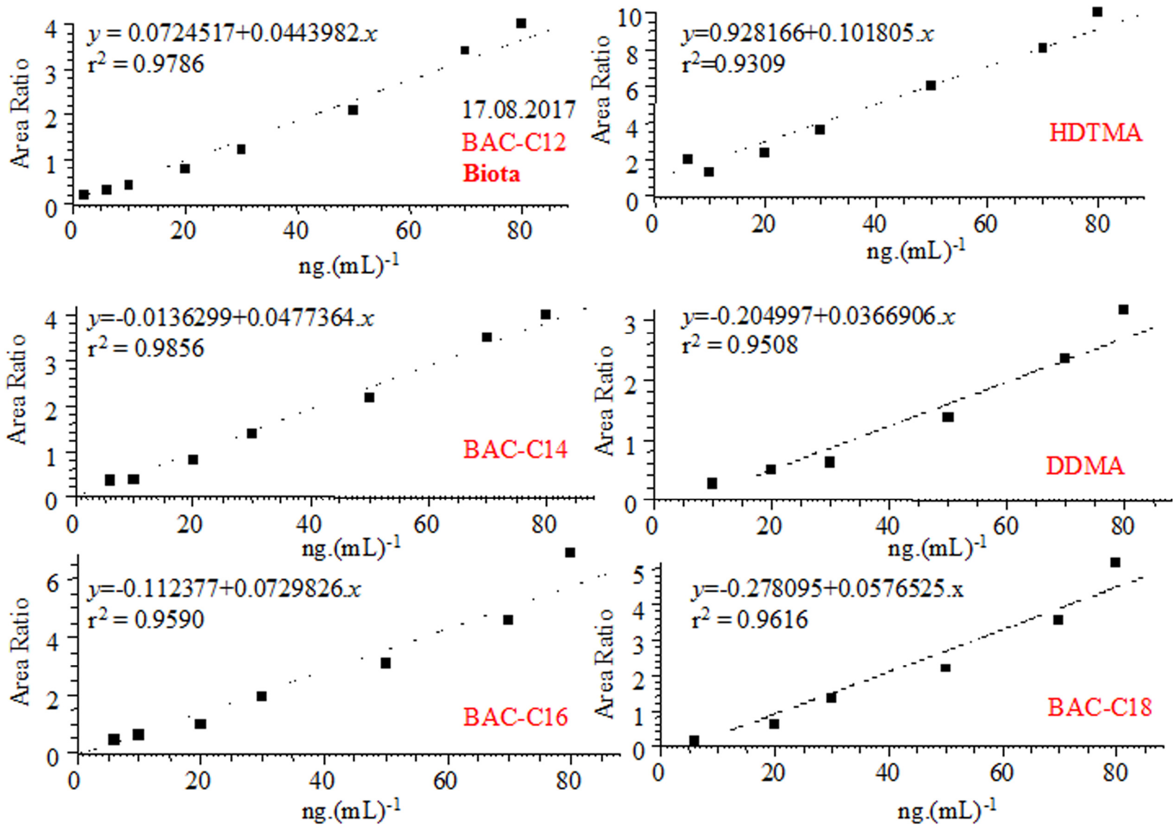

Figure S21. Standard linear calibration statistical equations and relationship mass spectrometric segment peak area of SRM spectra of biocides in biota using ICIS algorithm of peak detection and Savitzky-Golay smoothing function of the pattern of 3, in addition to, baseline correction and noise factors of 5 and 10; chemometrics.

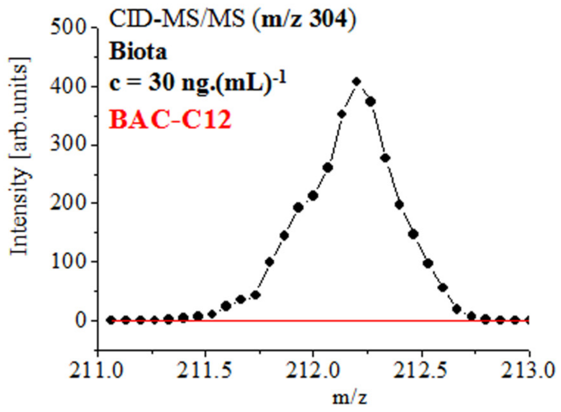

Area	Peak at	Width	Height
**197.60391**	212.2	0.4	407.78526

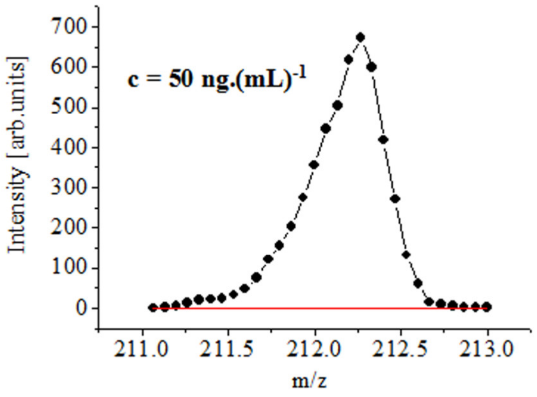

Area	Peak at	Width	Height
**340.36554**	212.26667	0.46667	671.8096

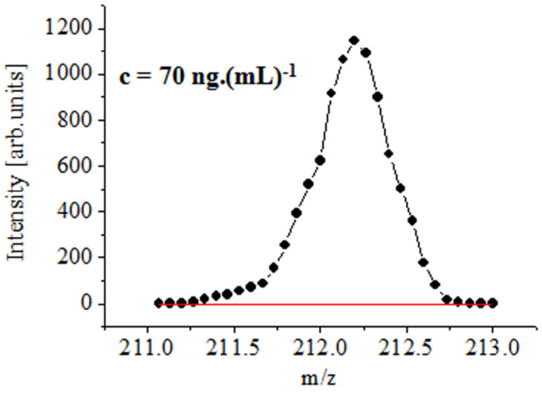

Area	Peak at	Width	Height
**610.82754**	212.2	0.46667	1146.39617

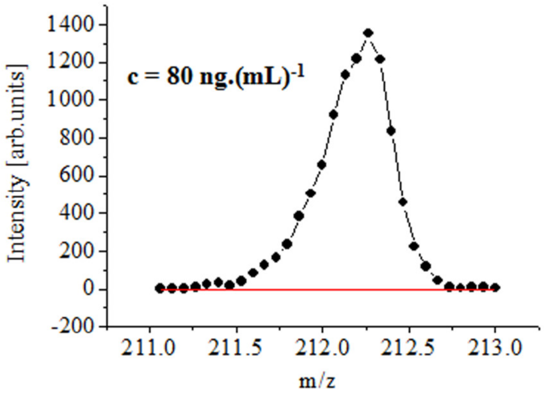

Area	Peak at	Width	Height
**653.96577**	212.26667	0.4	1351.5712

Figure S22. Experimental SRM spectra of BAC-C12 in biota, using the analyte molecular cation $[M]^+$ at m/z 304 at concentration levels c=30, 50, 70, and 80 ng.(mL)$^{-1}$; baseline correction of isotope pattern; data on trapezoidal integration approach.

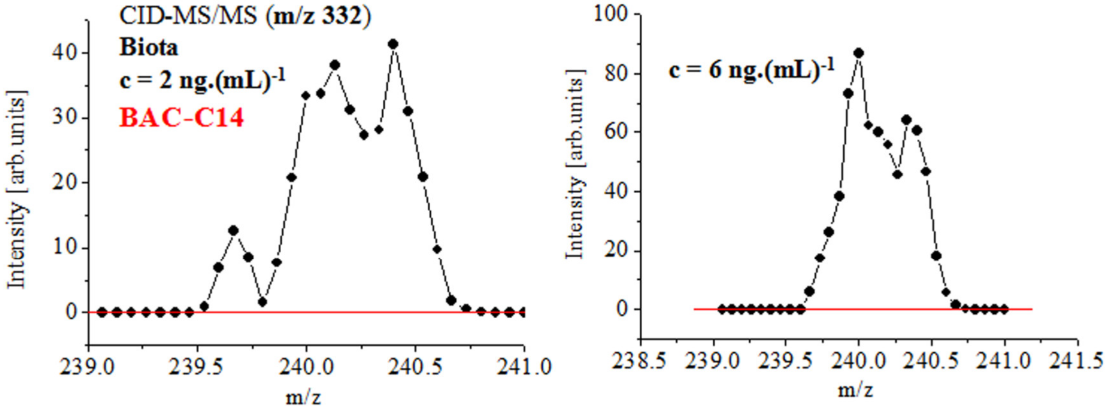

Area	Peak at	Width	Height
**23.71663**	240.4	0.66667	41.25522

Area	Peak at	Width	Height
**44.54311**	240	0.6	86.76046

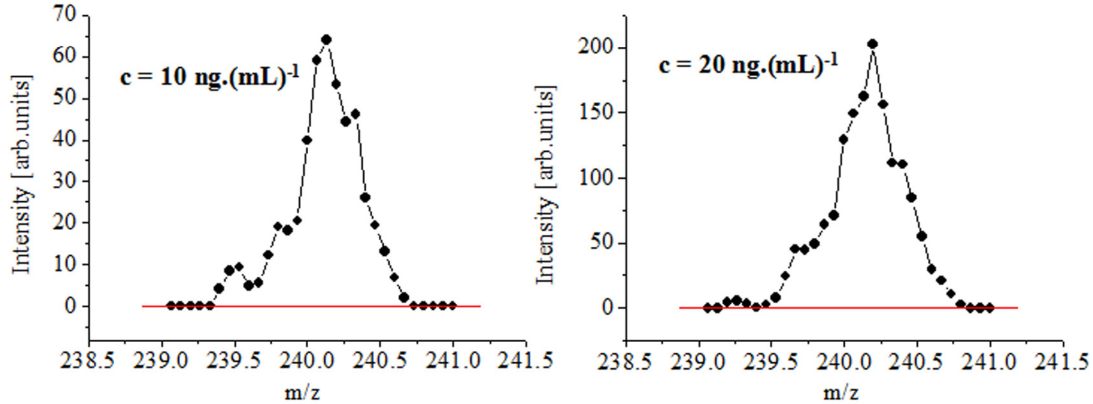

Area	Peak at	Width	Height
**31.75589**	240.13333	0.4	63.94186

Area	Peak at	Width	Height
**103.1025**	240.2	0.46667	203

Figure S23. Experimental SRM spectra of BAC-C14 in biota, using the analyte molecular cation $[M]^+$ at m/z 332 at concentration levels c=2, 6, 10, and 20 ng.(mL)$^{-1}$; baseline correction of isotope pattern; data on trapezoidal integration approach.

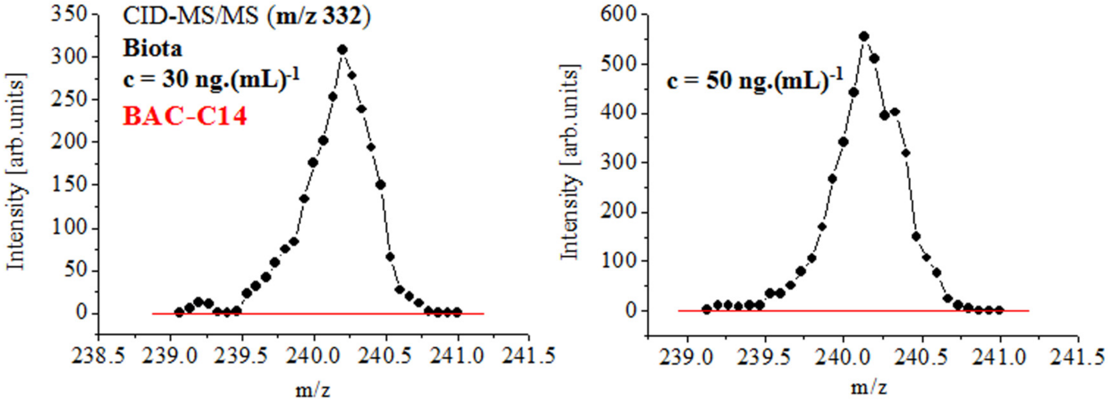

Area	Peak at	Width	Height
**160.52046**	240.2	0.46667	308.10304

Area	Peak at	Width	Height
**275.13693**	240.13333	0.46667	554.22196

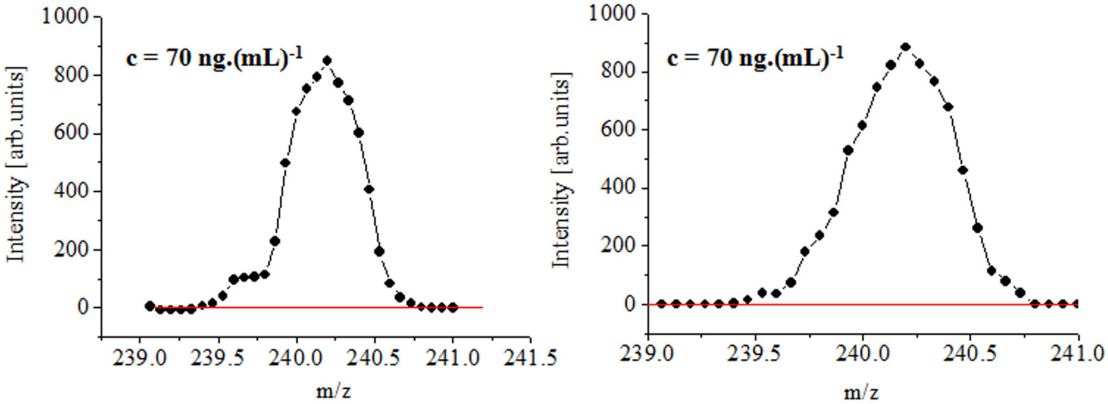

Area	Peak at	Width	Height
**471.57918**	240.2	0.53333	847.39767

Area	Peak at	Width	Height
**513.5366**	240.2	0.6	883.93925

Figure S24. Experimental SRM spectra of BAC-C14 in biota, using the analyte molecular cation $[M]^+$ at m/z 332 at concentration levels c=30, 50, 70, and 80 ng.$(mL)^{-1}$; baseline correction of isotope pattern; data on trapezoidal integration approach.

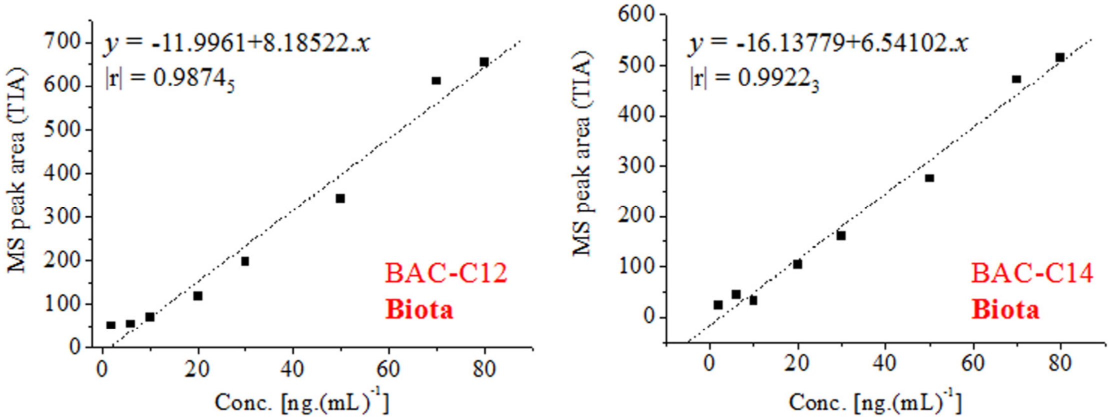

Figure S25. Functional relationship between mass spectrometric pattern area of SRM spectra of BAC-C12 and BAC-C14 in biota, obtained, due to trapezoidal integration of area under pattern *versus* analyte concentration [ng.(mL)$^{-1}$]; linear statistical equation; chemometrics.

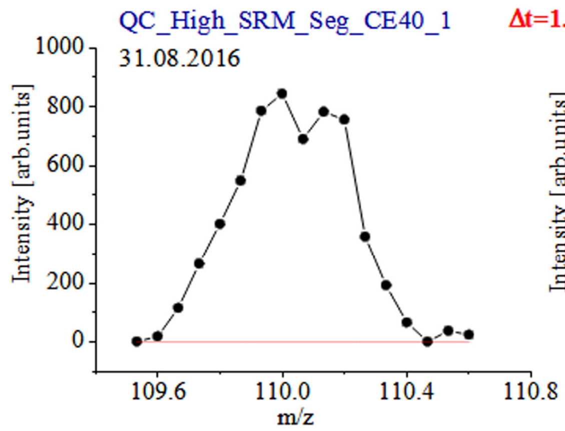

Area	Peak	Width	Height
**391.26902**	110	0.4	843.9452

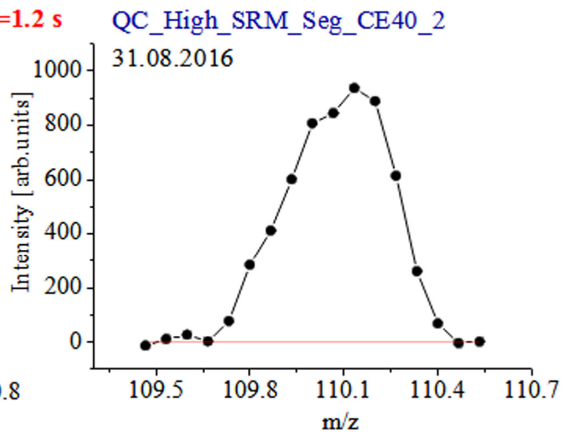

Area	Peak	Width	Height
**388.14882**	110.1	0.4	936.4659

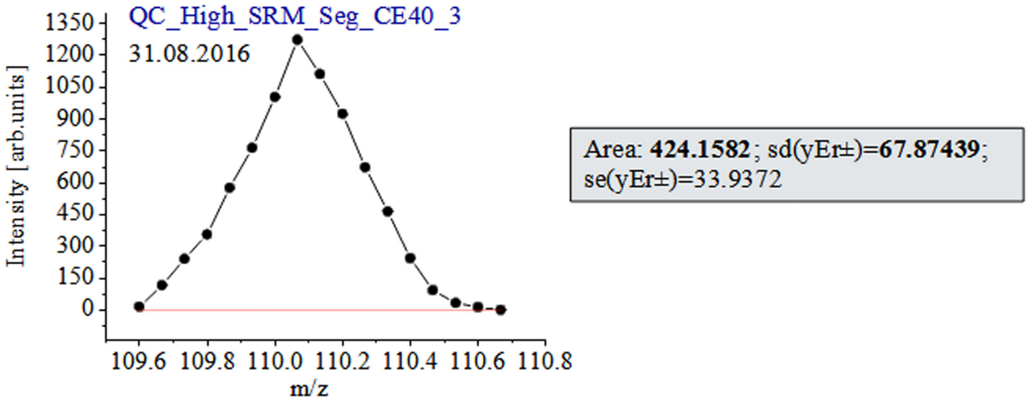

Area: **424.1582**; sd(yEr±)=**67.87439**; se(yEr±)=33.9372

Area	Peak	Width	Height
**525.94586**	110.1	0.4	1273.241

Figure S26. Data-processing of segment SRM mass spectra of paracetamol at m/z 152 measured (i=1–3) at Δt=1.2 s at CE = 40 V and using TIA; descriptive statistics (see data on **Table S2**.)

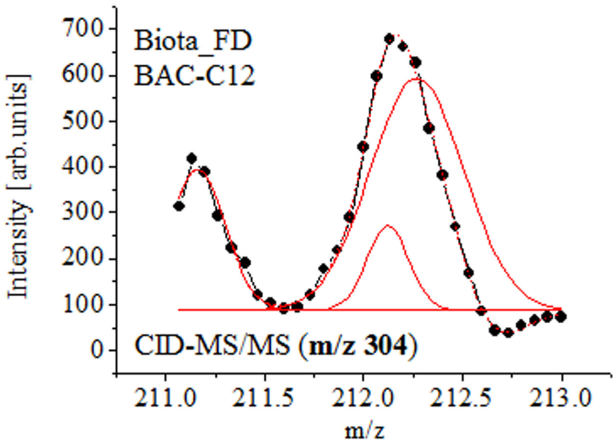

**r^2=0.99567**	
y_0	87.73204±21.64201
x_{c1}	211.16093±0.00892
w_1	0.27952±0.02832
A_1	**107.72856**±15.81885
x_{c2}	212.12191±0.02391
w_2	0.21056±0.09087
A_2	**48.54981**±78.49322
x_{c3}	212.27002±0.50607
w_3	0.47063±0.41118
A_3	**297.98061**±571.75988

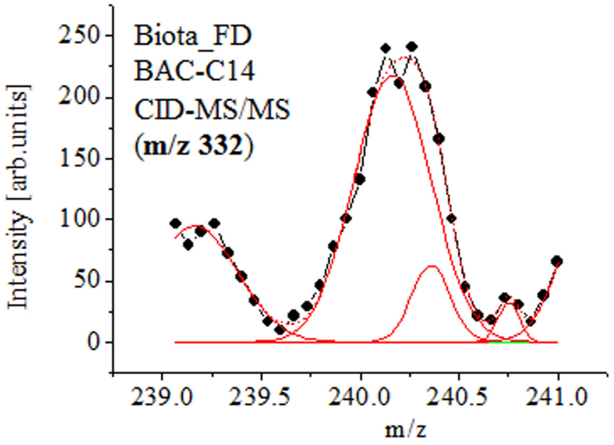

**r^2=0.98445**	
x_{c2}	239.16436±0.05018
w_2	0.43188±0.09426
A_2	**51.38167**±10.54482
x_{c3}	240.35876±0.03202
w_3	0.1947±0.14866
A_3	15.34245±31.48065
x_{c5}	240.16388±0.06692
w_5	0.38962±0.0608
A_5	106.75062±34.27113
x_{c6}	241.267±4.71917
w_6	0.35425±2.71093
A_6	**91.58519**±2791.35202
x_{c7}	240.75182±0.04405
w_7	0.10402±0.10979
A_7	4.31657±7.51906

(Continued)

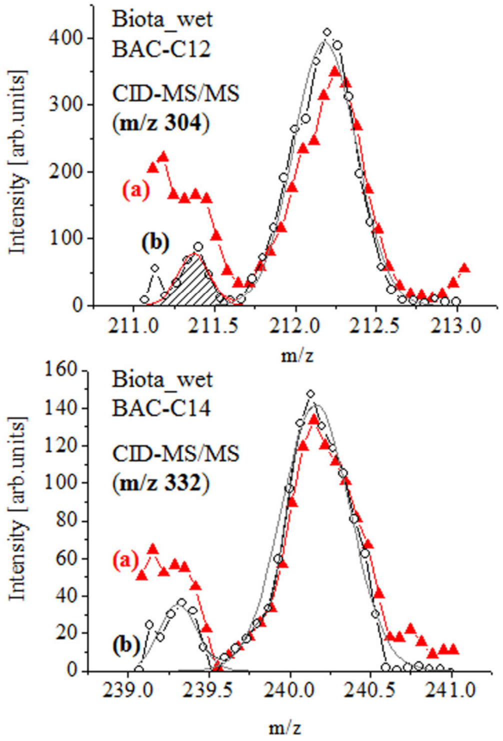

r^2=0.9802

y_0	0±0
x_{c2}	211.37034±0.02349
w_2	0.19525±0.047
A_2	19.111±3.98278
x_{c3}	212.18296±0.00653
w_3	0.38714±0.01307
A_3	191.93426±5.60741

r^2=0.98313

y_0	0±0
x_{c1}	239.30553±0.01868
w_1	0.23169±0.03824
A_1	10.40592±1.46759
x_{c3}	240.15978±0.01476
w_3	0.42723±0.02238
A_3	75.97119±4.4126

Figure S27. Experimental SRM spectra of BAC-C12 and BAC-C14 in freeze-dried and wet sample of biota, using the analyte molecular cation $[M]^+$ at m/z 304 and 332; without baseline correction of isotope pattern; data on curve fitting of the experimental pattern via Gaussian fitting function; chemometrics.

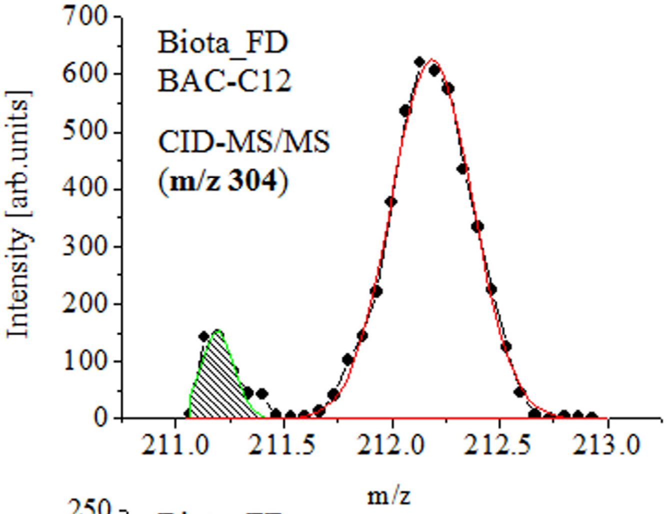

r^2=0.99274

y_0	0±0
x_{c1}	211.19145±0.01018
w_1	0.15384±0.02093
A_1	29.78212±3.44549
x_{c3}	212.18551±0.0039
w_3	0.3761±0.0078
A_3	**294.98671**±5.30109

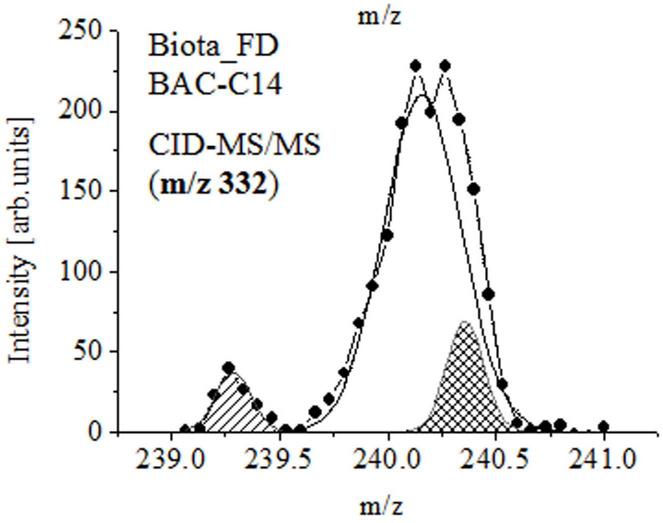

r^2=0.99274

y_0	0±0
x_{c1}	239.28547±0.01993
w_1	0.16606±0.03987
A_1	7.7348±1.60775
x_{c2}	240.35929±0.01433
w_2	0.17192±0.05525
A_2	15.00498±11.56921
x_{c3}	240.15924±0.02689
w_3	0.36231±0.0276
A_3	95.43686±12.60612

Figure S28. Experimental SRM spectra of BAC-C12 and BAC-C14 in freeze-dried sample of biota, using analyte molecular cation $[M]^+$ at m/z 304 and 332; with baseline correction of isotope pattern; data on curve fitting of the experimental pattern via Gaussian fitting function; chemometrics.

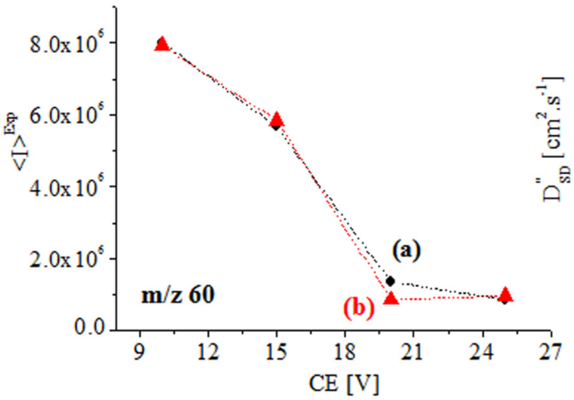

	Value	Error
X_c	26.55745	0.01639
ω	0.73727	0.00103
A^q_I	**8066000.8**	574835.8266
|r|	0.99177	

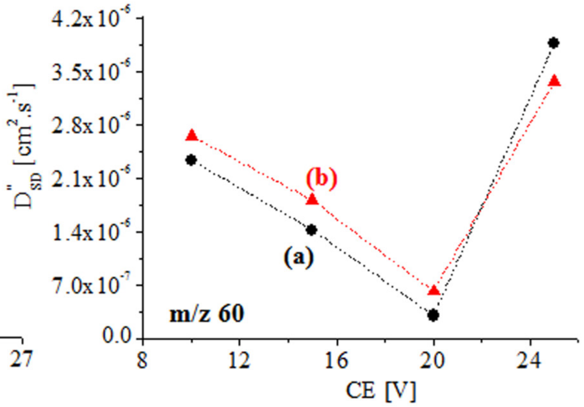

	Value	Error
X_c	25.3612	0.50708
ω	0.78144	0.03405
A^q_D	**$3.4228.10^{-6}$**	$1.5717.10^{-6}$
|r|	0.9138	

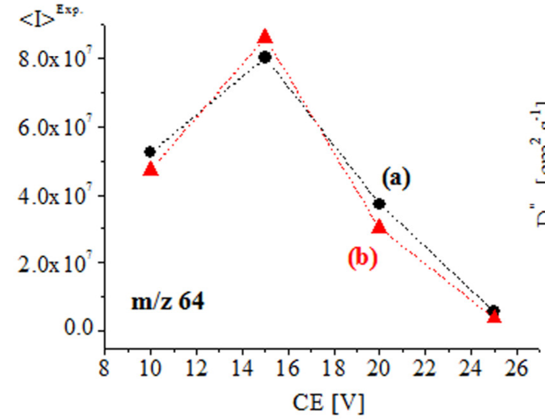

	Value	Error
X_c	7895443.89	7264.48
ω	0.00063	$4.4922.10^{-7}$
A^q_I	**86837035.7**	10263138.7
|r|	0.96026	

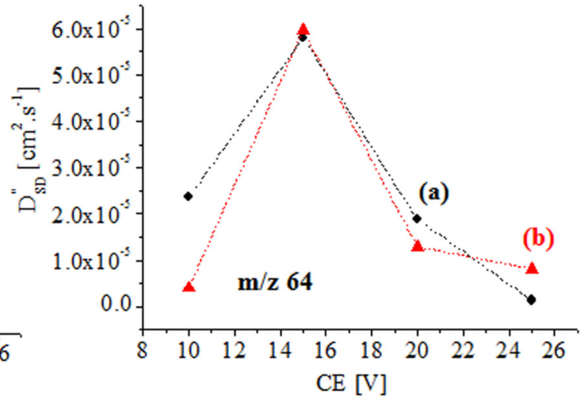

	Value	Error
X_c	25.39301	$2.8496.10^{-17}$
ω	5.10^{-7}	$6.641.10^{-26}$
A^q_D	**$1.9922.10^{12}$**	$1.6476.10^{12}$
|r|	0.72205	

Figure S29. Functional relationships between experimental ($<I>_{Exp.}$) intensity data on **Table S5** of fragmentation ions of paracetamol at m/z 60 and 64 with respect of collision energy CE [V] as well as functional relationships between experimental D''_{SD} [$cm^2.s^{-1}$] data on equation (2) of fragmentation ions of paracetamol at m/z 60 and 64 with respect of collision energy CE [V] (a); curve fitted SineSqr function of the above relationships (b); chemometrics.

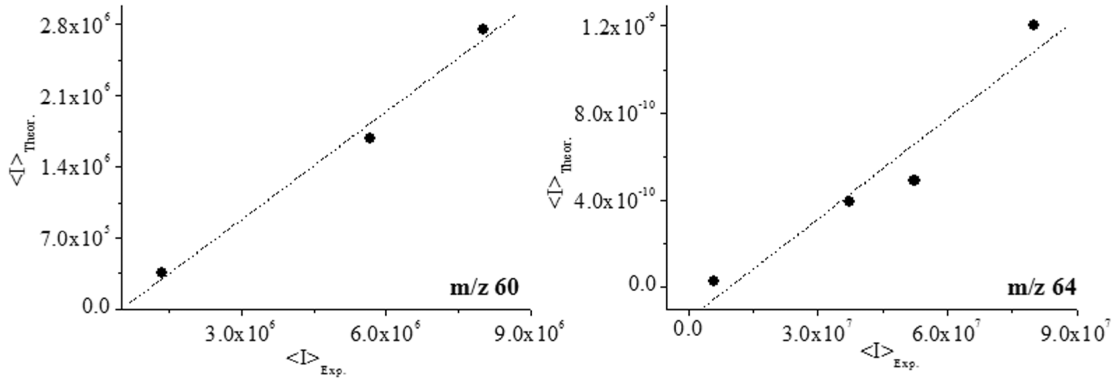

	Value	Error
A	-177259.04	217058.38
B	0.35352	0.03795
|r|	**0.99429**	
SD	181416.1	*p* (0.06807)
N	3	

	Value	Error
A	$-1.457.10^{-10}$	$1.5146.10^{-10}$
B	$1.533.10^{-17}$	$2.945.10^{-18}$
|r|	**0.96501**	
SD	$1.58.10^{-10}$	*p* (0.03499)
N	4	

Figure S30. Functional relationships between experimental ($<I>_{Exp.}$) and theoretical $<I>_{Theor.}$ intensity data on **Table S5** and equation (4) of fragmentation ions of paracetamol at m/z 60 and 64; chemometrics.

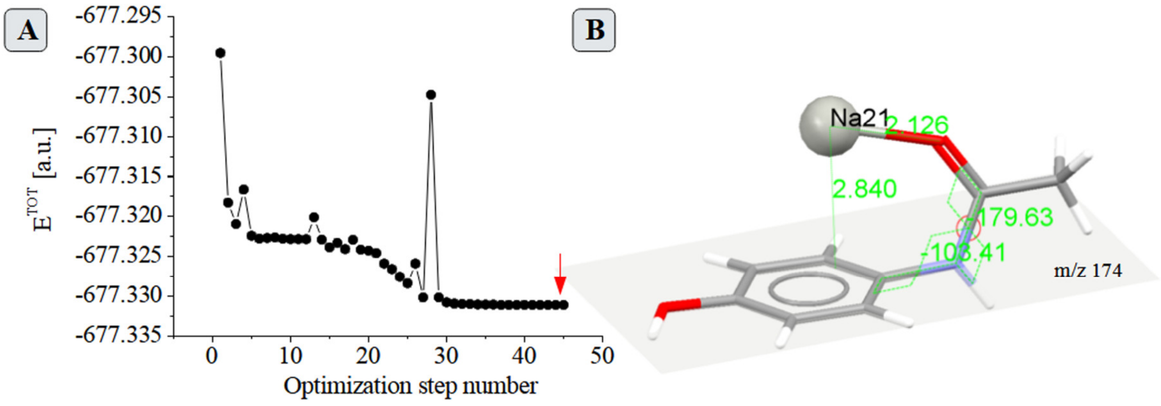
A
-677.295
-677.300
-677.305
-677.310
-677.315
-677.320
-677.325
-677.330
-677.335
E^TOT [a.u.]
0
10
20
30
40
50
Optimization step number
B
Na21
2.126
2.840
179.63
-103.41
m/z 174

(Continued)

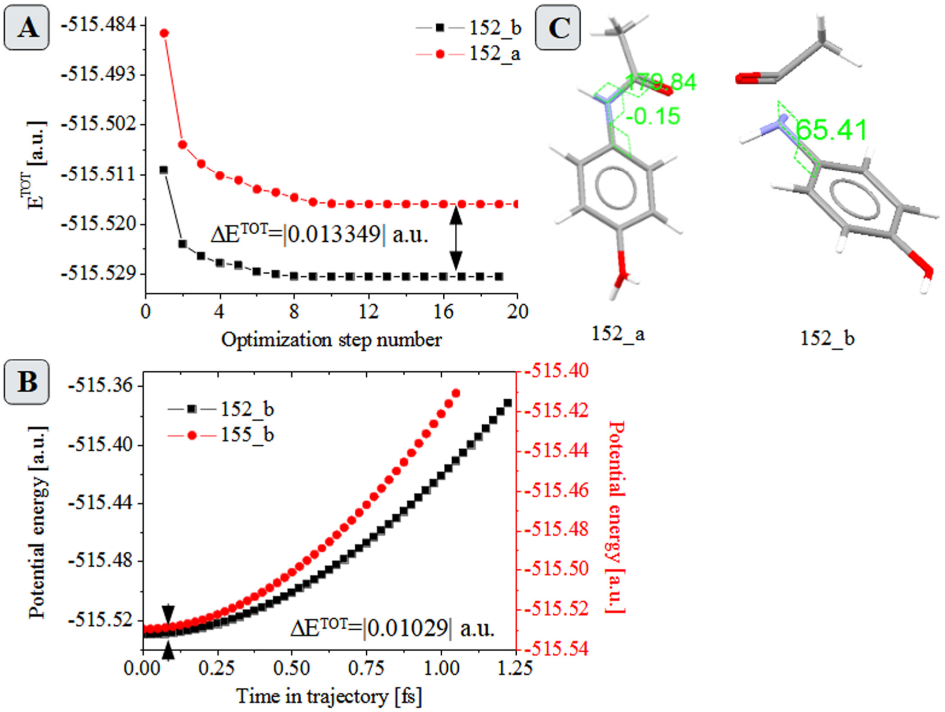

Figure S31. Theoretical M062X/SDD data on MS ion of paracetamol at m/z 174 ($[M+Na]^{+}$): Total energy, E^{TOT} [a.u.] with respect to optimization step number (A); most stable 3D molecular conformation of ion at m/z 174; selected geometry parameters such as torsion angle [°], coordinative $Na^{+}\cdots O=C$ bond [Å], and cation $Na^{+}\cdots$□ contacts [Å], respectively; Theoretical M062X/SDD data on MS ions of paracetamol and its d_3-derivative at m/z 152 and 155 ($[M+H]^{+}$ and $[d_3\text{-}M+H]^{+}$) depending on the proton accepting position of the cation: Total energy, E^{TOT} [a.u.] with respect to optimization step number and difference in energy data (ΔE) [a.u.] (A); molecular dynamics: Potential energy [a.u.] versus time in trajectory [fs], most stable 3D molecular conformation of ion at m/z 152 depending on the proton accepting position; selected geometry parameters such as torsion angle [°] (C), respectively; [a.u.] denotes *astronomical units* or Hartree.

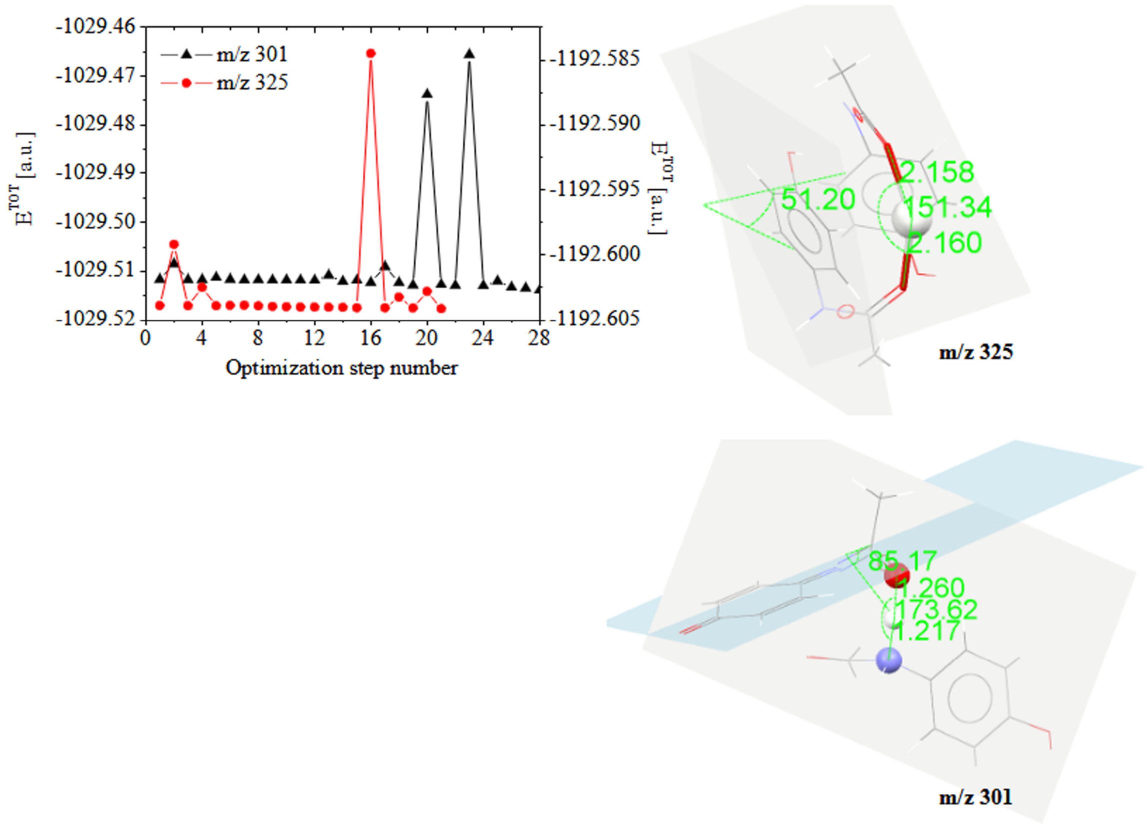

Figure S32. Theoretical M062X/SDD data on MS ions of paracetamol at m/z 310 ([2M+H]$^{+}$) and 325 ([2M+Na^{+}]): Total energy, E^{TOT} [a.u.] with respect to optimization step number; most stable 3D molecular conformations of ions; selected geometry parameters such as bond lengths [Å], angle [°], and interplanar angle [°] between the two phenyl-fragments of the ensembles of interacting noncovalent bonded dimers, respectively.

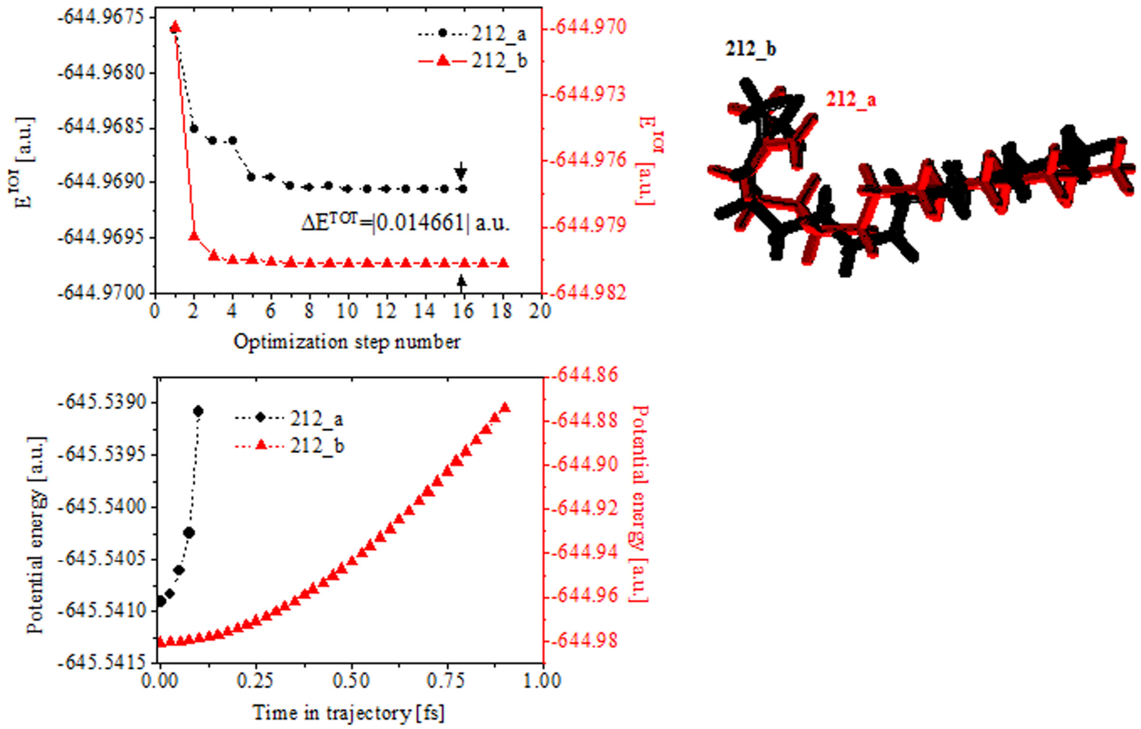

Figure S33. Relations between total energy E^{TOT} [a.u.] (MO62X/SDD) and optimization step number of fragmentation ions of BAC-C12 at m/z 212; difference in energy, ΔE^{TOT}; BOMD: Potential energy [a.u.] *versus* time in trajectory [fs] of species; most stable 3D molecular conformation of ions $212_{\_a}$ and $212_{\_b}$.

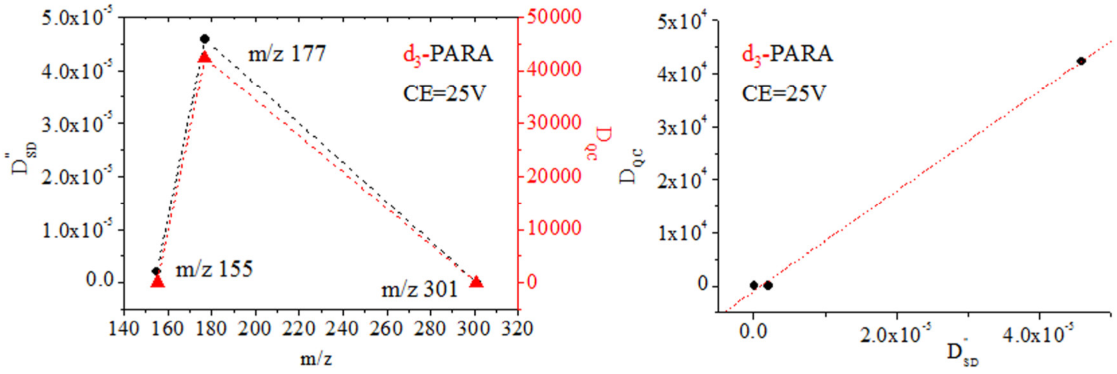

	Value	Error	
A	-952.60111	930.68732	
B	$9.430.10^8$	$3.510.10^7$	
|r|	sd(yEr±)	N	*p*
**0.9993_1**	1284.51	3	0.02368

Figure S34. Functional relationship between D''_{SD} [$cm^2.s^{-1}$] and D_{QC} data on equations (2) and (3) of fragmentation ions of CID-MS/MS reactions of molecular ion $[M+H]^+$ of d_3-paracetamol at m/z 155 and CE 25 V; chemometrics.

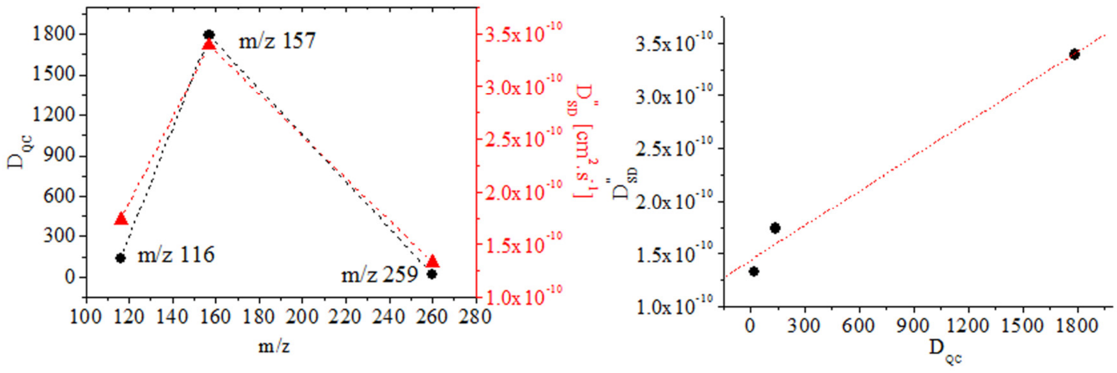

	Value	Error	
A	$1.441.10^{-10}$	$1.475.10^{-11}$	
B	$1.095.10^{-13}$	$1.42.10^{-14}$	
$\lvert r \rvert$	sd(yEr±)	N	*p*
0.99165	2.10^{-11}	3	0.36815

Figure S35. Functional relationship between $D"_{SD}$ [$cm^2.s^{-1}$] and D_{QC} data on equations (2) and (3) of fragmentation ions of CID-MS/MS reactions of molecular ion $[M+H]^+$ of propranolol at m/z 260 and CE 25 V; chemometrics.

Table S1
Experimental ESI(+) mass spectrometric conditions of measurements

Parameters/Samples	Biota		Effluent		Soil		Sludge	
	Wet	Freeze-dried	Blank	S0	SAE	SBE	Summer	Winter
Capillary temperature [ºC]	200	200	200	200	200	200	200	200
Source heater temperature [ºC]	0	0	0	0	0	0	0	0
Sheath gas flow [$\mu L.s^{-1}$]	60	60	60	60	60	60	60	60
Auxiliary gas flow [$\mu L.s^{-1}$]	0	0	0	0	0	0	0	0
Source voltage [kV]	4.5	4.5	4.5	4.5	4.5	4.5	4.5	4.5
Source current [uA]	80	80	80	80	80	80	80	80
Capillary voltage [V]	3	3	3	3	3	3	3	3
Tube lens offset [V]	10	10	10	10	10	10	10	10
MS run time [mins]	51	51	51	51	51	51	51	51
Segment 1 duration [mins]	15	15	15	15	15	15	15	15
Segment 1 number of scans	5	5	5	5	5	5	5	5
Injection volume [µL]	10	10	10	10	10	10	10	10

Table S2
Temporal distribution of m/z and intensity data on fragmentation peaks of ion at m/z 110 of CID-MS reaction of $[M+H]^+$ molecular cation ion of paracetamol at m/z 152; dataset labels are shown; parameters of equations (1) and (2); descriptive statistics

QC_H_SRM_CE40_3_i							QC_H_SRM_CE40_2_i					QC_H_SRM_CE40_1_i		
Scan	m/z	I [arb.units]	m/z	I [arb.units]	m/z	I [arb.units]	Scan	m/z	I [arb.units]	m/z	I [arb.units]	Scan	m/z	I [arb.units]
i	1		2		3		i	1		2		i	1	
11	110.232	3.08			–	–	65	110.07056	4627.5344	–	–	224	110.1011	2427.23181
17	110.16774	2175.34655			–	–	77	–	–	110.27009	1715.2538	227	110.11141	8815.36698
26	–	–	110.8851	1992.95056	–	–	89	–	–	110.24441	1430.18726	230	110.09836	21608.5939
44	–	–	–	–	110.03599	2214.2614	131	110.04513	4441.40842	–	–	260	110.10303	3066.17742
50	–	–	110.86594	2462.37318	–	–	206	–	–	110.29988	5166.57844	272	110.03129	5601.54976
53	110.16862	5025.16255	–	–	–	–	221	110.10341	2504.39711	–	–	293	110.07045	4398.60588
113	–	–	–	–	–	–	227	110.07345	26447.2084	–	–	–	–	–
131	–	–	110.88978	1475.07668	–	–	230	–	–	110.25419	13804.5775	–	–	–
134	–	–	–	–	110.08806	2850.57674	236	110.02922	5237.64839	–	–	–	–	–
191	–	–	–	–	110.06751	2191.91922	242	110.10271	4886.34305	–	–	–	–	–
206	–	–	110.7282	1463.09552	–	–	248	110.0301	1368.6029	–	–	–	–	–
215	–	–	–	–	110.07186	5317.65282	251	–	–	110.19998	4104.81461	–	–	–
218	–	–	–	–	110.16963	1907.27042	254	110.10929	8421.52251	–	–	–	–	–
221	–	–	–	–	110.05698	11262.9028	275	110.1037	3992.11545	–	–	–	–	–
224	–	–	–	–	110.07528	22529.2829	281	–	–	110.16456	3841.87384	–	–	–
227	110.28239	23056.1119	–	–	–	–	284	110.00073	3269.76492	–	–	–	–	–
230	110.15265	11872.695	–	–	–	–	299	110.03974	3297.93432	–	–	–	–	–
233	–	–	–	–	110.09372	4009.74695	317	110.03729	2146.13769	–	–	–	–	–
236	–	–	–	–	110.05123	3237.25633	–	–	–	–	–	–	–	–
239	–	–	–	–	110.00301	4172.19759	–	–	–	–	–	–	–	–
254	–	–	110.84117	1590.34933	110.13664	2901.57557	–	–	–	–	–	–	–	–
257	110.16633	6.02	–	–	–	–	–	–	–	–	–	–	–	–
260	110.15436	2918.16096	–	–	–	–	–	–	–	–	–	–	–	–
272	–	–	–	–	110.02872	1607.66866	–	–	–	–	–	–	–	–
287	110.15223	2329.4795	–	–	–	–	–	–	–	–	–	–	–	–
296	110.16923	6423.46631	–	–	–	–	–	–	–	–	–	–	–	–
305	–	–	–	–	110.00236	1848.17348	–	–	–	–	–	–	–	–
347	110.169	2662.5225	–	–	–	–	–	–	–	–	–	–	–	–
374	–	–	110.85917	3755.36237	–	–	–	–	–	–	–	–	–	–
395	–	–	–	–	110.03254	3796.37618	–	–	–	–	–	–	–	–
422	110.14404	5955.79933	–	–	–	–	–	–	–	–	–	–	–	–

425	110.22889	444.07003	–	–	–	–	–	–	–	–	–	–	–	–
Mean	110.1895		–	–	–	–	110.0730	–	–	–	–	–	110.0859	–
sd(yEr±)	0.03685		–	–	–	–	0.02922	–	–	–	–	–	0.03017	–
se(yEr±)	0.02127		–	–	–	–	0.01687	–	–	–	–	–	0.01232	–
<I>	–	2401.19637	–	–	–	–	–	3857.779	–	–	–	–	–	7652.92096
$<I>^2$	–	5765744.01	–	–	–	–	–	14882466.4	–	–	–	–	–	58567199.2
$<I^2>$	–	$9.9948.10^6$	–	–	–	–	–	$1.58041.10^7$	–	–	–	–	–	$1.01777.10^8$
$<I^2>–<I>^2$	–	4229055.99	–	–	–	–	–	921633.626	–	–	–	–	–	43209800.8
σ^2	–	$4.22906.10^6$	–	–	–	–	–	$1.58041.10^7$	–	–	–	–	–	$4.32095.10^7$
**lnP1**		**17.0532222**						**17.053206**	–	–	–	–	–	**17.053087**
A^i	12793516.46748		–	–	–	–	3117520.79637		–	–	–	–	219695423.0411	
D^i_{SD}	$1.6879749594.10^{-10}$		–	–	–	–	$4.113256939388.10^{-11}$		–	–	–	–	$2.898681639.10^{-9}$	
D''_{SD}	$1.11596329535.10^{-10}$		–	–	–	–	$2.432006812054.10^{-11}$		–	–	–	–	$1.14022.10^{-9}$	

Table S3
Temporal distribution of m/z and intensity data on fragmentation peaks of CID-MS/MS reaction of $[M+H]^{+}$ molecular ion of paracetamol at m/z 152 with respect to collision energy (CE, [V]); parameters of equation (2); descriptive statistics; experimental measurands and parameters of equation (2)

Scan	CE=10V											
	m/z	I [arb.units]	m/z	I [arb.units]	m/z	I [arb.units]	m/z	I [arb.units]	m/z	I [arb.units]	-	-
1	61.9584	244864.431	63.88515	$6.01.10^{7}$	93.94261	841184.047	325.43468	540197.593	174.23663	$2.20.10^{6}$	-	-
2	61.96603	324558.127	63.89074	$6.07.10^{7}$	93.90976	$1.09.10^{6}$	325.40492	721866.584	174.30328	845489.792	300.91813	164700.273
3	61.95123	384035.969	63.88537	$5.50.10^{7}$	93.96358	750549.789	325.41925	611790.599	174.28721	$1.02.10^{6}$	300.93079	114529.231
4	61.95052	171579.654	63.88946	$5.23.10^{7}$	93.94335	674096.501	325.45217	379660.249	174.26334	$1.41.10^{6}$	301.14857	145278.035
5	61.97158	334243.133	63.8885	$5.54.10^{7}$	93.89747	970387.31	325.4428	434636.401	174.20612	$2.66.10^{6}$	-	-
6	61.95433	292835.699	63.88573	$5.43.10^{7}$	-	-	325.41818	616007.928	174.25519	$1.70.10^{6}$	300.89628	143748.03
7	61.94459	381503.921	63.8891	$5.62.10^{7}$	93.92337	767732.078	325.41376	550761.564	174.2418	$1.63.10^{6}$	300.94895	326264.926
8	61.94893	247107.478	63.88829	$5.21.10^{7}$	93.86924	888320.053	325.45562	350329.184	174.27847	$1.29.10^{6}$	300.99401	177119.399
9	61.95139	336671.29	63.8896	$5.32.10^{7}$	93.83068	787019.51	325.43382	473054.913	174.24321	$1.54.10^{6}$	300.90586	160291.221
10	61.94894	397362.698	63.88867	$5.47.10^{7}$	93.76781	737963.352	325.50258	229805.735	174.24884	$1.64.10^{6}$	-	-
11	61.96369	418133.484	63.89172	$6.04.10^{7}$	93.85689	774978.834	-	-	174.24728	$1.50.10^{6}$	-	-
12	61.97606	461643.643	63.89293	$5.85.10^{7}$	-	-	-	-	174.24297	$1.66.10^{6}$	-	-
Mean	61.95714	-	63.88877	-	93.89048	-	325.43778	-	174.25453	-	300.96323	-
sd(yEr±)	0.01001	-	0.00244	-	0.06015	-	0.0282	-	0.02571	-	0.0879	-
se(yEr±)	0.00289	-	$7.038.10^{-4}$	-	0.01902	-	0.00892	-	0.00742	-	0.03322	-
<I>	-	332878.294	-	$5.6075.10^{7}$	-	828223.1474	-	490811.075	-	1.59129E6	-	175990.16
<I>^{2}	-	$1.108.10^{11}$	-	$3.1444.10^{15}$	-	$6.85953.10^{11}$	-	$2.40896.10^{11}$	-	$2.53220.10^{12}$	-	$3.0973.10^{10}$
<I^{2}>	-	$1.171.10^{11}$	-	$3.1533.10^{15}$	-	$6.99818.10^{11}$	-	$2.60237.10^{11}$	-	$2.74473.10^{12}$	-	$3.5075.10^{10}$
<I^{2}>–<I>^{2}	-	58563.0899	-	$8.934.10^{12}$	-	13864418110.84	-	$1.93414.10^{11}$	-	$2.125261.10^{11}$	-	$4.10256.10^{9}$
**D''_{SD}**	**$1.54536281652.10^{-12}$**		**$2.357602875.10^{-4}$**		**$3.6585426510878.10^{-7}$**		**$5.10383202690003.10^{-7}$**		**$5.6081396741.10^{-6}$**		**$1.08258353.10^{-7}$**	
Scan	CE=20V											
	m/z	I [arb.units]	m/z	I [arb.units]	m/z	I [arb.units]	m/z	I [arb.units]	m/z	I [arb.units]	m/z	I [arb.units]
1	60.88234	$3.29.10^{6}$	63.86275	$4.34.10^{7}$	82.57791	$1.81.10^{7}$	93.92434	733983.193	174.16328	$4.82.10^{6}$	324.95392	505373.39
2	60.89452	$3.09.10^{6}$	63.86984	$4.28.10^{7}$	82.98661	$2.08.10^{7}$	93.9527	826453.835	174.16471	$4.85.10^{6}$	324.97035	462884.919
3	60.89079	$2.97.10^{6}$	63.873	$4.13.10^{7}$	82.98151	$2.07.10^{7}$	93.50046	$1.07.10^{6}$	174.14686	$5.39.10^{6}$	324.96325	463598.201
4	60.88169	$3.15.10^{6}$	63.86781	$4.38.10^{7}$	82.99436	$2.02.10^{7}$	93.94365	548033.299	174.16379	$4.70.10^{6}$	324.96054	548246.603
5	60.90441	$2.65.10^{6}$	63.87133	$4.25.10^{7}$	82.55648	$1.70.10^{7}$	93.45748	899784.125	174.16154	$5.49.10^{6}$	324.97678	505580.766
6	60.93483	$3.16.10^{6}$	63.8738	$4.47.10^{7}$	$8.28.10^{1}$	$2.19.10^{7}$	93.45032	$1.06.10^{6}$	174.17078	$5.15.10^{6}$	324.98389	591084.09
7	60.91517	$3.09.10^{6}$	63.86964	$4.54.10^{7}$	$8.26.10^{1}$	$1.75.10^{7}$	93.95345	$5.24.10^{5}$	174.14719	$6.39.10^{6}$	-	-
8	60.88109	$3.34.10^{6}$	63.87134	$4.37.10^{7}$	$8.26.10^{1}$	$1.92.10^{7}$	93.45696	$8.28.10^{5}$	174.1582	$5.88.10^{6}$	324.99182	563383.979

9	60.91336	$3.30.10^{6}$	63.87423	$4.52.10^{7}$	$8.30.10^{1}$	$2.27.10^{7}$	93.47614	$8.53.10^{5}$	174.18424	$5.21.10^{6}$	324.97194	756590.356
10	60.92765	$2.88.10^{6}$	63.86695	$4.58.10^{7}$	$8.26.10^{1}$	$1.68.10^{7}$	93.45832	$7.71.10^{5}$	174.17512	$5.75.10^{6}$	324.99959	778854.851
11	60.90184	$2.93.10^{6}$	63.87227	$4.51.10^{7}$	$8.26.10^{1}$	$1.76.10^{7}$	93.47757	$8.67.10^{5}$	174.16972	$5.54.10^{6}$	325.00998	655337.894
12	60.93801	$3.18.10^{6}$	63.87357	$4.50.10^{7}$	$8.25.10^{1}$	$1.67.10^{7}$	93.47164	$7.85.10^{5}$	174.17434	$5.58.10^{6}$	324.98947	603352.281
Scan	CE=20V											
	m/z	I [arb.units]	m/z	I [arb.units]	m/z	I [arb.units]						
1	-	-	-	-	-	-	-	-	-	-	-	-
2	104.58864	737985.705	-	-	-	-	-	-	-	-	-	-
3	104.58245	$7.04.10^{5}$	108.78532	$4.39.10^{5}$	101.98257	349470.875	-	-	-	-	-	-
4	-	-	108.77784	$3.07.10^{5}$	-	-	-	-	-	-	-	-
5	-	-	108.86811	$3.70.10^{5}$	-	-	-	-	-	-	-	-
6	104.35004	$5.58.10^{5}$	108.76124	$3.40.10^{5}$	-	-	-	-	-	-	-	-
7	104.58298	$7.58.10^{5}$	108.76277	$4.38.10^{5}$	101.79551	421220.487	-	-	-	-	-	-
8	104.56023	$6.16.10^{5}$	$1.09.10^{2}$	$3.45.10^{5}$	101.79422	$5.42.10^{5}$	-	-	-	-	-	-
9	104.58636	$5.99.10^{5}$	$1.09.10^{2}$	$3.79.10^{5}$	-	-	-	-	-	-	-	-
10	-	-	-	-	-	-	-	-	-	-	-	-
11	104.59074	$6.42.10^{5}$	$1.09.10^{2}$	$3.72.10^{5}$	101.98202	$4.04.10^{5}$	-	-	-	-	-	-
12	104.62813	$5.50.10^{5}$	$1.09.10^{2}$	$2.85.10^{5}$	101.79505	$4.72.10^{5}$	-	-	-	-	-	-
Scan	CE=30V											
	m/z	I [arb.units]	m/z	I [arb.units]	m/z	I [arb.units]	m/z	I [arb.units]	m/z	I [arb.units]	m/z	I [arb.units]
1	60.90183	$1.17.10^{6}$	62.83791	$1.20.10^{6}$	63.87299	948161.067	101.7561	411971.474	104.68986	$1.02.10^{6}$	-	-
2	6.09E+01	979648.191	$6.29.10^{1}$	$1.06.10^{6}$	$6.39.10^{1}$	767327.168	101.76481	293515.635	$1.05.10^{2}$	996228.52	-	-
3	6.09E+01	944534.597	$6.28.10^{1}$	$1.08.10^{6}$	$6.39.10^{1}$	838428.986	$1.02.10^{2}$	302271.906	$1.05.10^{2}$	$1.15.10^{6}$	109.26626	$2.09.10^{6}$
4	6.09E+01	779820.104	$6.28.10^{1}$	977154.026	$6.39.10^{1}$	846333.772	$1.02.10^{2}$	403097.572	$1.05.10^{2}$	$1.08.10^{6}$	-	-
5	60.89271	993945.441	$6.29.10^{1}$	$1.13.10^{6}$	$6.39.10^{1}$	770515.525	101.78085	186387.035	$1.05.10^{2}$	$1.11.10^{6}$	109.1904	943762.13
6	60.90515	927562.267	62.82652	$1.17.10^{6}$	63.89364	844690.441	101.77339	301835.719	$1.05.10^{2}$	$1.18.10^{6}$	-	-
7	60.89874	985995.527	62.91471	$1.13.10^{6}$	63.89	724979.689	101.76695	259368.714	104.67241	$1.29.10^{6}$	109.21175	$1.34.10^{6}$
8	60.91304	826214.627	62.83128	$1.20.10^{6}$	63.88185	894717.015	101.78498	213121.317	104.66999	$1.08.10^{6}$	109.218	$1.27.10^{6}$
9	60.90677	845746.997	62.84141	$1.20.10^{6}$	63.87959	838762.945	101.75388	219639.561	104.80356	938407.862	-	-
10	60.90066	964030.634	62.8462	$1.09.10^{6}$	63.88953	850519.111	-	-	104.68381	937507.508	109.22656	$1.04.10^{6}$
11	60.89204	954369.156	62.83161	$1.35.10^{6}$	63.88017	798794.229	101.7686	323491.306	-	-	109.23798	$1.10.10^{6}$
12	60.89951	869742.806	62.8697	$1.17.10^{6}$	63.88028	751196.176	101.76925	290761.939	105.06114	780929.533	109.16026	$1.25.10^{6}$
Scan	CE=30V											
	m/z	I [arb.units]	m/z	I [arb.units]	m/z	I [arb.units]	m/z	I [arb.units]	m/z	I [arb.units]	m/z	I [arb.units]
1	104.68986	$1.02.10^{6}$	-	-	-	-	152.01889	$1.73.10^{-7}$	174.15948	$2.27.10^{6}$	324.97112	207771.373

2	104.68114	996228.52	-	-	-	-	152.00829	$1.75.10^{-7}$	173.76598	$9.44.10^{6}$	324.91349	247160.305
3	104.66529	$1.15.10^{6}$	109.26626	$2.09.10^{6}$	$1.52.10^{2}$	$2.02.10^{-7}$	-	-	174.17307	$4.90.10^{6}$	324.99059	181188.178
4	104.70864	$1.08.10^{6}$	-	-	$1.52.10^{2}$	$2.02.10^{-7}$	-	-	173.91001	$8.35.10^{6}$	324.97093	256754.72
5	104.68271	$1.11.10^{6}$	109.1904	943762.13	151.86428	$2.07.10^{-7}$	-	-	173.83389	$8.77.10^{6}$	325.06813	210802.092
6	104.67565	$1.18.10^{6}$	-	-	151.84267	$1.95.10^{-7}$	-	-	174.16826	$4.50.10^{6}$	324.99798	309125.189
7	104.67241	$1.29.10^{6}$	109.21175	$1.34.10^{6}$	-	-	152.01442	$1.68.10^{-7}$	173.82787	$8.74.10^{6}$	324.99478	288697.963
8	104.66999	$1.08.10^{6}$	109.218	$1.27.10^{6}$	151.86351	$2.09.10^{-7}$	-	-	174.17906	$4.47.10^{6}$	324.94669	294081.913
9	104.80356	938407.862	-	-	-	-	-	-	174.17086	$4.78.10^{6}$	324.96444	229394.575
10	104.68381	937507.508	109.22656	$1.04.10^{6}$	-	-	152.0049	$1.7.10^{-7}$	174.1645	$4.61.10^{6}$	325.00269	263438.208
11	-	-	109.23798	$1.10.10^{6}$	-	-	-	-	174.1868	$4.59.10^{6}$	324.97117	265576.537
12	105.06114	780929.533	109.16026	$1.25.10^{6}$	-	-	152.02517	$1.63.10^{-7}$	173.85678	$8.65.10^{6}$	324.9737	200993.773
Scan	CE=50V											
	m/z	I [arb.units]	m/z	I [arb.units]	m/z	I [arb.units]	m/z	I [arb.units]	m/z	I [arb.units]	m/z	I [arb.units]
1	62.79197	$2.11.10^{7}$	$6.48.10^{1}$	$1.14.10^{7}$	$9.27.10^{1}$	$5.75.10^{6}$	109.23999	$4.81.10^{6}$	151.70439	$2.67.10^{6}$	$1.74.10^{2}$	$1.31.10^{6}$
2	62.8057	$2.04.10^{7}$	64.79019	$1.21.10^{7}$	$9.27.10^{1}$	$5.74.10^{6}$	109.21345	$3.80.10^{6}$	152.09983	$1.64.10^{6}$	173.35045	604065.583
3	62.80868	$2.13.10^{7}$	64.80169	$1.11.10^{7}$	$9.23.10^{1}$	$1.29.10^{6}$	109.19976	$3.03.10^{6}$	151.86476	$2.44.10^{6}$	$1.73.10^{2}$	651157.61
4	62.79124	$2.39.10^{7}$	64.7892	$1.35.10^{7}$	$9.27.10^{1}$	$5.61.10^{6}$	109.23392	$3.54.10^{6}$	152.14558	$1.67.10^{6}$	$1.74.10^{2}$	$1.29.10^{6}$
5	62.79563	$1.97.10^{7}$	64.78346	$1.19.10^{7}$	$9.26.10^{1}$	$5.82.10^{6}$	109.2352	$4.25.10^{6}$	151.78621	$2.69.10^{6}$	$1.74.10^{2}$	$1.07.10^{6}$
6	62.81259	$1.94.10^{7}$	64.79864	$1.07.10^{7}$	$9.27.10^{1}$	$5.43.10^{6}$	109.22793	$3.88.10^{6}$	152.13246	$1.53.10^{6}$	$1.74.10^{2}$	$1.42.10^{6}$
7	62.81816	$1.96.10^{7}$	64.80423	$1.29.10^{7}$	$9.27.10^{1}$	$5.94.10^{6}$	109.24017	$3.60.10^{6}$	151.7493	$2.67.10^{6}$	$1.74.10^{2}$	$1.33.10^{6}$
8	62.78872	$2.31.10^{7}$	64.78627	$1.30.10^{7}$	$9.27.10^{1}$	$6.16.10^{6}$	109.22758	$3.83.10^{6}$	151.35181	$1.33.10^{6}$	$1.74.10^{2}$	$1.33.10^{6}$
9	62.78739	$2.22.10^{7}$	64.81049	$1.15.10^{7}$	$9.27.10^{1}$	$6.33.10^{6}$	109.23951	$4.40.10^{6}$	152.10079	$2.02.10^{6}$	173.68506	$1.53.10^{6}$
10	62.81722	$2.02.10^{7}$	64.80915	$1.18.10^{7}$	$9.27.10^{1}$	$6.22.10^{6}$	109.25781	$5.25.10^{6}$	152.05378	$1.96.10^{6}$	173.68124	$1.33.10^{6}$
11	62.812	$1.95.10^{7}$	64.81281	$1.12.10^{7}$	$9.27.10^{1}$	$6.04.10^{6}$	109.23248	$3.39.10^{6}$	152.05995	$1.79.10^{6}$	173.63735	$1.36.10^{6}$

Table S4
Temporal distribution of m/z and intensity data on fragmentation peaks of CID-MS/MS reaction of $[M+H]^+$ molecular cation of paracetamol at m/z 152 with respect to collision energy (CE, [V]), scan time[mins]; data on total ion current

Scan	CE=10V				
	m/z	I [arb.units]	Scan time	m/z	TIC
1	83.268	$6.84.10^7$	0.08503	82.64095	$2.70.10^9$
2	83.30901	$4.82.10^7$	0.17018	82.64095	$2.64.10^9$
3	83.31081	$4.57.10^7$	0.25533	82.64095	$2.59.10^9$
4	83.28823	$6.36.10^7$	0.34048	82.64095	$2.58.10^9$
5	83.28822	$6.16.10^7$	0.42563	82.64095	$2.62.10^9$
6	83.31201	$4.73.10^7$	0.51078	82.64095	$2.65.10^9$
7	83.30744	$5.28.10^7$	0.59593	82.64095	$2.60.10^9$
8	83.31192	$5.07.10^7$	0.68108	82.64095	$2.61.10^9$
9	83.28104	$6.73.10^7$	0.76623	82.64095	$2.59.10^9$
10	83.31252	$4.94.10^7$	0.85138	82.64095	$2.63.10^9$
11	83.29584	$5.61.10^7$	0.93653	82.64095	$2.56.10^9$
12	83.3053	$5.21.10^7$	1.02168	82.64095	$2.63.10^9$
Scan	CE=20V				
	Scan time	m/z	I [arb.units]	m/z	I [arb.units]
1	0.08502	$1.45.10^2$	$5.77.10^7$	-	-
2	0.17017	144.60149	$4.92.10^7$	-	-
3	0.25532	144.60149	$4.89.10^7$	-	-
4	0.34047	144.60149	$4.51.10^7$	-	-
5	0.42562	144.60149	$4.40.10^7$	-	-
6	0.51077	-	-	63.95256	$4.51.10^7$
7	0.59592	-	-	63.82711	$4.59.10^7$
8	0.68107	-	-	63.82711	$4.41.10^7$
9	0.76622	-	-	63.82711	$4.55.10^7$
10	0.85137	-	-	63.82711	$4.68.10^7$
11	0.93652	-	-	63.82711	$4.57.10^7$
12	1.02167	-	-	63.82711	$4.55.10^7$

Scan	CE=30V				
	Scan time	m/z	I [arb.units]	TIC	
1	0.08503	103.58705	$3.36.10^{7}$	$1.31.10^{9}$	-
2	0.17018	103.58705	$3.20.10^{7}$	$1.30.10^{9}$	-
3	0.25533	103.58705	$3.49.10^{7}$	$1.31.10^{9}$	-
4	0.34048	103.58705	$3.51.10^{7}$	$1.30.10^{9}$	-
5	0.42563	1.04E+02	$3.40.10^{7}$	$1.29.10^{9}$	-
6	0.51078	103.58705	$3.58.10^{7}$	$1.32.10^{9}$	-
7	$5.96.10^{-1}$	103.58705	$3.61.10^{7}$	$1.32.10^{9}$	-
8	0.68108	$1.04.10^{2}$	$3.54.10^{7}$	$1.31.10^{9}$	-
9	0.76623	$1.04.10^{2}$	$3.74.10^{7}$	$1.32.10^{9}$	-
10	0.85138	$1.04.10^{2}$	$3.51.10^{7}$	$1.30.10^{9}$	-
11	0.93653	$1.04.10^{2}$	$3.49.10^{7}$	$1.29.10^{9}$	-
12	1.02168	103.58705	$3.47.10^{7}$	$1.28.10^{9}$	-

Table S5

Temporal distribution of m/z and intensity data on fragmentation peaks of CID-MS/MS reaction of $[M+H]^+$ molecular cation of paracetamol and its d_3-deuterated derivative at m/z 152 and 155 with respect to collision energy (CE, [V]) and presence of formic acid, scan time[mins]; descriptive statistics; experimental measurands and parameters of equation (2)

Scan	CE=10V											
	m/z	I [arb.units]	m/z	I [arb.units]	-	-	m/z	I [arb.units]	m/z	I [arb.units]	-	-
1	60.24007	$8.28.10^{6}$	64.20138	$5.39.10^{7}$	-	-	101.09711	$1.20.10^{7}$	105.0842	$6.45.10^{7}$	-	-
2	60.24807	$8.04.10^{6}$	64.19495	$5.11.10^{7}$	-	-	101.09991	$1.17.10^{7}$	105.08666	$6.66.10^{7}$	-	-
3	60.2681	$8.34.10^{6}$	64.18907	$5.33.10^{7}$	-	-	101.11247	$1.11.10^{7}$	105.08621	$6.85.10^{7}$	-	-
4	60.26015	$7.92.10^{6}$	64.20196	$5.17.10^{7}$	-	-	101.09123	$1.12.10^{7}$	105.08517	$6.72.10^{7}$	-	-
5	60.29274	$8.26.10^{6}$	64.18803	$5.03.10^{7}$	-	-	101.11105	$1.10.10^{7}$	105.08692	$6.77.10^{7}$	-	-
6	60.26917	$7.42.10^{6}$	64.20201	$5.26.10^{7}$	-	-	101.11063	$1.13.10^{7}$	105.08759	$6.66.10^{7}$	-	-
7	60.27538	$7.76.10^{6}$	64.20265	$5.20.10^{7}$	-	-	101.10204	$1.15.10^{7}$	105.08809	$6.47.10^{7}$	-	-
8	60.26421	$7.84.10^{6}$	64.20229	$5.22.10^{7}$	-	-	101.10054	$1.11.10^{7}$	105.08847	$6.37.10^{7}$	-	-
9	60.25763	$8.33.10^{6}$	64.2026	$5.25.10^{7}$	-	-	101.13885	$1.18.10^{7}$	105.08819	$6.85.10^{7}$	-	-
10	60.26914	$8.40.10^{6}$	64.2027	$5.33.10^{7}$	-	-	101.11556	$1.12.10^{7}$	105.08845	$6.53.10^{7}$	-	-
11	60.25774	$7.68.10^{6}$	64.20206	$5.23.10^{7}$	-	-	101.10813	$1.11.10^{7}$	105.08956	$6.58.10^{7}$	-	-
12	60.27522	$7.93.10^{6}$	64.20244	$5.27.10^{7}$	-	-	101.11502	$1.13.10^{7}$	105.08716	$6.71.10^{7}$	-	-
Mean	60.2648	-	64.19934	-	-	-	101.10854	-	105.08722	-	-	-
sd(yEr±)	0.0137	-	0.00547	-	-	-	0.01224	-	0.00151	-	-	-
se(yEr±)	0.00395	-	0.00158	-	-	-	0.00353	-	$4.36.10^{-4}$	-	-	-
<I>	-	$8.01667.10^{6}$	-	$5.2325.10^{7}$	-	-	-	$1.13583.10^{7}$	-	$6.635.10^{7}$	-	-
<I>^{2}	-	$6.426699.10^{13}$	-	$2.7379.10^{15}$	-	-	-	$1.2901.10^{14}$	-	$4.4023.10^{15}$	-	-
<I^2>	-	$6.43555.10^{13}$	-	$2.7388.10^{15}$	-	-	-	$1.29106.10^{14}$	-	$4.40458.10^{15}$	-	-
<I^2>–<I>^{2}	-	$8.85021.10^{10}$	-	$8.9438.10^{11}$	-	-	-	$9.502.10^{10}$	-	$2.2575.10^{12}$	-	-
**D''_{SD}**	**$2.335393708.10^{-6}$**		**$2.36007675.10^{-5}$**				**$2.50741705068.10^{-6}$**		**$5.957091.10^{-5}$**		**-**	**-**
Scan	CE=15V											
	m/z	I [arb.units]	m/z	I [arb.units]	m/z	I [arb.units]	m/z	I [arb.units]	m/z	I [arb.units]	m/z	I [arb.units]
1	60.25215	$5.22.10^{6}$	64.20248	$7.92.10^{7}$	83.17375	$7.67.10^{7}$	101.10857	322948.952	105.08963	$1.02.10^{7}$	152.53276	$1.39.10^{6}$
2	60.28649	$5.72.10^{6}$	64.20357	$8.09.10^{7}$	83.1787	$7.78.10^{7}$	101.13086	221629.299	105.09422	$9.99.10^{6}$	152.56387	$1.37.10^{6}$
3	60.2986	$5.78.10^{6}$	64.20313	$8.03.10^{7}$	83.18995	$7.97.10^{7}$	101.12547	515882.163	105.0934	$1.02.10^{7}$	152.59102	$1.28.10^{6}$
4	60.30068	$5.50.10^{6}$	64.20373	$7.75.10^{7}$	83.1997	$7.57.10^{7}$	101.09865	245586.102	105.09496	$9.68.10^{6}$	152.58752	$1.06.10^{6}$
5	60.29861	$5.68.10^{6}$	64.20326	$8.38.10^{7}$	83.17856	$7.68.10^{7}$	101.1477	317719.792	105.09179	$1.02.10^{7}$	152.57409	$1.18.10^{6}$
6	60.28619	$5.71.10^{6}$	64.20179	$8.09.10^{7}$	83.19404	$7.50.10^{7}$	101.1281	262781.254	105.09196	$9.55.10^{6}$	152.57212	$1.30.10^{6}$
7	60.2974	$5.65.10^{6}$	64.20295	$8.10.10^{7}$	83.17981	$7.74.10^{7}$	101.09007	269005.469	105.09144	$9.85.10^{6}$	152.55833	$1.46.10^{6}$
8	60.27315	$5.33.10^{6}$	64.20306	$7.93.10^{7}$	83.18748	$7.75.10^{7}$	101.13192	407846.443	105.09109	$9.42.10^{6}$	152.57093	$1.23.10^{6}$
9	60.27369	$5.73.10^{6}$	64.20322	$7.88.10^{7}$	83.19313	$7.68.10^{7}$	101.14047	319573.334	105.09072	$9.94.10^{6}$	152.56109	$1.26.10^{6}$
10	60.28408	$5.84.10^{6}$	64.20347	$7.99.10^{7}$	83.19673	$7.69.10^{7}$	101.12079	295339.655	105.089	$9.97.10^{6}$	152.57464	$1.29.10^{6}$

11	60.29504	$6.17.10^{6}$	64.20317	$7.94.10^{7}$	83.19698	$7.91.10^{7}$	101.12797	271440.745	105.09396	$9.81.10^{6}$	152.57997	$1.22.10^{6}$
12	60.27927	$5.62.10^{6}$	64.20357	$8.04.10^{7}$	83.19995	$7.71.10^{7}$	101.12524	264807.902	105.09223	$9.90.10^{6}$	152.58946	$1.18.10^{6}$
Mean	60.28545	-	64.20312	-	83.18906	-	101.12298	-	105.092	-	152.57132	-
sd(yEr±)	0.01431	-	$5.345.10^{-4}$	-	0.00922	-	0.01654	-	0.00183	-	0.01621	-
se(yEr±)	0.00413	-	$1.54.10^{-4}$	-	0.00266	-	0.00477	-	$5.29.10^{-4}$	-	0.00468	-
<I>	-	$5.6625.10^{6}$	-	$8.0117.10^{7}$	-	$7.72455.10^{7}$	-	309546.76	-	$9.8925.10^{6}$	-	$1.26833.10^{6}$
<I>^{2}	-	$3.2064.10^{13}$	-	$6.41.10^{15}$	-	$5.9668.10^{15}$	-	$9.5819.10^{10}$	-	$9.7861.10^{13}$	-	$1.60866.10^{12}$
<I^2>	-	$3.2118.10^{13}$	-	$6.429.10^{15}$	-	$5.96849.10^{15}$	-	$1.0181.10^{11}$	-	$9.79195.10^{13}$	-	$1.6192.10^{12}$
<I^2>–<I>^{2}	-	$5.399.10^{10}$	-	$2.1944.10^{12}$	-	$1.6227.10^{13}$	-	$5.9908.10^{9}$	-	$5.7943.10^{10}$	-	10539011100
**D"$_{SD}$**	**$1.424787075.10^{-6}$**		**$5.790532873068.10^{-5}$**		**$4.2820592643.10^{-5}$**		**$1.580853329793923.10^{-7}$**		**$1.529019675.10^{-6}$**		**$2.781034249068.10^{-7}$**	

Scan	CE=20V													
	m/z	I [a.u.]	m/z	I [a.u.]	m/z	I [a.u.]	m/z	I [arb.units]	m/z	I [arb.units]	m/z	I [arb.units]	m/z	I [a.u.]
1	60.25922	$1.33.10^{6}$	64.20277	$3.88.10^{7}$	83.34733	$7.46.10^{6}$	101.1303	100409.742	105.0835	367329.628	152.5598	721308.647	174.55754	875586.3
2	60.26141	$1.47.10^{6}$	64.2033	$3.78.10^{7}$	-	-	101.1315	104684.093	-	-	152.5529	959850.959	174.13342	$1.39.10^{7}$
3	60.27737	$1.47.10^{6}$	64.20426	$3.76.10^{7}$	83.68252	189319.9	101.1395	69713.5032	105.0803	406554.591	152.5731	740901.608	174.14451	$1.44.10^{7}$
4	60.26071	$1.19.10^{6}$	64.20477	$3.74.10^{7}$	83.16145	$2.39.10^{7}$	101.1050	326534.425	-	-	152.5675	889292.758	174.13486	$1.29.10^{7}$
5	60.26646	$1.23.10^{6}$	64.20534	$3.74.10^{7}$	83.20106	$1.03.10^{7}$	-	-	105.0113	324374.285	152.5667	786952.262	174.13368	$1.34.10^{7}$
6	60.2625	$1.24.10^{6}$	64.20428	$3.65.10^{7}$	-	-	101.0821	67271.7282	-	-	152.5664	913923.416	174.14042	$1.44.10^{7}$
7	60.27353	$1.34.10^{6}$	64.20323	$3.70.10^{7}$	-	-	-	-	105.0622	340433.457	152.5619	916928.706	174.54452	$1.32.10^{6}$
8	60.27592	$1.35.10^{6}$	64.20471	$3.71.10^{7}$	83.34692	$8.15.10^{6}$	101.1266	115871.618	105.0428	295262.357	152.562	902168.823	174.55126	$1.28.10^{7}$
9	60.26292	$1.24.10^{6}$	64.20433	$3.87.10^{7}$	-	-	-	-	-	-	-	-	174.55892	966768.1
10	60.2587	$1.36.10^{6}$	64.20389	$3.60.10^{7}$	-	-	101.1419	154823.91	105.0282	295855.133	152.5638	792912.395	174.55259	$1.27.10^{7}$
11	60.27536	$1.50.10^{6}$	64.20466	$3.69.10^{7}$	83.89775	308542.1	-	-	105.0129	246942.542	152.5613	893087.993	174.55735	909272.3
12	60.28495	$1.49.10^{6}$	64.20614	$3.61.10^{7}$	-	-	101.1019	66758.8589	105.0853	296658.091	152.5650	815923.83	174.14362	$1.40.10^{7}$
Mean	60.26825	-	64.20431	-	83.4395	-	101.1199	-	105.0508	-	152.5636	-	174.34606	-
sd(yEr±)	0.00874	-	$9.36.10^{-4}$	-	0.28993	-	0.02115	-	0.03124	-	0.00514	-	0.21693	-
se(yEr±)	0.00252	-	$2.70.10^{-4}$	-	0.11836	-	0.00748	-	0.01104	-	0.00155	-	0.06262	-
<I>	-	$1.35.10^{6}$	-	$3.727.10^{7}$	-	$8.384.10^{6}$	-	125758.485	-	321676.26	-	848477.39	-	$9.381.10^{6}$
<I>^{2}	-	$1.83.10^{12}$	-	$1.389.10^{15}$	-	$7.030.10^{13}$	-	$1.5815.10^{10}$	-	$1.03.10^{11}$	-	$7.1991.10^{11}$	-	$8.8.10^{13}$
<I^2>	-	$1.83.10^{12}$	-	$1.390.10^{15}$	-	$1.333.10^{14}$	-	$2.2363.10^{10}$	-	$1.056.10^{11}$	-	$7.2569.10^{11}$	-	$1.232.10^{14}$
<I^2>–<I>^{2}	-	$1.13.10^{10}$	-	$7.143.10^{11}$	-	$6.294.10^{13}$	-	$6.54790.10^{9}$	-	$2.15338.10^{9}$	-	$5.7811.10^{9}$	-	$3.527.10^{13}$
**D"$_{SD}$**	**$2.986675933068.10^{-7}$**		**$1.88509275.10^{-5}$**		**$1.66109325291.10^{-3}$**		**$1.7278607764.10^{-7}$**		**$5.682348197.10^{-8}$**		**$1.5255172346.10^{-7}$**		**$9.30768140257.10^{-4}$**	

Scan	CE=25V											
	m/z	I [arb.units]	m/z	I [arb.units]	m/z	I [arb.units]	m/z	I [arb.units]	m/z	I [arb.units]	m/z	I [arb.units]
1	*61.25286*	*$2.06.10^{6}$*	64.21214	$6.03.10^{6}$	83.27908	$1.03.10^{6}$	104.42914	429964.263	130.03878	$2.60.10^{6}$	152.12444	$1.14.10^{7}$
2	60.2848	$7.44.10^{5}$	64.21101	$5.81.10^{6}$	83.38596	$5.04.10^{5}$	104.33474	861285.131	130.03209	$2.58.10^{6}$	152.12493	$1.15.10^{7}$
3	60.28286	660633.362	64.20687	$5.74.10^{6}$	83.29121	782135.112	104.32026	$1.15.10^{6}$	130.04682	$2.71.10^{6}$	152.12546	$1.14.10^{7}$

4	60.28592	776410.484	64.21211	5.65.10^{6}	83.24556	1.28.10^{6}	104.31591	1.23.10^{6}	130.04594	2.74.10^{6}	152.12512	1.16.10^{7}
5	60.27489	798836.238	64.20465	5.74.10^{6}	-	-	104.46954	2.59.10^{6}	130.04624	2.98.10^{6}	152.12371	1.16.10^{7}
6	60.27541	765231.717	64.21117	5.85.10^{6}	-	-	104.38247	861518.51	130.04895	2.60.10^{6}	152.12601	1.15.10^{7}
7	60.27613	715387.69	64.2118	6.31.10^{6}	83.18815	833717.535	104.39921	533517.328	130.05007	2.73.10^{6}	152.12691	1.07.10^{7}
8	60.28133	798430.903	64.20672	5.62.10^{6}	83.26798	883803.047	104.44177	364764.336	130.0473	2.60.10^{6}	152.12393	1.13.10^{7}
9	60.26499	748927.134	64.2079	5.79.10^{6}	-	-	104.43435	355815.313	130.03942	2.99.10^{6}	152.12575	1.08.10^{7}
10	60.27787	676325.298	64.20925	5.46.10^{6}	83.29044	944370.082	104.40072	651212.645	130.03901	2.92.10^{6}	152.12891	1.11.10^{7}
11	60.27478	694207.859	64.20935	5.96.10^{6}	-	-	104.43402	330268.478	130.03451	2.41.10^{6}	152.12512	1.13.10^{7}
Mean	60.36653	-	64.20936	-	83.27834	-	-	-	-	-	-	-
sd(yEr±)	0.29402	-	0.00254	-	0.05946	-	-	-	-	-	-	-
se(yEr±)	0.08865	-	7.6717.10^{-4}	-	0.02247	-	-	-	-	-	-	-
<I>	-	858035.52	-	5.8145.10^{6}	-	829670.97	-	-	-	-	-	-
<I>^{2}	-	7.3622.10^{11}	-	3.3809.10^{13}	-	6.8835.10^{11}	-	-	-	-	-	-
<I^{2}>	-	8.8269.10^{11}	-	3.3855.10^{13}	-	7.157.10^{11}	-	-	-	-	-	-
<I^{2}>–<I>^{2}	-	1.464.10^{11}	-	4.6908.10^{10}	-	2.7426.10^{11}	-	-	-	-	-	-
**D"$_{SD}$**	**3.864972564888.10^{-6}**		**1.2378161544.10^{-6}**		**7.2371976061.10^{-7}**		**-**		**-**		**-**	
Scan	CE=25V											
	m/z	I [a.u.]	m/z	I [arb.units]	m/z	I [arb.units]	m/z	I [arb.units]	m/z	I [arb.units]	-	-
1	158.07402	3.11.10^{6}	174.12679	1.17.10^{7}	301.30931	537085.080	325.28771	532321.5881	338.52918	836932.862	-	-
2	158.06477	3.37.10^{6}	174.14468	1.12.10^{7}	301.32896	494102.334	325.28912	572290.6133	338.54564	726746.34	-	-
3	158.08793	2.97.10^{6}	174.53852	1.22.10^{6}	301.32113	549167.52	325.29998	650710.9803	338.54763	770789.431	-	-
4	158.07127	3.21.10^{6}	174.55183	873797.136	301.30264	660528.781	325.32712	543235.5189	338.51995	748069.922	-	-
5	158.08977	3.18.10^{6}	174.13872	1.19.10^{7}	301.31941	614428.528	325.33198	608209.3719	338.52388	755630.277	-	-
6	158.08846	3.10.10^{6}	174.13595	1.14.10^{7}	301.30761	484709.748	325.29294	550455.6931	338.53027	807564.612	-	-
7	158.06946	3.14.10^{6}	174.13437	1.21.10^{7}	301.3144	561240.059	325.31418	696386.8333	338.53933	946868.84	-	-
8	158.09151	3.02.10^{6}	174.12261	1.20.10^{7}	301.31065	696035.423	325.32201	592519.9352	338.54041	809899.588	-	-
9	158.09031	3.19.10^{6}	174.13029	1.12.10^{7}	301.32492	522525.660	325.30868	510647.9098	338.53179	825564.432	-	-
10	158.07875	2.97.10^{6}	174.12484	1.24.10^{7}	301.31301	673166.197	325.30645	640109.2628	338.52702	821823.649	-	-
11	158.07702	2.74.10^{6}	174.11664	1.20.10^{7}	301.31336	526219.946	325.29283	506896.5061	338.52684	716678.351	-	-
Mean	158.0803	-	174.20593	-	301.31504	-	325.30664	-	-	-	-	-
sd(yEr±)	0.00966	-	0.16794	-	0.00785	-	0.01564	-	-	-	-	-
se(yEr±)	0.00291	-	0.05063	-	0.00237	-	0.00472	-	-	-	-	-
<I>	-	3.0909.10^{6}	-	9.8176.10^{6}	-	574473.571	-	582162.20116	-	-	-	-
<I>^{2}	-	9.5537.10^{12}	-	9.6386.10^{13}	-	3.3002.10^{11}	-	3.389128.10^{11}	-	-	-	-
<I^{2}>	-	9.578.10^{12}	-	1.1362.10^{14}	-	3.3504.10^{11}	-	3.42347.10^{11}	-	-	-	-
<I^{2}>–<I>^{2}	-	2.4545.10^{10}	-	1.7229.10^{13}	-	5.0251.10^{9}	-	3434171540.5			-	-
**D"$_{SD}$**	**6.4770327369.10^{-7}**		**4.5464775888941.10^{-4}**		**1.32602780522.10^{-7}**		**9.0620918611867.10^{-8}**					

Scan	CE=25V (d_3-PARA)											
	m/z	I [a.u.]	m/z	I [a.u.]	m/z	I [a.u.]	-	-	-	-	-	-
1	154.88334	912173.864	176.63932	5.76.10^{6}	300.70569	149712.762	-	-	-	-	-	-
2	154.98327	870925.844	176.67674	5.43.10^{6}	300.71814	187148.536	-	-	-	-	-	-
3	154.98356	705870.815	176.60034	4.11.10^{6}	300.71405	127602.552	-	-	-	-	-	-
4	154.73815	1.09E+06	176.69049	6.07.10^{6}	300.76054	126733.212	-	-	-	-	-	-
5	155.05292	837492.239	176.65813	4.96.10^{6}	300.75186	144082.184	-	-	-	-	-	-
6	154.81593	1.39.10^{6}	176.6353	7.07.10^{6}	-	-	-	-	-	-	-	-
7	154.91777	1.51.10^{6}	176.62217	8.47.10^{6}	-	-	-	-	-	-	-	-
8	155.03868	696405.382	176.70358	6.02.10^{6}	300.75979	307318.275	-	-	-	-	-	-
9	154.66856	867511.353	176.65664	6.43.10^{6}	300.76046	151661.651	-	-	-	-	-	-
10	154.63065	1.48.10^{6}	176.68874	6.80.10^{6}	300.77551	117596.15	-	-	-	-	-	-
11	154.79112	868582.98	176.84807	3.51.10^{6}	300.74011	92371.8528	-	-	-	-	-	-
12	154.61889	814673.102	-	-	300.78891	127896.459	-	-	-	-	-	-
Mean	154.84357	-	176.6745	-	300.74751	-	-	-	-	-	-	-
sd(yEr±)	0.15643	-	0.0656	-	0.02747	-	-	-	-	-	-	-
se(yEr±)	0.04516	-	0.01978	-	0.00869	-	-	-	-	-	-	-
<I>	-	1.00382.10^{6}	-	5.8754.10^{6}	-	153212.3633	-	-	-	-	-	-
<I>^{2}	-	1.007654.10^{12}	-	3.4521.10^{13}	-	2.34743.10^{10}	-	-	-	-	-	-
<I^2>	-	1.0871.10^{12}	-	3.6259.10^{13}	-	2.66707.10^{10}	-	-	-	-	-	-
<I^2>–<I>^{2}	-	7.95154.10^{10}	-	1.7385.10^{12}	-	3.19667.10^{9}	-	-	-	-	-	-
**D"$_{SD}$**	**2.0982525757.10^{-6}**		**4.587658886515.10^{-5}**		**8.4353773745635677.10^{-8}**		-		-		-	

Table S6

Temporal distribution of m/z and intensity data on fragmentation peaks of CID-MS/MS reaction of $[M+H]^+$ molecular cation of paracetamol at m/z 152 depending on presence of formic acid at collision energy CE=10 V, scan time [mins]; data on total ion current; descriptive statistics

Scan	CE=10V			
	Scan time [mins]	m/z	I [arb.units]	TIC
1	$8.50.10^{-2}$	$6.42.10^{1}$	$7.94.10^{7}$	$1.04.10^{9}$
2	$1.70.10^{-1}$	$6.42.10^{1}$	$8.39.10^{7}$	$1.06.10^{9}$
3	$2.55.10^{-1}$	$6.42.10^{1}$	$8.13.10^{7}$	$1.05.10^{9}$
4	$3.40.10^{-1}$	$6.42.10^{1}$	$8.12.10^{7}$	$1.05.10^{9}$
5	$4.26.10^{-1}$	$6.42.10^{1}$	$7.94.10^{7}$	$1.06.10^{9}$
6	$5.11.10^{-1}$	$6.42.10^{1}$	$7.88.10^{7}$	$1.03.10^{9}$
7	$5.96.10^{-1}$	$6.42.10^{1}$	$8.00.10^{7}$	$1.04.10^{9}$
8	$6.81.10^{-1}$	-	-	-
9	$7.66.10^{-1}$	$6.42.10^{1}$	$8.05.10^{7}$	$1.04.10^{9}$
10	$8.51.10^{-1}$	$6.42.10^{1}$	$8.09.10^{7}$	$1.04.10^{9}$
11	$9.37.10^{-1}$	-	-	-
12	1.02	$6.42.10^{1}$	$7.75.10^{7}$	$1.03.10^{9}$
Mean	-	64.20341	-	-
sd(yEr±)	-	0	-	-
se(yEr±)	-	0	-	-

Table S7
Data on normality Shapiro-Wilk test of mass spectrometric measurands summarized in **Tables S2–S4**

Dataset	N	W	P value	Decision
**Data on Table S3 CE=10 V**				
PARA66CE10_L	12	0.97308	0.89546	Normal at 0.05 level
PARA66CE10_G	12	0.90536	0.17743	Normal at 0.05 level
PARA66CE10_20637681.9906	11	0.81760	0.01736	Not Normal at 0.05 level
PARA66CE10_20637681.9906	10	0.90135	0.21628	Normal at 0.05 level
PARA66CE10_2A	7	0.92164	0.50018	Normal at 0.05 level
PARA66CE10_N	12	0.92300	0.29422	Normal at 0.05 level
PARA66CE10_3A	8	0.90357	0.31543	Normal at 0.05 level
PARA66CE10_P	10	0.97014	0.88353	Normal at 0.05 level
PARA66CE10_Q	10	0.89434	0.18080	Normal at 0.05 level
PARA66CE10_T	10	0.98533	0.98529	Normal at 0.05 level
PARA66CE10_U	6	0.93247	0.61052	Normal at 0.05 level
PARA66CE10_W	7	0.76619	0.01832	Not Normal at 0.05 level
**Data on Table S4** C=10V	12	0.85152	0.03572	Not Normal at 0.05 level
**Data on Table S3** m/z 63 (CE=20 V)	12	0.90426	0.17180	Normal at 0.05 level
**Data on Table S2**				
QC_H_SRM_CE40_3_1	12	0.76507	0.00294	Not Normal at 0.05 level
QC_H_SRM_CE40_3_2	6	0.76215	0.02618	Not Normal at 0.05 level
QC_H_SRM_CE40_3_3	14	0.94188	0.42355	Normal at 0.05 level
QC_H_SRM_CE40_2_1	12	0.90861	0.19506	Normal at 0.05 level
QC_H_SRM_CE40_2_2	6	0.96423	0.85401	Normal at 0.05 level
QC_H_SRM_CE40_1	6	0.81506	0.07350	Normal at 0.05 level

Table S8

Chemometrics of one-way ANOVA tests of mass spectrometric measurands of analytes listed in **Tables S2–S4** and **S9**

Chemometrics of one-way ANOVA tests of mass spectrometric measurands of analyte BAC-C12 listed in **Tables S2–S4** and **S9**

**Data on Table S3** and **S4** CE=20V m/z 63					
Dataset	N	Mean	SD	SE	
Data9_E	12	63.87054	0.00339	$9.77226.10^{-4}$	
Data9_F	6	63.82711	0	0	
H0: The means of all selected datasets are equal					
H1: The means of one or more selected datasets are different					
Source	DoF	Sum of Squares	Mean Square	F value	P value
Model	1	0.00754495216	0.00754495216	957.66335	$9.99201.10^{-16}$
Error	16	$1.26056024.10^{-4}$	$7.87850150.10^{-6}$		
At the 0.01 level, the population means are significantly different.					
Levene's Test for Equal Variance					
Source	DoF	Sum of Squares	Mean Square	F value	P value
Model	1	$4.41392255.10^{-10}$	$4.41392255.10^{-10}$	2.34266	0.14540
Error	16	$3.01464036.10^{-9}$	$1.88415022.10^{-10}$		
At the 0.01 level, the population means are not significantly different.					
Brown-Forsythe's Test for Equal Variance					
Source	DoF	Sum of Squares	Mean Square	F value	P value
Model	1	$2.48234591.10^{-5}$	$2.48234591.10^{-5}$	6.72027	0.01964
Error	16	$5.91011141.10^{-5}$	$3.69381963.10^{-6}$		
At the 0.01 level, the population means are not significantly different.					
Means Comparison using Bonferroni Test					
Dataset	Mean	Difference between Means	Simultaneous Confidence Intervals		Significant at 0.01 Level
Data9_E	63.87054	0.04343	Lower Limit	Upper Limit	Yes
Data9_F	63.82711		0.03933	0.04753	
**Data on Table S4** m/z 83 **CE=10 V**					
Dataset	N	Mean	SD	SE	
Data3_C	12	82.64095	0		
Data3_E	12	83.2992	0.01475	0.00426	
H0: The means of all selected datasets are equal					
H1: The means of one or more selected datasets are different					
Source	DoF	Sum of Squares	Mean Square	F value	P value
Model	1	2.59970115	2.59970115	23882.60754	0
Error	22	0.00239477306	$1.08853321.10^{-4}$		
At the 0.01 level, the population means are significantly different.					
Levene's Test for Equal Variance					
Source	DoF	Sum of Squares	Mean Square	F value	P value

Model	1	$2.38955751.10^{-7}$	$2.38955751.10^{-7}$	7.25497	0.01327
Error	22	$7.24610146.10^{-7}$	$3.29368248.10^{-8}$		
At the 0.01 level, the population means are not significantly different.					
Brown-Forsythe's Test for Equal Variance					
Source	DoF	Sum of Squares	Mean Square	F value	P value
Model	1	$7.83017963.10^{-4}$	$7.83017963.10^{-4}$	11.90911	0.00228
Error	22	0.00144648925	$6.57495115.10^{-5}$		
At the 0.01 level, the population means are significantly different.					
Means Comparison using Bonferroni Test					
Dataset	Mean	Difference between Means	Simultaneous Confidence Intervals		Significant at 0.01 Level
Data9_E	82.64095	-0.65824	Lower Limit	Upper Limit	Yes
Data9_F	83.2992		-0.67025	-0.64624	
**Data on two sets at m/z 152 at CE=30V (PARA)**					
Dataset	N	Mean	SD	SE	
Data10_I	5	152.01433	0.00812	0.00363	
Data10_Q	5	151.85536	0.01033	0.00462	
H0: The means of all selected datasets are equal					
H1: The means of one or more selected datasets are different					
Source	DoF	Sum of Squares	Mean Square	F value	P value
Model	1	0.0631851966	0.0631851966	731.64417	$3.7585.10^{-9}$
Error	8	$6.90884436.10^{-4}$	$8.63605545.10^{-5}$		
At the 0.01 level, the population means are significantly different.					
Levene's Test for Equal Variance					
Source	DoF	Sum of Squares	Mean Square	F value	P value
Model	1	$2.66093380.10^{-9}$	$2.66093380.10^{-9}$	1.11513	0.32181
Error	8	$1.90896878.10^{-8}$	$2.38621097.10^{-9}$		
At the 0.01 level, the population means are not significantly different.					
Brown-Forsythe's Test for Equal Variance					
Source	DoF	Sum of Squares	Mean Square	F value	P value
Model	1	$7.23684195.10^{-6}$	$7.23684195.10^{-6}$	0.17716	0.68490
Error	8	$3.26788190.10^{-4}$	$4.08485238.10^{-5}$		
At the 0.01 level, the population means are not significantly different.					
Means Comparison using Bonferroni Test					
Dataset	Mean	Difference between Means	Simultaneous Confidence Intervals		Significant at 0.01 Level
Data10_I	152.01433	0.15898	Lower Limit	Upper Limit	Yes
Data10_Q	151.85536		0.13926	0.1787	
**Data on Table S2**					
Dataset	N	Mean	SD	SE	
QC_H_SRM_CE40_3_1	6	110.8449	0.05985	0.02443	

QC_H_SRM_CE40_3_2	12	110.18229	0.04231	0.01221	
H0: The means of all selected datasets are equal					
H1: The means of one or more selected datasets are different					
Source	DoF	Sum of Squares	Mean Square	F value	P value
Model	1	1.75618348	1.75618348	747.37541	$7.43849.10^{-15}$
Error	16	0.0375968157	0.00234980098		
At the 0.01 level, the population means are significantly different.					
Levene's Test for Equal Variance					
Source	DoF	Sum of Squares	Mean Square	F value	P value
Model	1	$7.22855036.10^{-6}$	$7.22855036.10^{-6}$	0.52020	0.48116
Error	16	$2.22332671.10^{-4}$	$1.38957919.10^{-5}$		
At the 0.01 level, the population means are not significantly different.					
Brown-Forsythe's Test for Equal Variance					
Source	DoF	Sum of Squares	Mean Square	F value	P value
Model	1	$3.47161659.10^{-4}$	$3.47161659.10^{-4}$	0.21204	0.65137
Error	16	0.0261958967	0.00163724354		
At the 0.01 level, the population means are not significantly different.					
Means Comparison using Bonferroni Test					
Dataset	Mean	Difference between Means	Simultaneous Confidence Intervals		Significant at 0.01 Level
QC_H_SRM_CE40_3_1	110.8449	0.66261	Lower Limit	Upper Limit	Yes
QC_H_SRM_CE40_3_2	110.18229		0.59181	0.7334	
Dataset	N	Mean	SD	SE	
QC_H_SRM_CE40_3_1	12	110.18229	0.04231	0.01221	
QC_H_SRM_CE40_3_3	14	110.06525	0.04709	0.01259	
H0: The means of all selected datasets are equal					
H1: The means of one or more selected datasets are different					
Source	DoF	Sum of Squares	Mean Square	F value	P value
Model	1	0.0885082249	0.0885082249	43.78559	$7.62697.10^{-7}$
Error	24	0.0485136226	0.00202140094		
At the 0.01 level, the population means are significantly different.					
Levene's Test for Equal Variance					
Source	DoF	Sum of Squares	Mean Square	F value	P value
Model	1	$1.13118442.10^{-6}$	$1.13118442.10^{-6}$	0.13305	0.71849
Error	24	$2.04047174.10^{-4}$	$8.50196557.10^{-6}$		
At the 0.01 level, the population means are not significantly different.					
Brown-Forsythe's Test for Equal Variance					
Source	DoF	Sum of Squares	Mean Square	F value	P value
Model	1	$5.31519368.10^{-4}$	$5.31519368.10^{-4}$	0.49836	0.48702
Error	24	0.0255969510	0.00106653963		
At the 0.01 level, the population means are not significantly different.					
Means Comparison using Bonferroni Test					

Dataset	Mean	Difference between Means	Simultaneous Confidence Intervals		Significant at 0.01 Level
QC_H_SRM_CE40_3_1	110.18229	0.11704	Lower Limit	Upper Limit	Yes
QC_H_SRM_CE40_3_3	110.06525		0.06757	0.16651	
Dataset	N	Mean	SD	SE	
QC_H_SRM_CE40_3_3	12	110.06211	0.03667	0.01058	
QC_H_SRM_CE40_2_1	14	110.06525	0.04709	0.01259	
H0: The means of all selected datasets are equal					
H1: The means of one or more selected datasets are different					
Source	DoF	Sum of Squares	Mean Square	F value	P value
Model	1	$6.37970196 \cdot 10^{-5}$	$6.37970196 \cdot 10^{-5}$	0.03511	0.85295
Error	24	0.0436146664	0.00181727777		
At the 0.01 level, the population means are not significantly different.					
Levene's Test for Equal Variance					
Source	DoF	Sum of Squares	Mean Square	F value	P value
Model	1	$4.41552745 \cdot 10^{-6}$	$4.41552745 \cdot 10^{-6}$	0.79621	0.38109
Error	24	$1.33097146 \cdot 10^{-4}$	$5.54571442 \cdot 10^{-6}$		
At the 0.01 level, the population means are not significantly different.					
Brown-Forsythe's Test for Equal Variance					
Source	DoF	Sum of Squares	Mean Square	F value	P value
Model	1	$7.42965461 \cdot 10^{-5}$	$7.42965461 \cdot 10^{-5}$	0.12224	0.72967
Error	24	0.0145873771	$6.07807379 \cdot 10^{-4}$		
At the 0.01 level, the population means are not significantly different.					
Means Comparison using Bonferroni Test					
Dataset	Mean	Difference between Means	Simultaneous Confidence Intervals		Significant at 0.01 Level
QC_H_SRM_CE40_3_3	110.06211	-0.00314	Lower Limit	Upper Limit	No
QC_H_SRM_CE40_2_1	110.06525		-0.05005	0.04376	
Dataset	N	Mean	SD	SE	
QC_H_SRM_CE40_3_3	6	110.08594	0.03017	0.01232	
QC_H_SRM_CE40_1	14	110.06525	0.04709	0.01259	
H0: The means of all selected datasets are equal					
H1: The means of one or more selected datasets are different					
Source	DoF	Sum of Squares	Mean Square	F value	P value
Model	1	0.00179748638	0.00179748638	0.96936	0.33789
Error	18	0.0333773179	0.00185429544		
At the 0.01 level, the population means are not significantly different.					
Levene's Test for Equal Variance					
Source	DoF	Sum of Squares	Mean Square	F value	P value
Model	1	$7.10358857 \cdot 10^{-6}$	$7.10358857 \cdot 10^{-6}$	1.00915	0.32842
Error	18	$1.26705329 \cdot 10^{-4}$	$7.03918496 \cdot 10^{-6}$		
At the 0.01 level, the population means are not significantly different.					
Brown-Forsythe's Test for Equal Variance					

Source	DoF	Sum of Squares	Mean Square	F value	P value
Model	1	0.00106092280	0.00106092280	1.26110	0.27620
Error	18	0.0151428370	$8.41268724.10^{-4}$		
At the 0.01 level, the population means are not significantly different.					
Means Comparison using Bonferroni Test					
Dataset	Mean	Difference between Means	Simultaneous Confidence Intervals		Significant at 0.01 Level
QC_H_SRM_CE40_3_3	110.08594	0.02069	Lower Limit	Upper Limit	No
QC_H_SRM_CE40_1	110.06525		-0.03979	0.08117	
**Data on Table S9**					
Dataset	N	Mean	SD	SE	
DataC12_S1_212	32	212.22955	0.13451	0.02378	
DataC12_S1_211	11	211.83358	0.1345	0.04055	
H0: The means of all selected datasets are equal					
H1: The means of one or more selected datasets are different					
Source	DoF	Sum of Squares	Mean Square	F value	P value
Model	1	1.28346677	1.28346677	70.93881	$1.7549.10^{-10}$
Error	41	0.741796143	0.0180925889		
At the 0.01 level, the population means are significantly different.					
Levene's Test for Equal Variance					
Source	DoF	Sum of Squares	Mean Square	F value	P value
Model	1	$9.61487464.10^{-6}$	$9.61487464.10^{-6}$	0.04427	0.83440
Error	41	0.00890492586	$2.17193314.10^{-4}$		
At the 0.01 level, the population means are not significantly different.					
Brown-Forsythe's Test for Equal Variance					
Source	DoF	Sum of Squares	Mean Square	F value	P value
Model	1	$1.53935454.10^{-4}$	$1.53935454.10^{-4}$	0.02721	0.86980
Error	41	0.231990938	0.00565831555		
At the 0.01 level, the population means are not significantly different.					
Means Comparison using Bonferroni Test					
Dataset	Mean	Difference between Means	Simultaneous Confidence Intervals		Significant at 0.01 Level
DataC12_S1_212	212.22955	0.39596	Lower Limit	Upper Limit	Yes
DataC12_S1_211	211.83358		0.26897	0.52295	
Dataset	N	Mean	SD	SE	
DataC12_S2_212	33	**212.21401**	0.1001	0.01742	
DataC12_S2_213	8	**212.54356**	0.12232	0.04325	
H0: The means of all selected datasets are equal					
H1: The means of one or more selected datasets are different					
Source	DoF	Sum of Squares	Mean Square	F value	P value
Model	1	0.699337587	0.699337587	64.11999	$9.25001.10^{-10}$

Error	39	0.425361365	0.0109067017		
**At the 0.01 level, the population means are significantly different.**					
Levene's Test for Equal Variance					
Source	DoF	Sum of Squares	Mean Square	F value	P value
Model	1	$7.33429730.10^{-5}$	$7.33429730.10^{-5}$	0.54014	0.46677
Error	39	0.00529561530	$1.35785008.10^{-4}$		
At the 0.01 level, the population means are not significantly different.					
Brown-Forsythe's Test for Equal Variance					
Source	DoF	Sum of Squares	Mean Square	F value	P value
Model	1	$1.49330356.10^{-5}$	$1.49330356.10^{-5}$	0.00382	0.95101
Error	39	0.152319014	0.00390561574		
At the 0.01 level, the population means are not significantly different.					
Means Comparison using Bonferroni Test					
Dataset	Mean	Difference between Means	Simultaneous Confidence Intervals		Significant at 0.01 Level
DataC12_S2_212	212.21401		Lower Limit	Upper Limit	Yes
DataC12_S2_213	212.54356	-0.32956	-0.44101	-0.21811	
Dataset	N	Mean	SD	SE	
DataC12_S1_212.2	32	**212.22955**	0.13451	0.02378	
DataC12_S2_212.2	33	**212.21401**	0.1001	0.01742	
H0: The means of all selected datasets are equal					
H1: The means of one or more selected datasets are different					
Source	DoF	Sum of Squares	Mean Square	F value	P value
Model	1	0.00392362981	0.00392362981	0.28041	0.59830
Error	63	0.881538627	0.0139926766		
**At the 0.01 level, the population means are not significantly different.**					
Levene's Test for Equal Variance					
Source	DoF	Sum of Squares	Mean Square	F value	P value
Model	1	$9.91504621.10^{-4}$	$9.91504621.10^{-4}$	5.42690	0.02305
Error	63	0.0115102059	$1.82701681.10^{-4}$		
At the 0.01 level, the population means are not significantly different.					
Brown-Forsythe's Test for Equal Variance					
Source	DoF	Sum of Squares	Mean Square	F value	P value
Model	1	0.0142792462	0.0142792462	3.12902	0.08175
Error	63	0.287499987	0.00456349186		
At the 0.01 level, the population means are not significantly different.					
Means Comparison using Bonferroni Test					
Dataset	Mean	Difference between Means	Simultaneous Confidence Intervals		Significant at 0.01 Level
DataC12_S1_212.2	212.22955		Lower Limit	Upper Limit	No
DataC12_S2_212.2	212.21401	0.01554	-0.06241	0.09349	

Table S9

Temporal distribution of measurands at m/z 212 of SRM spectra of biocides in biota of CID-MS spectra of BAC-C12 of molecular cation $[M+H]^+$ at m/z 304 with respect to analyte concentration $[pg.(mL)^{-1}]$ (experimental dataset Cali_Si_1.raw; i = 1–8 [101,102];) descriptive statistics; absolute intensity (I [arb.units]) and m/z data on span of scan time shown as scan number; D''_{SD} parameter $[cm^2.s^{-1}]$ according to equation (2)

S1	c=2 ng.mL^{-1}				S2	c=6 ng.mL^{-1}					
Scan	m/z	I [arb.units]	m/z	I [arb.units]	Scan	m/z	I [arb.units]	m/z	I [arb.units]	m/z	I [arb.units]
2854	212.2647	4051.0846	-	-	2685	212.40133	2693.88723	-	-	-	-
3154	212.13495	3101.11104	-	-	2845	-	-	-	-	211.76701	1408.65281
3178	212.43479	1418.98974	-	-	2953	-	-	212.44894	3340.52234	-	-
3183	-	-	211.96514	3159.58568	3037	212.26799	3115.14476	-	-	-	-
3186	-	-	211.97918	2577.39639	3093	212.13379	2523.86702	-	-	-	-
3190	212.16807	4059.65649	-	-	3153	212.09847	1951.48393	-	-	-	-
3202	212.09258	2308.61058	-	-	3196	212.26673	2350.42418	-	-	211.80172	3549.61603
3206	212.40037	2681.92471	-	-	3173	-	-	212.46245	2614.9418	211.92965	1570.3656
3210	212.17021	2940.46389	-	-	3181	212.37275	635.29011	-	-	211.76526	1297.21493
3246	212.04789	2415.54717	-	-	3185	212.18078	301.95997	-	-	-	-
3378	-	-	211.73506	255.36377	3189	212.27044	423.06994	-	-	-	-
3609	212.23212	1666.30712	-	-	3197	-	-	-	-	211.88217	1279.87981
3697	212.07821	764.3808	211.65909	2986.56489	3201	-	-	212.7663	2655.94675	-	-
4033	-	-	211.95708	3042.29993	3213	-	-	-	-	211.96572	1747.20029
4321	212.36258	2406.37874	-	-	3596	-	-	-	-	211.79763	2983.11331
4481	-	-	211.96904	5937.19738	3652	212.17924	2521.23726	-	-	-	-
4609	212.16976	2674.27227	-	-	3716	212.09519	1530.65349	-	-	-	-
4665	-	-	211.69999	2019.99172	3876	-	-	-	-	211.63544	1975.83541
4729	212.10303	4938.57779	-	-	4044	212.16728	2597.33007	-	-	-	-
4937	212.10117	2724.45401	-	-	4092	-	-	212.52426	1491.63613	-	-
5129	212.16203	2372.30234	-	-	4100	212.23554	2021.41305	-	-	-	-
5385	212.16687	2067.16943	-	-	4204	-	-	212.43575	3094.47877	-	-
5489	212.02949	1837.8692	-	-	4276	-	-	-	-	211.59759	2242.23458
5521	212.00234	4244.81845	-	-	4308	212.35991	2650.38917	-	-	-	-
5545	212.43379	2159.13998	-	-	4380	212.09909	1851.51872	-	-	-	-
5633	212.19522	2611.44726	-	-	4404	-	-	-	-	211.86971	5058.17747
5649	212.38115	4058.89799	-	-	4644	-	-	-	-	211.86501	1273.24676
5785	212.3359	969.81379	-	-	4692	212.29722	2052.52072	-	-	-	-
5889	212.33042	2916.71964	-	-	4748	212.30127	1993.25442	-	-	-	-
5953	212.18298	2256.66052	-	-	4820	-	-	-	-	211.90172	2120.96072

5977	212.13354	2834.39223	-	-	4828	212.18529	3242.5939	-	-	-	-
5993	-	-	211.66897	1760.36314	4932	-	-	212.48145	7713.34822	-	-
6509	-	-	211.76825	2018.22117	5012	212.10261	2473.21074	-	-	-	-
6017	212.31696	3229.91704	-	-	5052	-	-	-	-	211.36591	1531.44081
6081	212.34983	3163.71045	-	-	5084	-	-	-	-	211.82741	4587.2
6113	-	-	211.79904	2925.49047	5108	212.03141	4256.97119	-	-	-	-
6121	212.30111	5096.32918	-	-	5212	212.0327	2614.55104	-	-	-	-
6129	212.26296	1642.77406	-	-	5388	212.26636	2383.5337	-	-	-	-
6161	212.44342	2640.62536	-	-	5484	212.10692	845.04073	-	-	-	-
6193	-	-	211.96858	2177.88846	5524	212.26732	2897.59572	-	-	-	-
6329	212.09694	1768.11727	-	-	5584	212.19753	2733.23996	-	-	-	-
-	-	-	-	-	5716	212.16657	2299.49859	-	-	-	-
-	-	-	-	-	5764	212.13289	3184.13798	-	-	-	-
-	-	-	-	-	5796	212.37221	1444.0719	-	-	211.70252	2376.00533
-	-	-	-	-	5836	212.23249	1695.75735	-	-	-	-
-	-	-	-	-	5900	212.29685	1474.45806	-	-	-	-
-	-	-	-	-	6004	--	--	-	-	211.90161	1778.79065
-	-	-	-	-	6012	212.26957	3165.42986	-	-	-	-
-	-	-	-	-	6060	--	--	212.53218	1828.11014	-	-
-	-	-	-	-	6108	212.30126	3053.98342	-	-	-	-
-	-	-	-	-	6164	212.26886	3802.55533	-	-	-	-
-	-	-	-	-	6220	212.10432	2723.31641	-	-	-	-
-	-	-	-	-	6540	-	-	212.69718	2462.21797	-	-
Mean	212.22211	-	-	-	Mean	212.21401	-	212.54356	-	211.786	-
sd(yEr±)	0.12988	-	-	-	sd(yEr±)	0.1001	-	0.12231	-	0.15203	-
se(yEr±)	0.02333	-	-	-	se(yEr±)	0.01742	-	0.04324	-	0.03801	-
<I>	-	2710.40204	-	-	<I>	-	2287.98151	-	3150.15027	-	2298.74591
<I>^{2}	-	7346279.2184	-	-	<I>^{2}	-	5234859.39	-	9923446.7236	-	5284232.7587
<I^2>	-	$8.40906.10^6$	-	-	<I^2>	-	$6.03463.10^6$	-	$1.3219.10^7$	-	$6.57226.10^6$
<I^2>–<I>^{2}	-	1062780.7816	-	-	<I^2>–<I>^{2}	-	799770.61	-	3295553.276	-	1288027.2413
**D''_{SD}**	**$2.8044.10^{-11}$**		-	-	**D''_{SD}**	**$2.11.10^{-11}$**		**$8.6963.10^{-11}$**		**$3.3989.10^{-11}$**	
S3	c=10 ng.mL^{-1}		S4	c=20 ng.mL^{-1}		S5	c=30 ng.mL^{-1}		S6	c=50 ng.mL^{-1}	
Scan	m/z	I [arb.units]	Scan	m/z	I [arb.units]	Scan	m/z	I [arb.units]	Scan	m/z	I [arb.units]
2796	212.36035	3529.30381	3159	212.10354	934.27119	3022	212.13135	2449.56521	3136	212.01748	4017.03845
2900	212.1901	2655.62232	3163	212.08407	14836.5133	3142	212.16777	1884.32324	3140	212.00116	2332.15386
3092	212.38002	3422.51848	3167	212.15475	17090.2865	3146	212.23172	7123.28329	3144	212.16056	8007.9737
3144	212.36448	1798.26498	3171	212.18722	33328.1426	3150	212.24006	16969.7957	3148	212.07434	19693.8328

3164	212.26088	4447.96225	3175	212.11949	13335.9287	3162	212.15232	21504.999	3152	212.1237	51114.8938
3172	212.28852	11131.4411	3174	212.12236	6989.5054	3166	212.18892	17670.0941	3156	212.08778	54381.1503
3176	212.13465	7675.39143	3183	212.23316	521.16888	3170	212.19078	6894.07387	3160	212.18865	60677.0017
3188	212.10103	269.92846	3187	212.33416	2772.194	3174	212.19063	7063.36639	3164	212.10136	14280.5384
3192	212.09149	1776.31682	3191	212.03004	1908.08967	3178	212.0674	6172.62766	3168	212.12443	17911.067
3196	212.36312	1861.7289	3195	212.30013	6022.54688	3182	212.14513	3567.39769	3172	212.19584	8580.50881
3236	212.3996	3201.38155	3207	212.2	2310.00595	3186	212.13039	4714.39448	3176	212.33965	6580.39985
3252	212.29033	1557.94225	3878	212.06776	2085.56045	3190	212.10242	3782.72271	3180	212.16544	3519.20431
3332	212.37022	2866.38369	3424	212.1345	3147.55397	3198	212.27992	2094.48027	3188	212.23392	2200.78853
3498	212.06861	3733.47679	3454	212.23604	2699.40128	3206	212.30874	3956.01975	3196	212.16653	5136.26643
3439	212.26492	1576.94355	3526	212.33538	2723.77076	3218	212.33182	3128.35295	3224	212.20262	3300.5971
3983	212.09926	2855.36283	3558	212.22839	2492.80301	3222	212.13453	1373.76301	3236	212.30427	2287.95359
4071	212.43423	2463.64555	3687	212.30112	2608.2017	3226	212.23388	2059.65393	3240	212.16516	3203.69869
4199	212.02695	6400.0026	3678	212.30112	2608.2017	3242	212.10246	3060.16775	3264	212.40041	2744.46573
4431	212.1037	2985.35983	4064	212.19519	4502.94194	3250	212.06663	2527.56092	3268	212.03229	2402.17115
4592	212.29647	1800.83157	4446	212.4193	1314.77732	3262	212.1362	3323.75253	3312	212.10115	2557.75295
4607	212.27041	3151.90401	4630	212.00038	1479.06771	3266	212.30048	2143.7125	3324	212.19401	1742.2717
4663	212.43395	2638.43017	4750	212.43362	1529.43659	3310	212.42119	3634.24117	3332	212.23218	4118.76874
4693	212.0908	2098.93748	5014	212.35875	2000.00681	3318	212.18192	3058.11002	3411	212.32868	2846.36732
4927	212.32463	2000.58699	5078	212.16525	2359.36284	3334	212.40065	2932.67001	3547	212.00347	3808.72342
4975	212.42436	1902.16668	5230	212.22468	1362.59838	3370	212.10303	3510.76926	3571	212.36593	2254.57864
5007	212.24467	7811.1044	5246	212.23474	2067.72728	3429	212.16324	1593.06508	3603	212.10142	1823.55754
5127	212.18877	2722	5270	212.03134	2963.82686	3781	212.18358	2879.35675	3611	212.25043	1557.34801
5183	212.30447	3943.07719	5310	212.06786	2351.46248	3909	212.23061	3647.79437	3635	212.23805	3993.90187
5351	212.06798	3872.52345	5390	212.30006	2569.81424	4157	212.19796	3169.55146	3715	212.23636	3190.27128
5447	212.16556	1936.15257	5470	212.06595	2145.24185	4269	212.10197	2499.09424	3731	212.23041	3368.228
5463	212.17556	528.03496	5526	212.32026	2970.50725	4469	212.40002	2415.78277	3891	212.22041	2590.17451
5567	212.43527	2976.46891	5578	212.29021	1073.46527	4613	212.26486	4145.86573	4067	212.06619	2674.69014
5183	212.26416	3695.7769	5702	212.13848	3628.12217	4629	212.26641	2447.22286	4283	212.1132	1812.04024
5351	212.20216	2389.56537	5822	212.20143	2476.04868	4661	212.42824	2238.13627	4371	212.32238	4710.71609
5447	212.37406	2560.18962	5910	212.32773	2732.72224	4717	212.13294	2273.35291	4387	212.1828	1511.5386
5463	212.25355	1760.85077	5926	212.22309	2389.69491	4997	212.26524	2680.9065	4403	212.43104	1468.52533
5567	212.2131	5454.09859	5982	212.23569	3855.84514	5021	212.12943	2660.19582	4411	212.22715	3027.08209
5663	212.19043	2291.26599	-	-	-	5237	212.24944	1846.68779	4531	212.3711	3174.39555
5727	212.466	1469.67953	-	-	-	5253	212.19268	3488.14472	4603	212.33066	1403.49842
-	-	-	-	-	-	5261	212.16082	2126.02861	4651	212.37881	1789.64369
-	-	-	-	-	-	5277	212.18973	2469.46735	4915	212.09708	3094.99313

-	-	-	-	-	-	5461	212.39999	1501.06923	4923	212.03358	2915.3835
-	-	-	-	-	-	5693	212.20548	4945.09089	5083	212.28072	3419.95603
-	-	-	-	-	-	5709	212.09998	2264.24669	5203	212.11384	1974.60569
-	-	-	-	-	-	5821	212.16584	3448.57928	5427	212.03731	4160.33454
-	-	-	-	-	-	5997	212.06726	4045.82611	5531	212.4331	1815.96546
-	-	-	-	-	-	6077	212.26672	4926.4307	5547	212.2638	2125.19921
-	-	-	-	-	-	6101	212.33314	2301.41788	5571	212.20095	2138.32749
-	-	-	-	-	-	6149	212.16865	2720.43066	6011	212.22779	1858.02525
-	-	-	-	-	-	6301	212.135	2991.70549	6016	212.16507	1565.71285
-	-	-	-	-	-	-	-	-	6035	212.20223	3546.5663
-	-	-	-	-	-	-	-	-	6099	212.16957	2237.08295
Mean	212.25587	-	Mean	212.2083	-	Mean	212.20679	-	Mean	212.19685	-
sd(yEr±)	0.12279	-	sd(yEr±)	0.11115	-	sd(yEr±)	0.09604	-	sd(yEr±)	0.11291	-
se(yEr±)	0.01966	-	se(yEr±)	0.01827	-	se(yEr±)	0.01358	-	se(yEr±)	0.01566	-
$\langle I\rangle$	-	3159.29801	$\langle I\rangle$	-	4437.48151	$\langle I\rangle$	-	4166.50695	$\langle I\rangle$	-	6897.07559
$\langle I\rangle^2$	-	9981163.916	$\langle I\rangle^2$	-	19691242.1	$\langle I\rangle^2$	-	17359780.1644	$\langle I\rangle^2$	-	47569651.694
$\langle I^2\rangle$	-	$1.42402.10^{7}$	$\langle I^2\rangle$	-	$5.6151.10^{7}$	$\langle I^2\rangle$	-	$3.28934.10^{7}$	$\langle I^2\rangle$	-	$2.05822.10^{8}$
$\langle I^2\rangle–\langle I\rangle^2$	-	4259036.084	$\langle I^2\rangle–\langle I\rangle^2$	-	36459857.8	$\langle I^2\rangle–\langle I\rangle^2$	-	15533619.836	$\langle I^2\rangle–\langle I\rangle^2$	-	158252348.31
**D''_{SD}**	**$1.1238744.10^{-10}$**		**D''_{SD}**	**$9.6210.10^{-10}$**		**D''_{SD}**	**$4.09901.10^{-10}$**		**D''_{SD}**	**$4.17596.10^{-9}$**	
S7	c=70 ng.mL^{-1}		S8	c=80 ng.mL^{-1}		-	-	-	-	-	-
Scan	m/z	I [arb.units]	Scan	m/z	I [arb.units]	-	-	-	-	-	-
3049	212.1317	6396.47915	2687	212.38758	2742.23169	-	-	-	-	-	-
3125	212.16651	1900.42696	3119	212.20695	3099.43547	-	-	-	-	-	-
3141	212.16951	8109.87134	3123	212.1213	18510.8069	-	-	-	-	-	-
3145	212.19282	66803.5065	3127	212.06109	28496.795	-	-	-	-	-	-
3149	212.17177	139030.853	3131	212.20712	98412.6146	-	-	-	-	-	-
3153	212.18772	81686.7087	3135	212.18653	123598.08	-	-	-	-	-	-
3157	212.0745	98966.2602	3139	212.10806	119256.864	-	-	-	-	-	-
3161	212.18023	76561.504	3143	212.04806	73316.3967	-	-	-	-	-	-
3165	212.19136	33120.7546	3147	212.17101	57546.0815	-	-	-	-	-	-
3169	212.23036	14244.414	3151	212.17687	38197.5948	-	-	-	-	-	-
3173	212.12144	5408.77936	3155	212.24443	15573.9236	-	-	-	-	-	-
3177	212.17521	6102.81119	3159	212.1657	12129.968	-	-	-	-	-	-
3181	212.2802	4955.9918	3163	212.00418	2307.35477	-	-	-	-	-	-
3185	212.3685	867.00993	3167	212.3689	8921.30319	-	-	-	-	-	-
3189	212.26389	2580.99857	3171	212.2718	2802.95438	-	-	-	-	-	-
3197	212.26902	2620.03766	3175	212.16898	749.59743	-	-	-	-	-	-

3201	212.1335	1858.22291	3179	212.30202	9364.41896	-	-	-	-	-	-
3205	212.32561	2822.13159	3183	212.33901	9111.46378	-	-	-	-	-	-
3209	212.14699	4274.16863	3199	212.36667	1377	-	-	-	-	-	-
3213	212.03502	1776.69109	3207	212.13613	5816.26332	-	-	-	-	-	-
3217	212.0129	4094.81887	3211	212.43434	1504.85716	-	-	-	-	-	-
3221	212.23289	2457.62146	3243	212.23507	2216.69287	-	-	-	-	-	-
3225	212.10308	4030.56741	3247	212.26442	266.75031	-	-	-	-	-	-
3237	212.26536	2542.67862	3251	212.06751	2620.92711	-	-	-	-	-	-
3273	212.14834	1798.74165	3255	212.20345	7330.60733	-	-	-	-	-	-
3277	212.26709	2938.9669	3267	212.26696	3912.5634	-	-	-	-	-	-
3293	212.13086	3443.30347	3299	212.11233	2166.19989	-	-	-	-	-	-
3305	212.402	3864.84259	3319	212.39403	2558.53847	-	-	-	-	-	-
3317	212.15456	2471.85345	3331	212.09041	3922.2934	-	-	-	-	-	-
3321	212.21038	4130.64578	3339	211.92431	1686.23027	-	-	-	-	-	-
3341	212.18979	2444.06574	3351	212.18174	2750.26883	-	-	-	-	-	-
3353	212.30611	4963.77814	3355	212.06267	2369.45365	-	-	-	-	-	-
3365	212.30061	3801.15191	3375	212.12824	2669.22339	-	-	-	-	-	-
3404	212.43302	1963.49437	3379	212.23554	2655.64169	-	-	-	-	-	-
3460	212.22539	1658.79031	3398	212.43705	2758.1202	-	-	-	-	-	-
3556	212.40512	1280.14051	3494	212.26404	1769.46792	-	-	-	-	-	-
3564	212.23395	1988.53758	3502	212.30171	1946.3269	-	-	-	-	-	-
3572	212.23312	2504.59481	3510	212.43265	2007.39144	-	-	-	-	-	-
3988	212.23417	3671.82238	3606	212.25265	3397.5556	-	-	-	-	-	-
4012	212.3644	2846.65683	3614	212.23411	2251.7443	-	-	-	-	-	-
4044	212.3621	1683.13244	3662	212.18871	2702.08334	-	-	-	-	-	-
4052	212.23415	1965.78485	3694	212.29986	2447.08135	-	-	-	-	-	-
4116	212.20126	2053.26939	3726	212.11392	2403.38564	-	-	-	-	-	-
4348	212.12785	2631.94814	3750	212.00376	6870.72008	-	-	-	-	-	-
4396	212.40084	1408.23234	3918	212.3861	1333.80115	-	-	-	-	-	-
4564	212.40287	3337.8482	4318	212.33454	2204.31562	-	-	-	-	-	-
4636	212.23271	2813.03092	4366	212.23364	2533.48364	-	-	-	-	-	-
4660	212.26828	624.25262	4406	212.06787	2773.91531	-	-	-	-	-	-
4708	212.16842	1819.10559	4518	212.23484	1971.24486	-	-	-	-	-	-
4860	212.31538	3641.57761	4798	212.20069	1590.84412	-	-	-	-	-	-
4940	212.15965	2590.11686	5062	212.36942	2494.04756	-	-	-	-	-	-
5124	212.04334	382.84293	5126	212.21918	3706.77647	-	-	-	-	-	-
5140	212.04378	2088.05092	5158	212.24409	4110.02143	-	-	-	-	-	-

5156	212.2008	1785.16626	5182	212.16579	1553.85611	-	-	-	-	-	-
5172	212.10166	1757.22952	5422	212.22655	2040.55326	-	-	-	-	-	-
5180	212.16796	2752.90103	5686	212.1017	1994.40443	-	-	-	-	-	-
5284	212.20065	3922.06189	5742	212.13195	2276.24733	-	-	-	-	-	-
5300	212.10114	1970.53003	5798	212.1302	1930.20967	-	-	-	-	-	-
5324	212.03481	2064.77114	5878	212.17067	2353.73681	-	-	-	-	-	-
5372	212.26948	5649.92706	6014	212.0974	2350.36235	-	-	-	-	-	-
5468	212.05459	1910.11717	6078	212.39527	1287.86495	-	-	-	-	-	-
5540	212.22783	1248.42191	6174	212.26538	3231.20885	-	-	-	-	-	-
5572	212.30249	3183.81885	-	-	-	-	-	-	-	-	-
5596	212.23102	2885.57895	-	-	-	-	-	-	-	-	-
5604	212.10096	1721.29119	-	-	-	-	-	-	-	-	-
5628	212.08343	2609.51684	-	-	-	-	-	-	-	-	-
5652	212.1012	2453.84305	-	-	-	-	-	-	-	-	-
5772	212.13467	2784.95997	-	-	-	-	-	-	-	-	-
5884	212.41503	3060.78125	-	-	-	-	-	-	-	-	-
5916	212.26368	1859.67882	-	-	-	-	-	-	-	-	-
5948	212.26815	3259.67362	-	-	-	-	-	-	-	-	-
6244	212.19964	2232.29608	-	-	-	-	-	-	-	-	-
Mean	212.20957	-	Mean	212.21205		-	-	-	-	-	-
sd(yEr±)	0.10179	-	sd(yEr±)	0.11644		-	-	-	-	-	-
se(yEr±)	0.012	-	se(yEr±)	0.01479		-	-	-	-	-	-
$\langle I \rangle$	-	9654.67293	$\langle I \rangle$	-	11939.5511	-	-	-	-	-	-
$\langle I \rangle^2$	-	93212709.3853	$\langle I \rangle^2$	-	142552879.75	-	-	-	-	-	-
$\langle I^2 \rangle$	-	$6.67692.10^{8}$	$\langle I^2 \rangle$	-	$8.31274.10^{8}$	-	-	-	-	-	-
$\langle I^2 \rangle – \langle I \rangle^2$	-	574479290.615	$\langle I^2 \rangle – \langle I \rangle^2$	-	688721120.25	-	-	-	-	-	-
**D''_{SD}**	**$1.5159359.10^{-8}$**		**D''_{SD}**	**$4.29679.10^{-8}$**		-	-	-	-	-	-

Table S10
Chemometric data on t-test of BAC-C12 at concentration c=2 ng.(mL)$^{-1}$ and based on measurands listed in **Table S9.**

Sample	N	Mean	sd(yEr±)	se(yEr±)
BAC-C12	32	212.22955	0.13451	0.02378
H_0: Mean=0				
H_1: Mean <>0				
t	DoF	P value		
8925.17224	31	$6.30589.10^{-101}$	-	-
At the 0.05 level, the population mean is significantly different than the test mean (0).				
Confidence Interval for Mean			-	-
Level	Lower Limit	Upper Limit	-	-
50	212.21332	212.24577	-	-
90	212.18923	212.26986	-	-
95	212.18105	212.27804	-	-
99.9	212.14315	212.31594	-	-
99.99	212.12351	212.33558	-	-

Table S11

Temporal distribution of measurands of m/z 240 of SRM spectra of biocides in biota of CID-MS spectra of BAC-C14 of molecular cation $[M+H]^+$ at m/z 332 with respect to analyte concentration $[pg.(mL)^{-1}]$ (experimental dataset Cali_Si_1.raw; i = 1–8 (15.08.2017) [101,102];) descriptive statistics; absolute intensity (I [arb.units]) and m/z data on span of scan time shown as scan number; D''_{SD} parameter $[cm^2.s^{-1}]$ according to equation (2)

S4	c=20 ng.mL^{-1}		S5	c=30 ng.mL^{-1}		S6	c=50 ng.mL^{-1}		S8	c=80 ng.mL^{-1}	
Scan	m/z	I [arb.units]	Scan	m/z	I [arb.units]	Scan	m/z	I [arb.units]	Scan	m/z	I [arb.units]
3463	240.15075	9933.11753	3438	240.196	1835.29969	3412	240.13396	3585.48485	3423	240.27017	6803.51777
3471	240.14643	12405.1126	3446	240.16785	3004.35631	3436	240.50096	2415.64292	3431	240.1702	35820.7672
3479	240.17359	18787.7172	3454	240.16482	26434.398	3444	240.0339	3989.16964	3439	240.19902	72621.7286
3487	240.23958	10654.8398	3462	240.11157	29118.1815	3452	240.27191	3462.1939	3447	240.14456	77841.7308
3495	240.12871	1625.92443	3470	240.13381	21818.9335	3460	240.10211	44201.5423	3455	240.17221	39077.1415
3519	240.02159	3632.86342	3476	240.16951	4792.92903	3468	240.1933	80410.8918	3463	240.13226	25396.0288
3535	240.20242	4811.41888	3494	240.06842	1995.19465	3476	240.14367	16439.8637	3471	240.09373	3163.3326
3543	240.03254	1905.78896	3502	240.30673	4976.03573	3484	240.33303	7258.28555	3479	240.33472	948.61705
3559	240.53722	4218.69675	3510	240.49822	2870.4843	3492	240.3275	2356.11293	3487	240.43484	3847.35887
3567	240.25921	2414.71884	3526	240.16872	2899.54277	3500	240.13527	4040.06977	3495	240.26856	4356.46596
3647	240.20154	1417.85791	3550	240.36106	2137.52618	3508	240.23249	5536.09694	3503	240.32897	3290.57946
3677	240.39356	3080.954	3582	240.1694	2717.64901	3516	240.12561	2418.01787	3535	240.36187	1481.85844
3719	240.06543	1728.00767	3598	240.51117	1133.61962	3524	240.26631	1013.47767	3543	240.26823	2372.9514
3783	240.401	2932.62407	4094	240.70243	3660.17639	3548	240.33298	3274.78147	3551	240.30039	921.96964
3951	240.60244	3065.82046	4270	240.36568	4643.14834	3556	240.0328	2435.16258	3559	240.3021	2291.55065
4239	240.13684	6755.25865	4342	240.03533	2220.46916	3572	240.16712	4632.16738	3567	240.16874	2109.3381
4743	240.03404	1965.71788	4470	240.00108	3362.84479	3604	240.19822	1155.02818	3575	240.32342	3947.46202
4823	240.38614	2811.91414	4742	240.16616	2545.79187	3676	240.20134	3443.02323	3583	240.16788	3544.16773
4831	240.09625	2980.51836	5014	240.12641	2146.335	3700	240.40912	4612.51893	3615	240.25824	1775.65343
4919	240.43435	3433.80224	5078	240.06217	1651.17939	3780	240.36643	1755.87428	3623	240.00079	2905.94718
4927	240.00167	3183.77475	5438	240.23126	1973.62472	3876	240.02117	967.68367	3711	240.36556	2415.20758
4975	240.13278	4077.91845	-	-	-	4132	240.1007	2487.29739	3871	240.20162	2331.98707
5063	240.33212	1684.26126	-	-	-	4172	240.13454	2943.05985	3991	240.10901	2302.92728
5423	240.10053	1401.47616	-	-	-	4196	240.20228	2572.06123	4207	240.06821	2932.16244
6137	240.43471	2396.2743	-	-	-	4204	240.30395	3276.87487	4231	240.25324	3164.73635
-	-	-	-	-	-	4228	240.26848	1902.03	4437	240.3057	1272.53747
-	-	-	-	-	-	4308	240.06813	1412.57097	4575	240.1768	2387.9086
-	-	-	-	-	-	4484	240.06776	1445.39091	4939	240.38838	1361.27109
-	-	-	-	-	-	4524	240.35139	2019.04319	4695	240.17389	2609.62317
-	-	-	-	-	-	4580	240.44617	3910.45456	4743	240.48378	1801.93279
-	-	-	-	-	-	4588	240.20296	5620.41567	4807	240.38594	2297.13747

-	-	-	-	-	-	4772	240.0342	2922.71427	4879	240.16803	2482.28934
-	-	-	-	-	-	5316	240.56825	2083.2933	4959	240.20049	3368.49441
-	-	-	-	-	-	5332	240.30087	2079.11406	4983	240.0957	2340.45539
-	-	-	-	-	-	5716	240.56775	2011.46012	5007	240.04783	2487.53611
-	-	-	-	-	-	5907	240.5974	3390.48804	5351	240.19099	1615.60112
-	-	-	-	-	-	6060	240.03302	2121.78477	5383	240.23257	1439.25521
-	-	-	-	-	-	-	-	-	5415	240.2683	3953.17487
Mean	240.22582	-	Mean	240.22466	-	Mean	240.23722	-	Mean	240.23202	-
sd(yEr±)	0.16946	-	sd(yEr±)	0.1758	-	sd(yEr±)	0.1608	-	sd(yEr±)	0.1106	-
se(yEr±)	0.03389	-	se(yEr±)	0.03836	-	se(yEr±)	0.02644	-	se(yEr±)	0.01794	-
<I>	-	4532.25515	<I>	-	6092.27238	<I>	-	6529.76061	<I>	-	8870.5896
<I>^{2}	-	20541336.745	<I>^{2}	-	37115782.8	<I>^{2}	-	42637773.624	<I>^{2}	-	78687359.852
<I^2>	-	3.73274.10^7	<I^2>	-	1.04077.10^8	<I^2>	-	2.44685.10^8	<I^2>	-	3.96249.10^8
<I^2>–<I>^{2}	-	16786063.255	<I^2>–<I>^{2}	-	66961217.3	<I^2>–<I>^{2}	-	202047226.38	<I^2>–<I>^{2}	-	317561640.15
**D$''_{SD}$**	**4.429506.10^{-10}**		**D$''_{SD}$**	**1.76697.10^{-9}**		**D$''_{SD}$**	**5.331622.10^{-9}**		**D$''_{SD}$**	**8.379817.10^{-9}**	

Table S12

Temporal distribution of measurands at m/z 211.8 of SRM spectra of biocides in biota of CID-MS spectra of BAC-C12 of molecular cation $[M+H]^+$ at m/z 304 with respect to analyte concentration [pg.$(mL)^{-1}$] (experimental dataset Cali_Si_1.raw; i = 1–8 [101,102];) descriptive statistics; absolute intensity (I [arb.units]) and m/z data on span of scan time shown as scan number; D''_{SD} parameter [$cm^2.s^{-1}$] according to equation (2)

S1	c=2 ng.mL^{-1}		S2	c=6 ng.mL^{-1}		S3	c=10 ng.mL^{-1}		S4	c=20 ng.mL^{-1}	
Scan	m/z	I [arb.units]	Scan	m/z	I [arb.units]	Scan	m/z	I [arb.units]	Scan	m/z	I [arb.units]
3182	211.96514	3159.58568	2845	211.76701	1408.65281	3168	211.56355	1734.84263	3171	211.58325	612.19412
3186	211.97918	2577.39639	3169	211.92965	1570.3656	3168	211.99061	3409.29575	*3179*	*211.33642*	*1191.77924*
3697	211.65909	2986.56489	3173	211.76577	1961.96968	3292	211.73241	2063.2971	3183	211.73378	2391.96591
4033	211.95708	3042.29993	3177	211.76526	1297.21493	3348	211.66526	3118.10668	3195	211.56417	2061.13146
4481	211.96904	5937.19738	3181	211.80172	3549.61603	3631	211.93207	2377.15228	3199	211.96708	1952.81575
4665	211.69999	2019.99172	3197	211.88217	1279.87981	4255	211.56838	2883.57215	3383	211.70054	2475.42965
-	-	-	3213	211.96572	1747.20029	4287	211.80091	1972.03478	3758	211.63362	2619.69266
-	-	-	3596	211.79763	2983.11331	4759	211.74131	4652.19852	4006	211.9627	5429.45362
-	-	-	3876	211.63544	1975.83541	-	-	-	5286	211.93376	2035.54698
-	-	-	4276	211.59759	2242.23458	-	-	-	5406	211.75759	2796.37844
-	-	-	4404	211.86971	5058.17747	-	-	-	5710	211.99912	3057.57035
-	-	-	4644	211.86501	1273.24676	-	-	-	-	-	-
-	-	-	4820	211.90172	2120.96072	-	-	-	-	-	-
-	-	-	5052	211.36591	1531.44081	-	-	-	-	-	-
-	-	-	5084	211.82741	4587.2	-	-	-	-	-	-
-	-	-	6004	211.90161	1778.79065	-	-	-	-	-	-
Mean	211.87158	-	Mean	211.78996	-	Mean	211.74931	-	Mean	211.78356	-
sd(yEr±)	0.14949	-	sd(yEr±)	0.15053	-	sd(yEr±)	0.15535	-	sd(yEr±)	0.16852	-
se(yEr±)	0.06103	-	se(yEr±)	0.03763	-	se(yEr±)	0.05493	-	se(yEr±)	0.05329	-
$\langle I \rangle$	-	3287.17267	$\langle I \rangle$	-	2272.86868	$\langle I \rangle$	2776.31249	-	$\langle I \rangle$	-	2543.21789
$\langle I \rangle^2$	-	10805504.16	$\langle I \rangle^2$	-	5165932.0365	$\langle I \rangle^2$	7707911.042	-	$\langle I \rangle^2$	-	6467957.236
$\langle I^2 \rangle$	-	$1.23553.10^7$	$\langle I^2 \rangle$	-	$6.46001.10^6$	$\langle I^2 \rangle$	$8.51381.10^6$	-	$\langle I^2 \rangle$	-	$7.79395.10^6$
$\langle I^2 \rangle – \langle I \rangle^2$	-	1549795.838	$\langle I^2 \rangle – \langle I \rangle^2$	-	1294077.9635	$\langle I^2 \rangle – \langle I \rangle^2$	805898.95787	-	$\langle I^2 \rangle – \langle I \rangle^2$	-	1325992.764
D''_{SD}	-	$4.089601.10^{-11}$	D''_{SD}	-	$3.414813.10^{-11}$	D''_{SD}	$2.126606.10^{-11}$	-	D''_{SD}	-	$3.49903.10^{-11}$
S5	c=30 ng.mL^{-1}		S6	c=50 ng.mL^{-1}		-	-	-	-	-	-
Scan	m/z	I [arb.units]	Scan	m/z	I [arb.units]	-	-	-	-	-	-
3126	211.62816	231.01099	3096	211.80154	1405.72396	-	-	-	-	-	-
3158	211.86586	4135.74613	3152	211.44	1053.24367	-	-	-	-	-	-
3166	211.46414	929.2939	3172	211.43669	566.65693	-	-	-	-	-	-
3493	211.80712	2465.94052	3172	211.70514	2319.04902	-	-	-	-		-
3525	211.90035	2797.18826	3192	211.73489	381.81405	-	-	-	-	-	-

4261	211.83485	4129.7862	3252	211.94716	2108.06922	-	-	-	-	-	-
4797	211.96867	2484.2284	3507	211.5908	4527.88224	-	-	-	-	-	-
5581	211.92762	2529.53833	3571	211.96894	4163.56856	-	-	-	-	-	-
5853	211.80063	1733.84488	3627	211.86805	1904.15508	-	-	-	-	-	-
5973	211.83503	2878.37038	3691	211.46165	1871.10694	-	-	-	-	-	-
6413	211.96747	1877.97292	4034	211.96825	1772.28683	-	-	-	-	-	-
-	-	-	4683	211.99816	4234.47762	-	-	-	-	-	-
-	-	-	5019	211.89306	3913.0211	-	-	-	-	-	-
-	-	-	5331	211.82187	2455.47502	-	-	-	-	-	-
-	-	-	5723	211.59323	2272.46947	-	-	-	-	-	-
Mean	211.81817	-	Mean	211.74863	-	-	-	-	-	-	-
sd(yEr±)	0.15112	-	sd(yEr±)	0.20139	-	-	-	-	-	-	-
se(yEr±)	0.04556	-	se(yEr±)	0.052	-	-	-	-	-	-	-
<I>	-	2381.17463	<I>	-	2329.93332	-	-	-	-	-	-
<I>^{2}	-	5669992.62	<I>^{2}	-	5428589.2757	-	-	-	-	-	-
<I^2>	-	$6.94269.10^{6}$	<I^2>	-	$7.06101.10^{6}$	-	-	-	-	-	-
<I^2>–<I>^{2}	-	1272697.38	<I^2>–<I>^{2}	-	1632420.72	-	-	-	-	-	-
D''_{SD}	-	$3.3584.10^{-11}$	D''_{SD}	-	$4.30763.10^{-11}$	-	-	-	-	-	-

Table S13

Theoretical average intensity data $\langle I \rangle^{Theor}$ of MS fragmentation ions of paracetamol at m/z 60 and 64 (see **Table S5**) according to equation (4); D''_{SD} [$cm^2.s^{-1}$] parameters of equation (2) with respect to collision energy CE [V]

m/z	60	64	60	64
CE [V]	$\langle I \rangle_{Theor}$	$\langle I \rangle_{Theor}$	D''_{SD}	D''_{SD}
10	$2.75.10^{6}$	$4.90.10^{-10}$	$2.34.10^{-6}$	$2.36.10^{-5}$
15	$1.68.10^{6}$	$1.20.10^{-9}$	$1.42.10^{-6}$	$5.79.10^{-5}$
20	351851.389	$3.91.10^{-10}$	$2.99.10^{-7}$	$1.89.10^{-5}$
25		$2.57.10^{-11}$		$1.24.10^{-6}$

Table S14

Temporal distribution of m/z and intensity data on fragmentation peaks of CID-MS/MS reaction of $[M+H]^+$ molecular cation of propranolol at m/z 260 of soil sample [101,102]; scan time[mins]; descriptive statistics; experimental measurands and parameters of equation (2)

Scan	t [mins]	m/z	I [arb.units]	m/z	I [arb.units]	m/z	I [arb.units]
1875	13.55	116.20028	3269.06797	157.1986	3578.22173	-	-
1890	14.01	-	-	157.01746	2387.38401	-	-
1910	14.1	116.29558	2547.98595	157.29036	2574.80596	259.43924	2374.02547
2067	15.22	-	-	-	-	259.0397	3384.53942
2077	15.26	-	-	-	-	-	-
2117	15.45	116.10027	3074.47523	157.15561	6768.88532	-	-
2127	15.5	116.22128	1940.15646	-	-	259.40107	4163.45252
2137	15.55	116.09684	7307.74891	157.17874	12278.3858	259.04003	8274.33711
2147	16	116.22343	8705.2987	157.01061	2499.62309	-	-
Mean	-	116.18961	-	157.1419	-	259.23001	-
sd(yEr±)	-	0.07756	-	0.10911	-	0.22011	-
se(yEr±)	-	0.03166	-	0.04454	-	0.11006	-
<I>	-	-	4474.1222	-	5014.55099	-	4549.08863
<I>^{2}	-	-	20017769.461	-	25145721.6313	-	20694207.364
<I^{2}>	-	-	2.65968.10^{7}	-	3.79929.10^{7}	-	2.57225.10^{7}
<I^{2}>–<I>^{2}	-	-	6579030.5395	-	12847178.3687	-	5028292.6364
**D"$_{SD}$**		**1.7360745787545941.10^{-10}**		**3.3901134279299224512.10^{-10}**		**1.3268658608944783316.10^{-10}**	

Table S15
Thermochemistry (M062X/SDD) of fragmentation species of antibiotics at ground (GS) and transition (TS) states in gas-phase.

	PARA (152_a)		d_3-PARA (155_a)		PARA (152_b)	
	GS	TS	GS	TS	GS	TS
E_{ZPVE}	109.24582	108.86651	103.10949	102.70620	109.43768	109.02682
E_{corr}	0.184670	0.183478	0.175204	0.173987	0.184777	0.183383
H_{corr}	0.185615	0.184422	0.176148	0.174931	0.185721	0.184327
G_{corr}	0.137694	0.138390	0.127352	0.128076	0.137464	0.137763
ε_0	0.174094	0.173490	0.164315	0.163673	0.174400	0.173745
E	-515.331713	-515.306320	-515.341179	-515.312997	-515.344726	-515.320379
H	-515.330768	-515.305376	-515.340235	-515.312053	-515.343781	-515.319434
G	-515.378689	-515.351407	-515.389031	-515.358909	-515.392038	-515.365998
	d_3-PARA (155_b)		PARA (325) $[2M+Na^+]$		PARA (174) $[M+Na^+]$	
	GS	TS	GS	TS	GS	TS
E_{ZPVE}	108.98043	103.32244	205.68077	205.41587	102.40145	101.92623
E_{corr}	0.183327	0.175358	0.350865	0.349642	0.175148	0.173746
H_{corr}	0.184271	0.176302	0.351809	0.350586	0.176093	0.174690
G_{corr}	0.137612	0.127174	0.273912	0.275713	0.123661	0.123526
ε_0	0.173671	0.164655	0.327773	0.327351	0.163187	0.162430
E	-515.317710	-515.354144	-1192.253403	-1192.244141	-677.155990	-677.133132
H	-515.316766	-515.353200	-1192.252458	-1192.243197	-677.155046	-677.132188
G	-515.363425	-515.402328	-1192.330355	-1192.318071	-677.207477	-677.183352
	d_3-PARA (177) $[M+Na^+]$		PARA (301) $[2M+H^+]$		PARA (158)	
	GS	TS	GS	TS	GS	TS
E_{ZPVE}	96.26044	95.91664	194.03973	194.03256	154.88567	154.07633
E_{corr}	0.165676	0.164402	0.331251	0.330361	0.258472	0.256795
H_{corr}	0.166620	0.165347	0.332195	0.331305	0.259416	0.257739
G_{corr}	0.113332	0.113863	0.251603	0.254162	0.208963	0.208209
ε_0	0.153401	0.152853	0.309222	0.309211	0.246826	0.245536
E	-677.165462	-677.149989	-1029.182657	-1029.183022	-518.900785	-518.890911
H	-677.164518	-677.149045	-1029.181713	-1029.182078	-518.899841	-518.889967
G	-677.217806	-677.200528	-1029.262305	-1029.259221	-518.950293	-518.939498
	d_3-PARA (161)		PARA (110)		PRO (260) $[M+H^+]$	
	GS	TS	GS	TS	GS	TS
E_{ZPVE}	148.71346	147.31251	86.48481	85.62887	228.88327	228.86407
E_{corr}	0.248994	0.246772	0.145067	0.136458	0.382742	0.381826
H_{corr}	0.249938	0.247716	0.146012	0.143850	0.383686	0.382770
G_{corr}	0.198644	0.195694	0.105718	0.144794	0.316054	0.318331
ε_0	0.236990	0.234757	0.137822	0.103509	0.364749	0.364718
E	-518.910262	-518.893925	-362.834578	-362.800486	-827.100705	-827.098312
H	-518.909318	-518.892981	-362.833634	-362.799542	-827.099761	-827.097368
G	-518.960612	-518.945003	-362.873927	-362.840828	-827.167393	-827.161807
	PRO (157)		PRO (116)			
	GS	TS	GS	TS		
E_{ZPVE}	107.45813	105.59760	125.25232	124.96038		
E_{corr}	0.180392	0.177537	0.211125	0.210103		
H_{corr}	0.181336	0.178481	0.212069	0.211047		
G_{corr}	0.136373	0.133746	0.160996	0.161432		
ε_0	0.171245	0.168280	0.199602	0.199137		
E	-499.041280	-498.950993	-366.340291	-366.341082		
H	-499.040336	-498.950049	-366.339347	-366.340137		
G	-499.085299	-498.994784	-366.390420	-366.389752		

E_{ZPVE} – zero-point vibrational energy [kcal.mol^{-1}]; ε_0 – zero-point correction [Hartree.(partice) $^{-1}$]; E_{corr} – thermal correction to energy [Hartree.(partice)$^{-1}$]; H_{corr} – thermal correction to enthalpy [Hartree.(partice)$^{-1}$]; G_{corr} – thermal correction to free energy [Hartree.(partice)$^{-1}$]; E – sum of electronic and thermal energies [Hartree.(partice)$^{-1}$]; H – sum of electronic and thermal enthalpies [Hartree.(partice)$^{-1}$]; G – sum of electronic and thermal free energies [Hartree.(partice)$^{-1}$]

Table S16

Atomic coordinates of most stable 3D molecular and electronic structures of fragmentation species of antibiotics at ground (GS) and transition (TS) states in gas-phase

d_3-PARA (155_a) (TS)				PARA (152_a) (TS)			
Atom/isotope	x	y	z	Atom/isotope	x	y	z
C	1.72219	-1.18908	0.02484	C	1.6364	-1.17672	0.02417
C	2.58794	-0.03109	-0.04371	C	2.50104	-0.02139	-0.04095
C	0.38869	-1.00462	0.06714	C	0.30063	-0.98997	0.06418
C	2.05515	1.3389	0.0903	C	1.97354	1.34633	0.0862
O	4.02124	-0.31838	0.00586	O	3.93584	-0.30678	0.00634
C	0.75275	1.47201	-0.00464	C	0.66737	1.48361	-0.00417
C	-0.15674	0.38605	-0.00456	C	-0.24214	0.39767	-0.00408
N	-1.52344	0.68803	-0.0078	N	-1.60884	0.69964	-0.00732
C	-2.59542	-0.21981	-0.00567	C	-2.68081	-0.2082	-0.00519
C	-3.95879	0.42934	0.01385	C	-4.04419	0.44095	0.01433
O	-2.41642	-1.44756	-0.00818	O	-2.50182	-1.43596	-0.0077
H	2.10007	-2.18821	0.00446	H	2.01465	-2.17657	0.00493
H	-0.35744	-1.77304	-0.00233	H	-0.44283	-1.7614	-0.00187
H	2.82893	2.08531	-0.0012	H	2.74355	2.09688	-0.00075
H	4.51049	-0.42688	-0.83541	H	4.42509	-0.41528	-0.83493
H	4.50662	-0.42312	0.84988	H	4.42122	-0.41151	0.85035
H	0.3808	2.49129	-0.00737	H	0.29542	2.5029	-0.00689
H	-1.77446	1.67129	-0.00813	H	-1.85986	1.68289	-0.00765
H(isotope=2)	-4.71436	-0.34094	-0.13408	H	-4.79976	-0.32933	-0.1336
H(isotope=2)	-4.05502	1.18276	-0.77417	H	-4.14042	1.19437	-0.77369
H(isotope=2)	-4.13582	0.91243	0.98065	H	-4.22122	0.92404	0.98113
d_3-PARA (155_a) (GS)				PARA (152_a) (GS)			
Atom/isotope	x	y	z	Atom/isotope	x	y	z
C	-1.638566	-1.18245	-0.001944	C	-1.638566	-1.18245	-0.001944
C	-2.481604	-0.077864	-0.002079	C	-2.481604	-0.077864	-0.002079
C	-0.259447	-0.952214	0.001571	C	-0.259447	-0.952214	0.001571
C	-2.055431	1.246459	0.0013	C	-2.055431	1.246459	0.0013
O	-3.945337	-0.326973	-0.005879	O	-3.945337	-0.326973	-0.005879
C	-0.676991	1.46339	0.004649	C	-0.676991	1.46339	0.004649
C	0.232663	0.377544	0.004568	C	0.232663	0.377544	0.004568
N	1.599345	0.67946	0.007792	N	1.599345	0.67946	0.007792
C	2.671306	-0.228382	0.005657	C	2.671306	-0.228382	0.005657
C	4.034681	0.420764	-0.013856	C	4.034681	0.420764	-0.013856
O	2.492314	-1.456143	0.008171	O	2.492314	-1.456143	0.008171
H	-2.023988	-2.196573	-0.004454	H	-2.023988	-2.196573	-0.004454
H	0.433336	-1.781378	0.002447	H	0.433336	-1.781378	0.002447
H	-2.753142	2.076394	0.00134	H	-2.753142	2.076394	0.00134
H	-4.434567	-0.435462	0.835415	H	-4.434567	-0.435462	0.835415
H	-4.430742	-0.431699	-0.84986	H	-4.430742	-0.431699	-0.84986
H	-0.305034	2.482723	0.007338	H	-0.305034	2.482723	0.007338
H	1.850366	1.662693	0.008119	H	1.850366	1.662693	0.008119
H(isotope=2)	4.790255	-0.349517	0.134074	H	4.790255	-0.349517	0.134074
H(isotope=2)	4.130914	1.174183	0.774162	H	4.130914	1.174183	0.774162
H(isotope=2)	4.211711	0.903854	-0.980661	H	4.211711	0.903854	-0.980661
d_3-PARA (155_b) (TS)				PARA (152_b) (TS)			
Atom/isotope	x	y	z	Atom/isotope	x	y	z
C	1.72219	-1.18908	0.02484	C	1.91274	1.17886	-0.11403
C	2.58794	-0.03109	-0.04371	C	2.45792	-0.03902	0.1343
C	0.38869	-1.00462	0.06714	C	0.62359	1.25789	-0.46222
C	2.05515	1.3389	0.0903	C	1.54791	-1.14535	0.18616
O	4.02124	-0.31838	0.00586	O	3.74632	-0.09994	0.55144

C	0.75275	1.47201	-0.00464	C	0.28314	-1.10662	-0.29264
C	-0.15674	0.38605	-0.00456	C	-0.21675	0.16178	-0.59883
N	-1.52344	0.68803	-0.0078	N	-1.65275	0.28079	-0.99994
C	-2.59542	-0.21981	-0.00567	C	-2.58683	-0.10481	0.19209
C	-3.95879	0.42934	0.01385	C	-2.6579	0.93203	1.26506
O	-2.41642	-1.44756	-0.00818	O	-3.10803	-1.19305	0.10078
H	2.10007	-2.18821	0.00446	H	2.56043	2.05321	-0.07143
H	-0.35744	-1.77304	-0.00233	H	0.17583	2.29533	-0.77858
H	2.82893	2.08531	-0.0012	H	2.0262	-2.20233	0.33611
H	4.51049	-0.42688	-0.83541	H	4.11595	-0.97961	0.75931
H	4.50662	-0.42312	0.84988	H	-0.3427	-1.9932	-0.35263
H	0.3808	2.49129	-0.00737	H	-1.87406	-0.37067	-1.77111
H	-1.77446	1.67129	-0.00813	H	-1.84714	1.24282	-1.31379
H(isotope=2)	-4.71436	-0.34094	-0.13408	H	-3.19444	0.52404	2.12152
H(isotope=2)	-4.05502	1.18276	-0.77417	H	-3.19753	1.81576	0.90353
H(isotope=2)	-4.13582	0.91243	0.98065	H	-1.65475	1.24084	1.57479
d_3-PARA (155_b) (GS)				PARA (152_b) (GS)			
Atom/isotope	x	y	z	Atom/isotope	x	y	z
C	-1.92515	1.171159	0.14446	C	-1.92515	1.171159	0.14446
C	-2.44867	-0.097392	-0.169127	C	-2.44867	-0.097392	-0.169127
C	-0.592678	1.293657	0.536119	C	-0.592678	1.293657	0.536119
C	-1.640081	-1.247227	-0.090433	C	-1.640081	-1.247227	-0.090433
O	-3.766057	-0.122104	-0.542788	O	-3.766057	-0.122104	-0.542788
C	-0.302944	-1.128793	0.301303	C	-0.302944	-1.128793	0.301303
C	0.197007	0.139633	0.607496	C	0.197007	0.139633	0.607496
N	1.633017	0.258625	1.008601	N	1.633017	0.258625	1.008601
C	2.567083	-0.126974	-0.183428	C	2.567083	-0.126974	-0.183428
C	2.638154	0.909864	-1.256401	C	2.638154	0.909864	-1.256401
O	3.088287	-1.21521	-0.092122	O	3.088287	-1.21521	-0.092122
H	-2.580252	2.031078	0.08008	H	-2.580252	2.031078	0.08008
H	-0.195178	2.273954	0.787554	H	-0.195178	2.273954	0.787554
H	-2.046332	-2.225322	-0.327462	H	-2.046332	-2.225322	-0.327462
H	-4.135736	-1.001762	-0.750673	H	-4.135736	-1.001762	-0.750673
H	0.32301	-2.015339	0.361289	H	0.32301	-2.015339	0.361289
H	1.854327	-0.392833	1.779772	H	1.854327	-0.392833	1.779772
H	1.827387	1.220646	1.322445	H	1.827387	1.220646	1.322445
H(isotope=2)	3.1747	0.501879	-2.112861	H	3.1747	0.501879	-2.112861
H(isotope=2)	3.177787	1.793601	-0.894874	H	3.177787	1.793601	-0.894874
H(isotope=2)	1.635009	1.218672	-1.566135	H	1.635009	1.218672	-1.566135
PARA (325) $[2M+Na^+]$ (GS)				PARA (325) $[2M+Na^+]$ (TS)			
Atom/isotope	x	y	z	Atom/isotope	x	y	z
C	-2.25507	1.05227	1.39328	C	2.25935	-1.0607	1.4249
C	-2.95235	1.36582	0.21849	C	2.9692	-1.34484	0.21235
C	-0.91363	1.42686	1.52014	C	0.94309	-1.4605	1.57697
C	-2.3328	2.09813	-0.80815	C	2.29088	-2.05197	-0.73875
O	-4.25444	0.90375	0.13415	O	4.25451	-0.90378	0.15318
C	-0.99513	2.48746	-0.66516	C	0.99518	-2.48749	-0.64612
C	-0.2742	2.12792	0.48603	C	0.27427	-2.12795	0.50507
N	1.11806	2.47033	0.59883	N	-1.11799	-2.47036	0.61786
C	2.05897	1.96236	-0.25163	C	-2.0589	-1.96239	-0.23259
C	3.49323	2.34985	-0.01638	C	-3.49317	-2.34988	0.00265
O	1.73159	1.17837	-1.19002	O	-1.73152	-1.1784	-1.17098
H	-2.77708	0.52865	2.18639	H	2.77717	-0.52867	2.20545
H	-0.36023	1.18534	2.42172	H	0.3603	-1.18538	2.4408
H	-2.88797	2.37054	-1.70134	H	2.88789	-2.37049	-1.6821
H	-4.75288	1.23634	-0.63648	H	4.75295	-1.23637	-0.61745

H	-0.50943	3.07686	-1.43708	H	0.50951	-3.07688	-1.41805
H	1.41385	3.09337	1.34226	H	-1.41379	-3.0934	1.36129
H	3.91956	2.72068	-0.95189	H	-3.9195	-2.72071	-0.93286
H	4.05236	1.45082	0.26732	H	-4.05229	-1.45085	0.28635
H	3.60597	3.11143	0.75821	H	-3.6059	-3.11146	0.77724
C	2.24788	-1.03556	1.38995	C	-2.24781	1.03553	1.40898
C	2.95182	-1.36888	0.22463	C	-2.95175	1.36885	0.24366
C	0.90565	-1.40774	1.51551	C	-0.90559	1.40771	1.53454
C	2.33731	-2.11688	-0.79387	C	-2.33725	2.11685	-0.77484
O	4.255	-0.91048	0.14136	O	-4.25494	0.91045	0.16039
C	0.99883	-2.50412	-0.65149	C	-0.99877	2.50409	-0.63246
C	0.27178	-2.12656	0.49014	C	-0.27171	2.12653	0.50917
N	-1.12072	-2.46786	0.60251	N	1.12079	2.46783	0.62154
C	-2.06076	-1.95543	-0.24657	C	2.06083	1.9554	-0.22753
C	-3.49514	-2.34432	-0.0157	C	3.49521	2.34429	0.00333
O	-1.73236	-1.16706	-1.1812	O	1.73243	1.16703	-1.16217
H	2.76544	-0.49905	2.17738	H	-2.76538	0.49901	2.19641
H	0.34773	-1.15269	2.41044	H	-0.34766	1.15266	2.42947
H	2.89701	-2.40364	-1.67971	H	-2.89695	2.40361	-1.66068
H	4.75796	-1.25532	-0.62084	H	-4.75789	1.25529	-0.60181
H	0.51709	-3.10522	-1.41695	H	-0.51702	3.10519	-1.39792
H	-1.41699	-3.09277	1.34415	H	1.41706	3.09274	1.36318
H	-4.05507	-1.44539	0.26638	H	4.05513	1.44536	0.28542
H	-3.61022	-3.1059	0.75848	H	3.61028	3.10587	0.77751
H	-3.91819	-2.7149	-0.95275	H	3.91825	2.71487	-0.93371
Na	0.01029	-0.01094	-1.71951	Na	-0.01022	0.01091	-1.70048
PARA (174) [M+Na$^+$] (GS)				PARA (174) [M+Na$^+$] (TS)			
C	1.78945	0.31743	1.14993	C	1.76529	0.26078	1.17784
C	2.55445	-0.15256	0.06308	C	2.54967	-0.16374	0.03267
C	0.43576	-0.02376	1.24166	C	0.45047	-0.11936	1.30849
C	1.96215	-0.95106	-0.93041	C	1.86665	-0.92339	-0.84421
O	3.87745	0.22657	0.04895	O	3.84678	0.18291	0.05739
C	0.59755	-1.26989	-0.84214	C	0.56679	-1.31354	-0.83367
C	-0.1665	-0.80611	0.23672	C	-0.19719	-0.84977	0.24519
N	-1.58146	-1.06861	0.29746	N	-1.61215	-1.11227	0.30591
C	-2.47555	-0.08321	-0.00104	C	-2.50624	-0.12688	0.0074
C	-3.94198	-0.40058	0.08484	C	-3.97267	-0.44425	0.09328
O	-2.06238	1.06588	-0.34481	O	-2.09308	1.02222	-0.33637
H	2.28964	0.88839	1.92399	H	2.25901	0.84476	1.93254
H	-0.15337	0.29172	2.09807	H	-0.18402	0.24801	2.10663
H	2.55364	-1.32767	-1.75972	H	2.52242	-1.37096	-1.75056
H	4.43095	-0.20578	-0.6296	H	4.4003	-0.24942	-0.62117
H	0.12965	-1.8762	-1.61075	H	0.09903	-1.91986	-1.60231
H	-1.91314	-1.98868	0.56685	H	-1.94384	-2.03235	0.5753
H	-4.39697	-0.24935	-0.89755	H	-4.42766	-0.29302	-0.8891
H	-4.41843	0.30066	0.77465	H	-4.44912	0.257	0.78309
H	-4.13524	-1.42191	0.41811	H	-4.16593	-1.46557	0.42655
Na	-0.21536	2.08617	-0.60138	Na	-0.24606	2.04251	-0.59294
d_3-PARA (177) [M+Na$^+$] (GS)				d_3-PARA (177) [M+Na$^+$] (TS)			
C	1.78945	0.317432	1.149933	C	1.83824	0.27161	1.17259
C	2.554448	-0.152562	0.063083	C	2.61923	-0.16094	0.03768
C	0.435756	-0.023758	1.241655	C	0.51659	-0.10166	1.29638
C	1.962145	-0.951062	-0.930406	C	1.95217	-0.92742	-0.85971
O	3.877453	0.226568	0.048949	O	3.92089	0.19144	0.05557
C	0.597549	-1.269885	-0.842144	C	0.64094	-1.30501	-0.8355
C	-0.166501	-0.806105	0.236723	C	-0.12307	-0.84124	0.24336

N	-1.58146	-1.06861	0.297461	N	-1.53803	-1.10374	0.30409
C	-2.475547	-0.083213	-0.001044	C	-2.43212	-0.11835	0.00558
C	-3.941977	-0.400584	0.084839	C	-3.89855	-0.43572	0.09146
O	-2.062385	1.06588	-0.344814	O	-2.01896	1.03075	-0.33819
H	2.28964	0.888388	1.923994	H	2.3331	0.85328	1.93067
H	-0.153366	0.291722	2.098073	H	-0.10992	0.25657	2.10476
H	2.553641	-1.327669	-1.759716	H	2.59676	-1.36259	-1.75268
H	4.430952	-0.205775	-0.629602	H	4.4744	-0.2409	-0.62299
H	0.129649	-1.876203	-1.610746	H	0.17312	-1.91133	-1.60413
H	-1.913142	-1.988681	0.566851	H	-1.86972	-2.02381	0.57348
H(isotope=2)	-4.396968	-0.24935	-0.897548	H(isotope=2)	-4.35354	-0.28448	-0.89092
H(isotope=2)	-4.418432	0.300662	0.774646	H(isotope=2)	-4.375	0.26553	0.78127
H(isotope=2)	-4.135241	-1.421907	0.418108	H(isotope=2)	-4.09181	-1.45704	0.42473
Na	-0.215364	2.086174	-0.601381	Na	-0.17194	2.05104	-0.59476
PARA (301) [2M+H$^+$] (GS)				PARA (301) [2M+H$^+$] (TS)			
C	-4.442217	0.918088	0.862735	C	4.41355	-0.90128	0.87963
C	-5.068107	1.269061	-0.444707	C	5.04173	-1.24691	-0.43468
C	-3.43103	0.028014	0.937827	C	3.40695	-0.01579	0.95931
C	-4.542342	0.593162	-1.665412	C	4.50528	-0.56643	-1.64164
O	-5.995662	2.092937	-0.517005	O	5.96623	-2.0746	-0.5024
C	-3.533639	-0.29907	-1.59126	C	3.50421	0.3174	-1.57665
C	-2.918378	-0.628143	-0.288541	C	2.88895	0.64648	-0.27393
N	-1.961321	-1.480483	-0.239516	N	1.93189	1.49882	-0.22491
C	-1.008367	-2.308861	0.111638	C	0.97894	2.32719	0.12624
C	-1.339278	-3.759995	0.352966	C	1.30985	3.77833	0.36757
O	0.239941	-1.950108	0.207416	O	-0.26937	1.96844	0.22202
H	-4.837746	1.428837	1.734259	H	4.80832	-1.4105	1.74887
H	-2.944891	-0.218321	1.875222	H	2.91546	0.23665	1.88983
H	-5.019215	0.858573	-2.603377	H	4.98979	-0.84024	-2.58877
H	-3.142165	-0.810445	-2.463739	H	3.11274	0.82878	-2.44913
H	-0.812144	-4.095707	1.249205	H	0.78272	4.11404	1.26381
H	-0.947148	-4.346169	-0.480754	H	0.91772	4.3645	-0.46615
H	-2.412771	-3.93471	0.445952	H	2.38334	3.95304	0.46056
C	4.633224	-0.826912	-0.308181	C	-4.66265	0.84525	-0.29357
C	5.299583	0.375384	-0.587005	C	-5.32901	-0.35705	-0.5724
C	3.247592	-0.831807	-0.135279	C	-3.27702	0.85014	-0.12067
C	4.584885	1.580646	-0.700333	C	-4.61431	-1.56231	-0.68573
O	6.665006	0.287631	-0.733231	O	-6.69444	-0.2693	-0.71862
C	3.196413	1.574881	-0.528352	C	-3.22584	-1.55655	-0.51375
C	2.541284	0.372454	-0.242674	C	-2.57071	-0.35412	-0.22807
N	1.085587	0.360885	-0.041309	N	-1.11502	-0.34255	-0.0267
C	0.534098	0.946192	1.217114	C	-0.56353	-0.92786	1.23172
C	1.431582	0.96243	2.415639	C	-1.46101	-0.9441	2.43025
O	-0.647936	1.272098	1.179976	O	0.61851	-1.25376	1.19458
H	5.213354	-1.737633	-0.231605	H	-5.24278	1.75597	-0.217
H	2.727614	-1.763576	0.070302	H	-2.75704	1.78191	0.08491
H	5.099873	2.512435	-0.912583	H	-5.1293	-2.4941	-0.89798
H	7.116295	1.126432	-0.950431	H	-7.14572	-1.1081	-0.93582
H	2.642712	2.506419	-0.61138	H	-2.67214	-2.48809	-0.59677
H	0.674115	-0.783042	0.012607	H	-0.70354	0.80138	0.02721
H	0.588932	0.790368	-0.834401	H	-0.61836	-0.77203	-0.81979
H	2.270157	1.646256	2.261788	H	-2.29959	-1.62792	2.27639
H	1.851915	-0.034557	2.582936	H	-1.88134	0.05289	2.59754
H	0.858644	1.278416	3.28749	H	-0.88807	-1.26008	3.3021
PARA (158) (GS)				PARA (158) (TS)			
C	-0.911941	-1.439449	-0.377831	C	0.85052	1.45196	-0.35019

C	-2.333286	-1.252072	0.196459	C	2.28446	1.29397	0.18632
C	0.054331	-0.431157	0.262653	C	-0.09056	0.41847	0.31549
N	1.454974	-0.665648	-0.316982	N	-1.55946	0.7293	-0.25529
C	-0.387708	1.018235	0.016621	C	0.32209	-0.99734	0.03167
C	-1.800086	1.237883	0.601332	C	1.73447	-1.21698	0.61638
C	-2.781669	0.199061	0.07754	C	2.71605	-0.17815	0.09258
O	-3.871961	0.509948	-0.411949	O	3.80634	-0.48904	-0.3969
C	2.561788	0.199561	0.275945	C	-2.62741	-0.17865	0.29099
C	3.868895	-0.573726	0.362443	C	-3.93452	0.59463	0.37749
O	2.587506	1.271605	-0.672343	O	-2.65313	-1.2507	-0.6573
H	-0.939264	-1.280394	-1.464734	H	0.87363	1.30129	-1.44965
H	-0.582425	-2.470408	-0.196924	H	0.5168	2.49128	-0.18187
H	-3.048645	-1.88774	-0.32878	H	2.98311	1.90873	-0.31381
H	-2.346477	-1.535696	1.257513	H	2.28087	1.5566	1.27249
H	0.141085	-0.626214	1.339335	H	-0.20669	0.64711	1.3545
H	1.440852	-0.422119	-1.323285	H	-1.50649	0.4431	-1.30801
H	1.694047	-1.662658	-0.224412	H	-1.75974	1.68383	-0.20931
H	0.312093	1.730818	0.461852	H	-0.37771	-1.70992	0.47689
H	-0.397874	1.217427	-1.063469	H	0.33225	-1.19651	-1.04842
H	-2.169662	2.232873	0.346028	H	2.10404	-2.21196	0.36107
H	-1.766749	1.163312	1.696989	H	1.70113	-1.14241	1.71203
H	2.215083	0.535342	1.25926	H	-2.28072	-0.51445	1.27431
H	4.65513	0.076939	0.756111	H	-4.72076	-0.05603	0.77116
H	4.182495	-0.920602	-0.627196	H	-4.24811	0.94151	-0.61215
H	3.785981	-1.426539	1.043884	H	-3.8516	1.44744	1.05893
H	3.293212	1.932758	-0.529929	H	-3.35883	-1.91185	-0.51488
d_3-PARA (161) (GS)				d_3-PARA (161) (TS)			
C	-0.911941	-1.439449	-0.377831	C	0.85166	1.44967	-0.34676
C	-2.333286	-1.252072	0.196459	C	2.28903	1.29969	0.17946
C	0.054331	-0.431157	0.262653	C	-0.08255	0.40933	0.32579
N	1.454974	-0.665648	-0.316982	N	-1.57005	0.74093	-0.24259
C	-0.387708	1.018235	0.016621	C	0.32209	-0.99734	0.03167
C	-1.800086	1.237883	0.601332	C	1.73447	-1.21698	0.61638
C	-2.781669	0.199061	0.07754	C	2.71605	-0.17815	0.09258
O	-3.871961	0.509948	-0.411949	O	3.80634	-0.48904	-0.3969
C	2.561788	0.199561	0.275945	C	-2.62742	-0.17865	0.291
C	3.868895	-0.573726	0.362443	C	-3.93452	0.59463	0.37749
O	2.587506	1.271605	-0.672343	O	-2.65312	-1.2507	-0.6573
H	-0.939264	-1.280394	-1.464734	H	0.87361	1.30128	-1.4496
H	-0.582425	-2.470408	-0.196924	H	0.5168	2.49125	-0.18186
H	-3.048645	-1.88774	-0.32878	H	2.98321	1.90882	-0.31389
H	-2.346477	-1.535696	1.257513	H	2.28089	1.5566	1.27242
H	0.141085	-0.626214	1.339335	H	-0.20667	0.6471	1.35463
H	1.440852	-0.422119	-1.323285	H	-1.50651	0.44319	-1.30778
H	1.694047	-1.662658	-0.224412	H	-1.75981	1.68413	-0.20925
H	0.312093	1.730818	0.461852	H	-0.3777	-1.70993	0.47689
H	-0.397874	1.217427	-1.063469	H	0.33225	-1.1965	-1.04842
H	-2.169662	2.232873	0.346028	H	2.10404	-2.21196	0.36107
H	-1.766749	1.163312	1.696989	H	1.70113	-1.14241	1.71203
H	2.215083	0.535342	1.25926	H	-2.28073	-0.51446	1.27431
H	4.65513	0.076939	0.756111	H(isotope=2)	-4.72076	-0.05603	0.77115
H	4.182495	-0.920602	-0.627196	H(isotope=2)	-4.24811	0.94151	-0.61215
H	3.785981	-1.426539	1.043884	H(isotope=2)	-3.8516	1.44744	1.05893
H	3.293212	1.932758	-0.529929	H	-3.35883	-1.91185	-0.51488
PARA (110) (GS)				PARA (110) (TS)			
C	-0.767153	1.224586	-0.000263	C	-0.7061	1.20034	0.02609

C	-1.46767	0.004642	0.000259	C	-1.38018	0.04143	-0.05238
O	-2.834331	0.100712	0.001205	O	-2.72868	0.03305	0.06968
C	0.627454	1.22289	-0.001045	C	0.59233	1.31237	0.10432
C	-0.774437	-1.220666	-0.00018	C	-0.72202	-1.22749	-0.00019
C	0.623951	-1.225983	-0.001037	C	0.67638	-1.23285	-0.00103
C	1.298546	-0.004107	-0.001531	C	1.35087	-0.01085	-0.00149
N	2.799021	-0.007194	0.001683	N	2.85143	-0.01407	0.0017
H	-1.334143	2.147019	-0.00057	H	-1.28172	2.1398	-0.00049
H	-3.322463	-0.74506	-0.000352	H	-3.27004	-0.75252	-0.00039
H	1.163229	2.168571	-0.001363	H	1.21565	2.16245	-0.00115
H	-1.316122	-2.160998	-0.00025	H	-1.26378	-2.1678	-0.00022
H	1.156429	-2.173016	-0.001279	H	1.20887	-2.1799	-0.00127
H	3.151981	0.95896	-0.04621	H	3.20441	0.95207	-0.0462
H	3.170465	-0.520929	-0.810291	H	3.22287	-0.5278	-0.81028
H	3.167981	-0.438062	0.861675	H	3.22041	-0.44494	0.86169
PRO (260) (GS)				PRO (260) (TS)			
C	4.040505	-2.252165	-0.112289	C	3.92279	-2.26626	-0.12175
C	5.085679	-1.501984	-0.727632	C	4.97483	-1.50004	-0.75771
C	2.905334	-1.617644	0.355515	C	2.80136	-1.64548	0.35981
C	4.981209	-0.129593	-0.847094	C	4.83829	-0.1139	-0.83593
C	2.769216	-0.203341	0.222364	C	2.6492	-0.21286	0.20604
C	3.823884	0.55684	-0.371138	C	3.70387	0.54733	-0.38747
C	1.617112	0.504031	0.679705	C	1.4971	0.49452	0.66338
C	3.705402	1.978657	-0.454743	C	3.58539	1.96915	-0.47107
C	1.518108	1.875679	0.606228	C	1.3981	1.86617	0.5899
O	0.555595	-0.242443	1.257565	O	0.43558	-0.25196	1.24124
C	2.587164	2.625505	0.033324	C	2.46715	2.61599	0.01699
C	-0.322887	-0.912223	0.309971	C	-0.4429	-0.92174	0.29364
C	-1.450318	0.073477	-0.005297	C	-1.57033	0.06396	-0.02163
C	-2.637542	-0.568712	-0.730407	C	-2.75755	-0.57822	-0.74674
O	-2.024448	0.554227	1.239704	O	-2.14446	0.54471	1.22337
N	-3.837325	0.330091	-0.518492	N	-3.95734	0.32058	-0.53482
C	-5.202654	-0.353115	-0.666934	C	-5.32267	-0.36263	-0.68326
C	-6.259259	0.733537	-0.882437	C	-6.37927	0.72402	-0.89877
C	-5.479996	-1.193965	0.582123	C	-5.60001	-1.20348	0.56579
H	4.150869	-3.325345	0.00015	H	4.03086	-3.33486	-0.01618
H	5.970105	-2.01562	-1.087759	H	5.8501	-2.02513	-1.10409
H	2.126751	-2.179147	0.862757	H	2.00674	-2.18867	0.84643
H	5.783138	0.446889	-1.298563	H	5.66311	0.43738	-1.31488
H	4.518179	2.545568	-0.898423	H	4.39817	2.53606	-0.91475
H	0.641155	2.379139	1.003266	H	0.52114	2.36963	0.98694
H	2.512628	3.705955	-0.014577	H	2.39262	3.69644	-0.03091
H	-0.716545	-1.79168	0.828529	H	-0.83656	-1.80119	0.8122
H	0.21971	-1.217583	-0.591901	H	0.0997	-1.2271	-0.60823
H	-1.04451	0.922861	-0.571702	H	-1.16452	0.91335	-0.58803
H	-2.876221	-1.533864	-0.277806	H	-2.99623	-1.54338	-0.29414
H	-2.466113	-0.698349	-1.800463	H	-2.58613	-0.70786	-1.81679
H	-1.328792	0.654724	1.925587	H	-1.4488	0.64521	1.90926
H	-3.789516	1.131103	-1.161763	H	-3.90953	1.12159	-1.17809
H	-3.719101	0.695272	0.451561	H	-3.83911	0.68576	0.43523
H	-5.122307	-0.989601	-1.554216	H	-5.24232	-0.99911	-1.57055
H	-7.240679	0.267586	-0.997781	H	-7.36069	0.25807	-1.01411
H	-6.067553	1.323278	-1.785828	H	-6.18757	1.31377	-1.80216
H	-6.31271	1.407481	-0.020039	H	-6.43272	1.39797	-0.03637
H	-6.43747	-1.706111	0.459023	H	-6.55748	-1.71562	0.44269
H	-5.553882	-0.557415	1.471133	H	-5.67389	-0.56693	1.4548

H	-4.720782	-1.959949	0.762548	H	-4.84079	-1.96946	0.74622
PRO (116) (GS)				PRO (116) (TS)			
C	2.68532	0.48452	0.90871	C	2.65311	-0.50382	-0.9153
C	2.70914	0.13631	-0.37872	C	2.67922	-0.15026	0.36526
O	1.51118	0.12098	-1.14619	O	1.48284	-0.14405	1.14326
C	-0.56016	-2.03045	0.54205	C	-0.60078	2.02109	-0.54176
N	-0.63937	-0.8313	0.08815	N	-0.67234	0.81353	-0.09703
C	-1.80507	0.08774	0.25359	C	-1.83804	-0.10551	-0.26248
C	-1.34158	1.30509	1.06708	C	-1.37456	-1.32286	-1.07596
C	-2.31184	0.47928	-1.14154	C	-2.34481	-0.49705	1.13266
H	3.5952	0.44317	1.4939	H	3.56222	-0.46094	-1.50278
H	1.78213	0.83759	1.39709	H	1.74916	-0.85536	-1.40597
H	3.59414	-0.19163	-0.91352	H	3.56116	0.17386	0.90464
H	1.64509	0.33127	-2.0893	H	1.61211	-0.34904	2.08042
H	-1.38613	-2.46813	1.09678	H	-1.41911	2.45036	-1.10566
H	0.34115	-2.61117	0.37043	H	0.30817	2.5934	-0.37931
H	0.18315	-0.42295	-0.44901	H	0.15018	0.40518	0.44012
H	-2.57735	-0.46525	0.79866	H	-2.61033	0.44748	-0.80754
H	-2.182	1.99221	1.19361	H	-2.21498	-2.00998	-1.20249
H	-0.54461	1.84248	0.54028	H	-0.57758	-1.86025	-0.54916
H	-0.98454	1.01682	2.06012	H	-1.01752	-1.03459	-2.069
H	-3.17855	1.13648	-1.035	H	-3.21152	-1.15425	1.02612
H	-2.61469	-0.39444	-1.72491	H	-2.64766	0.37667	1.71603
H	-1.54175	1.02984	-1.69364	H	-1.57473	-1.04761	1.68475
PRO (157) (GS)				PRO (157) (TS)			
C	2.121744	-1.76189	-0.006066	C	2.131	-1.78667	0.03666
C	2.985269	-0.627686	0.02045	C	3.03438	-0.55633	-0.0602
C	0.746	-1.610629	-0.022593	C	0.83643	-1.71753	0.10254
C	2.456875	0.647421	0.028088	C	2.31547	0.80012	0.1943
C	0.184279	-0.304507	-0.017455	C	0.17939	-0.30244	-0.01584
C	1.043113	0.843406	0.009245	C	1.03737	0.84598	0.01087
C	-1.213196	-0.031256	-0.03005	C	-1.21822	-0.02885	-0.02852
C	0.474288	2.146929	0.014338	C	0.46908	2.1494	0.01586
C	-1.77531	1.235159	-0.034949	C	-1.78041	1.23759	-0.03341
O	-2.029007	-1.199337	-0.057203	O	-2.03411	-1.19692	-0.05566
C	-0.902857	2.344432	-0.010184	C	-0.90793	2.34687	-0.00865
C	-3.282299	-1.347332	0.084762	C	-3.28738	-1.3449	0.0863
H	2.552928	-2.756647	-0.013598	H	2.54905	-2.75539	-0.01204
H	4.058536	-0.775618	0.034082	H	4.05558	-0.7725	0.03515
H	0.101621	-2.481361	-0.042391	H	0.09662	-2.48038	-0.0402
H	3.106244	1.516726	0.048287	H	3.10067	1.52131	0.05067
H	1.139016	3.005056	0.030915	H	1.13393	3.00744	0.03253
H	-2.846684	1.405251	-0.077863	H	-2.85178	1.40767	-0.07633
H	-1.31489	3.345474	-0.019447	H	-1.32001	3.34791	-0.0179
H	-3.649953	-2.365816	0.005375	H	-3.65505	-2.3634	0.00691
H	-3.942209	-0.502644	0.278747	H	-3.9473	-0.50021	0.28029

Table S17
Molecular vibrations (M062X/SDD) of most stable 3D molecular and electronic structures of fragmentation species of antibiotics at ground (GS) and transition (TS) states in gas-phase

d_3-PARA (155_a) (TS)				d_3-PARA (155_a) (GS)			
Frequencies	-60.5606	71.186	104.6795	Frequencies	55.9422	64.7544	76.4244
Frequencies	150.821	165.1937	259.5624	Frequencies	162.0758	175.3064	259.3632
Frequencies	283.5685	291.5234	307.9536	Frequencies	294.6444	314.2592	341.2638
Frequencies	345.8769	372.4588	478.3	Frequencies	395.5409	441.5263	469.2929
Frequencies	486.1847	497.8673	539.8529	Frequencies	478.2433	509.8287	550.7919
Frequencies	600.5851	625.6437	686.5815	Frequencies	604.408	644.5073	683.2731
Frequencies	721.4921	730.9768	771.7569	Frequencies	728.7705	751.5768	812.4394
Frequencies	825.8792	862.3252	867.5409	Frequencies	863.0397	889.3441	894.5148
Frequencies	928.3598	941.2096	945.0585	Frequencies	940.9835	941.7533	1003.7505
Frequencies	1009.5317	1022.7755	1052.6537	Frequencies	1022.6544	1042.9818	1058.5626
Frequencies	1082.8205	1097.8811	1123.78	Frequencies	1083.2291	1098.0538	1123.0201
Frequencies	1126.8175	1129.6618	1227.7613	Frequencies	1143.3895	1167.8915	1228.2818
Frequencies	1269.4913	1275.7636	1344.3724	Frequencies	1274.8592	1320.957	1355.0446
Frequencies	1348.8096	1452.9137	1507.2749	Frequencies	1393.7897	1455.0911	1549.7392
Frequencies	1572.7942	1663.6167	1702.505	Frequencies	1571.0428	1672.4467	1682.9392
Frequencies	1747.9316	1869.1958	2224.1423	Frequencies	1696.2946	1752.0842	2224.216
Frequencies	2348.2735	2395.7102	3227.1156	Frequencies	2348.3282	2395.4496	3235.215
Frequencies	3309.9889	3345.9755	3412.3909	Frequencies	3243.6364	3252.7895	3277.4722
Frequencies	3647.6841	3657.115	3784.8431	Frequencies	3647.9643	3663.7456	3797.3494
PARA (152_a) (TS)				PARA (152_a) (GS)			
Frequencies	-43.7129	95.7733	104.3457	Frequencies	64.7556	66.9729	96.7276
Frequencies	159.488	168.5072	264.9283	Frequencies	169.5437	178.5734	259.3633
Frequencies	288.4285	299.1666	322.9575	Frequencies	307.9933	323.9773	343.2053
Frequencies	350.1757	376.9992	484.5074	Frequencies	396.3304	441.6965	469.3218
Frequencies	503.0114	504.15	603.8498	Frequencies	502.234	522.2055	606.4647
Frequencies	616.5632	640.4872	689.9809	Frequencies	628.583	650.2951	687.0491
Frequencies	725.2694	738.9712	796.2802	Frequencies	729.2199	762.1117	856.4587
Frequencies	828.3531	867.8086	933.4819	Frequencies	863.0912	890.1068	971.2712
Frequencies	975.1549	1009.4807	1022.7103	Frequencies	1003.6573	1021.8191	1036.14
Frequencies	1031.9046	1052.7535	1095.0406	Frequencies	1042.9674	1058.561	1094.9284
Frequencies	1129.379	1132.3886	1229.8681	Frequencies	1143.3515	1167.7509	1228.5525
Frequencies	1270.1409	1283.3402	1344.03	Frequencies	1282.454	1320.7163	1355.2235
Frequencies	1350.3138	1450.5858	1459.8395	Frequencies	1393.7755	1450.674	1454.0921
Frequencies	1495.2629	1514.7397	1524.2028	Frequencies	1502.2673	1524.1916	1550.4321
Frequencies	1576.4634	1655.4317	1702.2126	Frequencies	1574.4416	1672.5461	1683.1062
Frequencies	1752.714	1856.7644	3089.9681	Frequencies	1696.2957	1756.8928	3090.0474
Frequencies	3173.5907	3227.7432	3234.5799	Frequencies	3173.6584	3234.2829	3235.2252
Frequencies	3310.4843	3343.7538	3404.1386	Frequencies	3243.6365	3252.7896	3277.4725
Frequencies	3647.7299	3657.5328	3785.5074	Frequencies	3647.966	3663.7456	3797.3494
d_3-PARA (155_b) (TS)				d_3-PARA (155_b) (GS)			
Frequencies	-72.6618	35.9956	59.2275	Frequencies	39.3961	50.7053	81.1661
Frequencies	136.251	170.4751	274.9884	Frequencies	102.2004	173.0691	280.0168
Frequencies	302.1076	353.0903	386.5158	Frequencies	326.6901	377.7614	405.6238
Frequencies	405.7376	422.1534	453.5219	Frequencies	422.7142	427.9515	431.9935
Frequencies	518.9606	546.7885	592.8727	Frequencies	497.642	520.4601	556.1142
Frequencies	652.1461	710.041	743.9295	Frequencies	649.8602	683.116	743.2778
Frequencies	770.2073	881.4438	894.8128	Frequencies	759.4492	807.5234	856.5069
Frequencies	947.9653	987.3789	1019.8318	Frequencies	860.0705	877.7769	934.0428
Frequencies	1050.4273	1086.1968	1150.5377	Frequencies	975.5203	1001.4526	1015.9626
Frequencies	1155.8443	1176.6836	1189.3575	Frequencies	1031.4109	1052.2267	1075.0615
Frequencies	1225.5512	1231.9943	1281.6083	Frequencies	1084.4803	1138.5648	1163.4204
Frequencies	1306.9024	1379.7189	1387.4545	Frequencies	1176.5734	1213.8406	1220.699

Frequencies	1448.8742	1461.5217	1482.2471	Frequencies	1244.7833	1337.9859	1369.0965
Frequencies	1490.4021	1505.9574	1602.5816	Frequencies	1384.7479	1413.9757	1502.9375
Frequencies	1668.7506	1680.4167	1767.4317	Frequencies	1569.689	1671.9543	1685.8379
Frequencies	1868.7913	1894.3709	2443.2073	Frequencies	1702.9152	1866.2696	2212.1271
Frequencies	2502.3386	3073.982	3163.9956	Frequencies	2339.8335	2380.9083	3206.6457
Frequencies	3212.5608	3213.5755	3230.702	Frequencies	3222.8869	3238.9389	3273.3471
Frequencies	3384.5973	3480.0519	3767.8481	Frequencies	3385.3732	3481.7681	3768.7329
PARA (152_b) (TS)				PARA (152_b) (GS)			
Frequencies	-67.879	37.8085	60.8696	Frequencies	41.3378	54.1548	83.085
Frequencies	136.45	171.0891	275.6107	Frequencies	139.7584	178.1542	284.206
Frequencies	304.8499	358.3596	386.7714	Frequencies	341.3382	390.8344	405.6264
Frequencies	407.1372	421.5916	453.3071	Frequencies	426.7127	428.0519	448.19
Frequencies	519.1003	546.7783	592.9336	Frequencies	518.0183	534.511	587.7036
Frequencies	652.4038	710.2631	744.5826	Frequencies	651.6252	707.1816	746.2271
Frequencies	770.2199	880.9301	894.862	Frequencies	768.3602	857.5647	860.133
Frequencies	946.7951	984.3946	1021.6005	Frequencies	881.0481	896.8996	1000.5222
Frequencies	1050.5141	1086.2274	1150.641	Frequencies	1015.9333	1032.7322	1055.0168
Frequencies	1155.5204	1171.1894	1182.9822	Frequencies	1086.7769	1139.3753	1154.5607
Frequencies	1224.4047	1231.8145	1279.6624	Frequencies	1174.9783	1215.3345	1221.0263
Frequencies	1304.0837	1379.6629	1386.7159	Frequencies	1242.7371	1337.9828	1368.838
Frequencies	1448.8989	1458.0999	1480.6292	Frequencies	1383.6989	1413.7043	1449.4849
Frequencies	1490.4295	1505.9825	1600.3124	Frequencies	1490.9221	1501.8986	1507.3972
Frequencies	1668.1589	1674.8481	1762.5184	Frequencies	1569.7341	1672.185	1685.8525
Frequencies	1868.8367	1884.3443	2474.1068	Frequencies	1702.9288	1869.7935	3074.2968
Frequencies	2535.6397	3073.9942	3163.9906	Frequencies	3163.9118	3206.644	3212.7293
Frequencies	3212.5546	3216.6352	3230.6484	Frequencies	3222.887	3238.9389	3273.3472
Frequencies	3384.6352	3480.1461	3767.8308	Frequencies	3385.3763	3481.7692	3768.7329
PARA (325) [2M+Na^+] (GS)				PARA (325) [2M+Na^+] (TS)			
Frequencies	7.8975	42.0739	44.5948	Frequencies	-23.1353	25.1304	38.1024
Frequencies	69.5129	70.2826	82.6642	Frequencies	61.5289	67.8471	82.0009
Frequencies	94.6909	100.0502	104.774	Frequencies	87.7284	99.2617	102.9578
Frequencies	116.2081	117.4259	132.6211	Frequencies	111.0723	116.0786	132.2832
Frequencies	139.349	171.5566	172.4555	Frequencies	137.4479	171.2075	171.6974
Frequencies	181.8953	215.2551	216.1959	Frequencies	181.66	209.9732	215.379
Frequencies	293.8253	344.2439	351.6359	Frequencies	294.0477	341.765	347.0276
Frequencies	354.1939	373.4749	375.7726	Frequencies	353.9872	369.8617	375.8427
Frequencies	386.3225	393.2129	397.6286	Frequencies	387.392	398.3233	405.1275
Frequencies	434.0036	442.4996	449.5297	Frequencies	415.0311	436.6319	438.6936
Frequencies	450.3439	513.3896	519.7325	Frequencies	450.0338	514.5235	521.0185
Frequencies	544.6681	550.5345	619.7039	Frequencies	540.6629	548.6253	618.8254
Frequencies	620.3324	635.2587	635.683	Frequencies	621.8651	635.2392	635.7385
Frequencies	642.3687	642.5532	665.7512	Frequencies	642.2813	642.9558	665.1964
Frequencies	666.2199	746.9957	749.6539	Frequencies	666.0106	746.3077	749.6023
Frequencies	817.8435	820.274	870.2857	Frequencies	818.4074	825.232	865.0898
Frequencies	871.4966	881.6245	885.4791	Frequencies	870.2971	883.0314	893.4828
Frequencies	906.1737	915.7534	984.8484	Frequencies	910.5351	938.6934	984.9345
Frequencies	985.9154	1015.6637	1018.1889	Frequencies	996.3754	1014.0792	1016.029
Frequencies	1030.2226	1037.9042	1044.3784	Frequencies	1030.2194	1036.8833	1046.9753
Frequencies	1051.0868	1063.0382	1063.8515	Frequencies	1062.9714	1063.5267	1108.0654
Frequencies	1108.4363	1108.8981	1139.5739	Frequencies	1108.8586	1138.7091	1140.5408
Frequencies	1141.272	1176.2175	1176.7146	Frequencies	1150.5946	1176.2785	1178.4175
Frequencies	1215.4014	1216.7433	1282.5729	Frequencies	1215.238	1244.5951	1283.1062
Frequencies	1283.6734	1308.8223	1317.6462	Frequencies	1297.5123	1311.4364	1317.3656
Frequencies	1324.4094	1329.1264	1363.7565	Frequencies	1327.1528	1363.3986	1367.6685
Frequencies	1364.9633	1380.5652	1381.3937	Frequencies	1380.4352	1385.5204	1422.1435
Frequencies	1465.0562	1465.5805	1479.2997	Frequencies	1464.9337	1465.6722	1479.1823

Frequencies	1479.838	1501.9376	1501.9835	Frequencies	1501.8762	1502.2529	1517.4586
Frequencies	1525.3066	1525.4684	1558.5124	Frequencies	1525.2334	1535.6751	1558.5907
Frequencies	1559.0344	1576.2614	1579.4534	Frequencies	1562.1693	1573.7193	1578.3986
Frequencies	1676.0561	1677.0079	1678.7586	Frequencies	1669.7424	1676.0493	1679.435
Frequencies	1681.2789	1708.8223	1710.2034	Frequencies	1685.4166	1708.977	1814.9492
Frequencies	3083.7286	3084.8287	3176.8835	Frequencies	2583.5782	3083.2244	3085.8716
Frequencies	3177.9831	3205.5949	3206.7028	Frequencies	3176.3581	3178.7145	3205.4272
Frequencies	3222.0432	3224.7832	3242.1876	Frequencies	3207.0884	3221.6707	3233.3971
Frequencies	3242.5964	3245.5145	3250.9431	Frequencies	3241.8953	3250.4073	3272.3812
Frequencies	3272.7689	3274.765	3653.6782	Frequencies	3316.9617	3337.3489	3653.4741
Frequencies	3653.9854	3771.6846	3772.0117	Frequencies	3653.9186	3769.5731	3771.6923
PARA (174) [M+Na$^+$] (GS)				PARA (174) [M+Na$^+$] (TS)			
Frequencies	25.4575	67.4647	76.3525	Frequencies	-90.8777	22.6106	59.6704
Frequencies	98.4495	114.8444	127.3507	Frequencies	86.7098	103.7163	125.4646
Frequencies	198.7332	258.2131	356.5479	Frequencies	180.3699	257.2407	342.754
Frequencies	359.5787	388.0805	410.6684	Frequencies	355.7766	364.422	390.0461
Frequencies	432.4591	450.8334	512.6775	Frequencies	431.0411	449.7876	514.4828
Frequencies	549.1661	629.6605	637.3247	Frequencies	534.7758	631.8035	637.3198
Frequencies	640.5	667.0995	750.0668	Frequencies	646.9702	668.3957	743.4653
Frequencies	818.6341	867.6231	884.398	Frequencies	825.5667	862.065	881.4373
Frequencies	903.318	984.182	1015.4651	Frequencies	946.5257	996.9361	1016.0977
Frequencies	1025.315	1041.2152	1060.0939	Frequencies	1020.9721	1058.6594	1101.5094
Frequencies	1102.0166	1131.7334	1166.1837	Frequencies	1130.0257	1178.6722	1207.9737
Frequencies	1212.9291	1284.2713	1321.745	Frequencies	1247.6092	1302.1405	1331.2461
Frequencies	1334.0703	1352.8349	1370.1192	Frequencies	1355.7385	1397.9541	1457.877
Frequencies	1458.1954	1474.4987	1497.669	Frequencies	1463.5681	1497.2789	1515.3268
Frequencies	1516.0433	1555.0101	1577.1873	Frequencies	1539.0559	1558.5592	1576.2554
Frequencies	1663.25	1673.9381	1701.5521	Frequencies	1671.8537	1688.4129	1891.4094
Frequencies	3096.7279	3194.1697	3203.6566	Frequencies	2271.0159	3095.5635	3192.345
Frequencies	3219.7826	3238.7873	3242.5878	Frequencies	3202.7601	3244.8289	3248.8481
Frequencies	3263.4524	3652.8187	3773.853	Frequencies	3350.8709	3651.0679	3773.5846
d_3-PARA (177) [M+Na$^+$] (GS)				d_3-PARA (177) [M+Na$^+$] (TS)			
Frequencies	25.4159	61.6395	68.0435	Frequencies	-72.2402	32.9775	53.2077
Frequencies	83.5451	111.4838	122.2933	Frequencies	72.3129	105.454	120.9013
Frequencies	198.3814	242.2428	341.0446	Frequencies	184.6402	241.7834	338.7613
Frequencies	355.3378	387.0771	410.5692	Frequencies	346.1433	367.9033	399.8084
Frequencies	432.3715	444.0985	488.7424	Frequencies	427.9187	444.8871	490.7759
Frequencies	546.141	574.4897	614.2303	Frequencies	536.6951	573.0318	615.6124
Frequencies	636.1356	660.9007	747.8105	Frequencies	644.6786	662.1712	742.8901
Frequencies	804.1836	847.4123	871.8147	Frequencies	807.3076	854.5067	867.6513
Frequencies	892.7178	896.3955	948.7701	Frequencies	890.029	934.5404	949.1569
Frequencies	969.7122	1012.2061	1024.4678	Frequencies	986.7775	1014.4594	1025.5895
Frequencies	1041.6548	1079.5706	1084.5181	Frequencies	1079.2978	1084.0004	1131.5754
Frequencies	1131.5837	1139.4237	1166.2491	Frequencies	1140.0856	1177.8981	1184.997
Frequencies	1212.9228	1284.3448	1325.0311	Frequencies	1246.6402	1302.5734	1338.8195
Frequencies	1338.4354	1355.332	1370.3372	Frequencies	1361.0116	1390.6204	1447.0154
Frequencies	1474.6904	1545.4823	1576.3272	Frequencies	1539.5988	1550.8925	1569.0483
Frequencies	1663.0831	1667.9796	1701.3564	Frequencies	1666.0072	1683.9202	1853.4797
Frequencies	2227.874	2363.0087	2375.002	Frequencies	2227.2309	2361.9098	2374.5564
Frequencies	3219.7825	3238.7871	3242.5878	Frequencies	2395.0818	3243.6885	3251.2027
Frequencies	3263.4523	3652.8169	3773.853	Frequencies	3335.4466	3651.2786	3774.2183
PARA (301) [2M+H$^+$] (GS)				PARA (301) [2M+H$^+$] (TS)			
Frequencies	7.7564	17.1395	18.167	Frequencies	-12.3094	10.9922	16.6391
Frequencies	28.3946	40.8111	44.527	Frequencies	28.9231	40.5089	44.5041
Frequencies	53.6096	62.8931	80.2549	Frequencies	51.561	61.3343	80.4644
Frequencies	84.9302	92.5705	140.7414	Frequencies	85.6875	93.4029	141.0332

Frequencies	144.5025	154.4936	197.8404	Frequencies	144.19	154.2028	197.7798
Frequencies	231.4149	260.2386	320.0397	Frequencies	230.9861	248.8487	320.4336
Frequencies	350.3643	362.571	375.6576	Frequencies	350.2808	361.4869	362.9427
Frequencies	398.7012	403.6763	409.4953	Frequencies	398.1118	403.3672	409.6834
Frequencies	434.9357	445.2969	461.2924	Frequencies	434.9189	445.5351	457.0008
Frequencies	493.5525	507.3305	536.4157	Frequencies	493.5934	510.1566	536.441
Frequencies	547.8909	569.8691	584.6684	Frequencies	547.9188	570.4556	584.526
Frequencies	591.0309	617.6651	640.102	Frequencies	591.0624	616.8425	639.7515
Frequencies	653.4941	705.9225	744.2447	Frequencies	653.5447	704.9578	744.2509
Frequencies	764.0113	784.3953	796.509	Frequencies	764.1486	783.0403	800.2821
Frequencies	802.2719	806.5586	868.2584	Frequencies	806.3615	808.2865	868.2481
Frequencies	873.4935	900.4698	914.2589	Frequencies	873.5577	900.4338	917.7157
Frequencies	920.1942	949.5641	975.5585	Frequencies	922.2578	943.4347	974.609
Frequencies	996.262	1011.0869	1035.3153	Frequencies	996.3604	1011.0732	1033.3882
Frequencies	1038.2209	1040.9728	1049.2886	Frequencies	1035.6169	1040.9782	1065.2491
Frequencies	1065.4074	1068.86	1094.451	Frequencies	1068.809	1077.7588	1094.5821
Frequencies	1113.3758	1114.6923	1146.6476	Frequencies	1113.4401	1118.6627	1146.6365
Frequencies	1173.4312	1208.1776	1218.6743	Frequencies	1173.4062	1215.4974	1218.6778
Frequencies	1262.2386	1287.5382	1300.5055	Frequencies	1262.2523	1287.6335	1302.422
Frequencies	1331.9978	1339.4081	1347.8255	Frequencies	1332.2182	1341.2848	1349.0691
Frequencies	1369.128	1385.3972	1414.0547	Frequencies	1363.6683	1385.3879	1412.4061
Frequencies	1421.7153	1431.4886	1448.9451	Frequencies	1414.3531	1440.0023	1449.0634
Frequencies	1449.9187	1488.3142	1492.9632	Frequencies	1449.9215	1488.2477	1492.963
Frequencies	1493.7739	1501.5051	1505.8096	Frequencies	1493.7749	1501.4741	1505.794
Frequencies	1555.4215	1579.1618	1619.9615	Frequencies	1556.1066	1579.1874	1620.016
Frequencies	1681.9401	1691.6113	1700.4255	Frequencies	1682.009	1704.5232	1706.6986
Frequencies	1705.7881	1721.7546	1755.5387	Frequencies	1721.2782	1721.774	1784.0436
Frequencies	1813.6113	1940.3601	3093.2926	Frequencies	1813.554	1940.0668	3093.206
Frequencies	3093.7722	3180.0881	3189.5938	Frequencies	3093.7835	3137.4521	3180.0277
Frequencies	3211.4161	3213.0558	3222.1273	Frequencies	3189.5882	3211.4318	3213.001
Frequencies	3225.9396	3234.074	3236.5858	Frequencies	3222.1394	3225.8164	3236.5772
Frequencies	3237.2801	3254.5346	3259.0319	Frequencies	3243.7134	3259.0454	3271.355
Frequencies	3271.5314	3463.0535	3764.3784	Frequencies	3281.4618	3462.8436	3764.281
PARA (158) (GS)				PARA (158) (TS)			
Frequencies	48.937	65.627	83.6614	Frequencies	-83.5896	46.3157	64.7956
Frequencies	133.4198	173.9723	207.2173	Frequencies	119.2337	151.3692	178.0502
Frequencies	243.8878	258.4044	273.381	Frequencies	225.0654	248.9513	265.3488
Frequencies	327.4946	394.2204	416.3481	Frequencies	313.7793	363.365	387.8708
Frequencies	434.1247	460.3821	477.8564	Frequencies	422.8048	439.4422	469.8132
Frequencies	516.7005	560.143	699.3006	Frequencies	501.7852	559.8043	690.8422
Frequencies	769.7039	795.2715	840.1914	Frequencies	744.1056	775.5074	783.7751
Frequencies	875.1803	938.3919	940.4678	Frequencies	864.5032	875.7178	906.6468
Frequencies	942.9671	974.0147	1004.7214	Frequencies	933.144	958.7845	977.9369
Frequencies	1035.8103	1069.7666	1078.9593	Frequencies	999.0875	1028.2566	1071.2769
Frequencies	1106.6084	1119.6669	1143.7675	Frequencies	1075.7351	1100.5545	1131.5402
Frequencies	1168.9182	1183.3039	1230.6423	Frequencies	1159.2793	1185.7464	1219.4749
Frequencies	1237.4795	1264.4541	1290.6616	Frequencies	1226.1839	1233.977	1257.2744
Frequencies	1314.4794	1324.6293	1346.2756	Frequencies	1283.5926	1306.0231	1311.4614
Frequencies	1371.7378	1385.5837	1398.4412	Frequencies	1358.8925	1381.4707	1392.6404
Frequencies	1402.517	1417.5963	1432.4021	Frequencies	1400.4351	1409.6571	1422.5986
Frequencies	1441.8303	1462.2983	1468.0511	Frequencies	1432.3696	1464.7514	1480.8161
Frequencies	1496.8913	1505.0095	1512.3078	Frequencies	1483.3709	1502.9867	1515.559
Frequencies	1514.7542	1528.4892	1533.1563	Frequencies	1522.9147	1533.2479	1545.6359
Frequencies	1667.0875	1774.1403	3059.6885	Frequencies	1653.573	1770.7491	2822.2143
Frequencies	3073.7766	3074.5151	3079.9389	Frequencies	2931.7841	2982.8583	3048.0573
Frequencies	3087.7245	3101.4666	3122.7434	Frequencies	3073.4166	3075.9183	3082.0553

Frequencies	3139.3759	3166.6443	3171.6502	Frequencies	3134.032	3163.6	3170.9798
Frequencies	3174.2014	3176.4807	3180.2419	Frequencies	3175.1041	3179.6856	3361.6061
Frequencies	3382.7231	3479.6261	3789.6001	Frequencies	3531.143	3790.3489	4125.2678
d_3-PARA (161) (GS)				d_3-PARA (161) (TS)			
Frequencies	47.5753	62.6688	81.545	Frequencies	-212.9207	28.3236	41.1588
Frequencies	128.3162	173.3187	178.4183	Frequencies	97.3822	117.4008	154.1817
Frequencies	213.4086	250.0817	260.6408	Frequencies	183.734	218.2517	241.7696
Frequencies	315.7758	378.8857	415.6625	Frequencies	283.1668	319.1203	362.0628
Frequencies	424.4675	445.4764	474.3066	Frequencies	409.2393	418.4358	463.1677
Frequencies	513.9677	535.1811	693.0304	Frequencies	494.2884	526.5828	677.7768
Frequencies	765.4255	776.5131	795.2189	Frequencies	691.1697	756.7576	773.8795
Frequencies	798.8793	847.0055	932.9208	Frequencies	781.6429	804.3557	841.8207
Frequencies	940.9335	965.8305	989.7236	Frequencies	876.0737	906.6547	928.3677
Frequencies	1002.8295	1026.9957	1053.5982	Frequencies	981.8616	1005.2553	1023.1356
Frequencies	1079.2427	1086.4662	1089.6653	Frequencies	1052.3774	1066.5668	1086.6741
Frequencies	1102.6316	1110.3868	1160.1119	Frequencies	1095.398	1098.054	1132.5541
Frequencies	1167.0542	1191.7531	1225.1907	Frequencies	1147.9938	1183.908	1204.1355
Frequencies	1232.2933	1264.4262	1291.9067	Frequencies	1215.0246	1228.2079	1250.4919
Frequencies	1314.1481	1325.0292	1343.2706	Frequencies	1269.4808	1295.5762	1310.4159
Frequencies	1370.3627	1385.6398	1398.1299	Frequencies	1355.9601	1380.9166	1390.64
Frequencies	1401.8275	1414.9158	1430.7695	Frequencies	1398.1592	1406.9144	1423.6836
Frequencies	1442.757	1468.3814	1496.9486	Frequencies	1432.6973	1477.0812	1490.7869
Frequencies	1505.0202	1512.4141	1532.8565	Frequencies	1503.2589	1526.1073	1548.5496
Frequencies	1666.7702	1774.1402	2211.8559	Frequencies	1649.542	1769.9223	2211.6782
Frequencies	2348.6295	2356.4049	3059.6888	Frequencies	2347.923	2356.184	2674.5045
Frequencies	3074.5102	3079.9375	3087.7245	Frequencies	2884.015	2960.8565	3030.7043
Frequencies	3101.4684	3122.7443	3141.2477	Frequencies	3076.1259	3082.2734	3134.1449
Frequencies	3166.6454	3174.2017	3176.481	Frequencies	3162.978	3175.2553	3439.2231
Frequencies	3382.717	3479.6182	3789.5958	Frequencies	3631.7139	3790.5076	4290.4178
PRO (260) $[M+H^+]$ (GS)				PRO (260) $[M+H^+]$ (TS)			
Frequencies	14.395	26.293	28.6955	Frequencies	-23.7942	17.9654	30.9135
Frequencies	50.4595	68.7306	94.6249	Frequencies	51.9776	69.8856	94.6248
Frequencies	125.5095	146.4524	181.3914	Frequencies	126.4008	149.2727	175.7732
Frequencies	191.826	219.6072	222.5365	Frequencies	191.9326	220.4199	223.1461
Frequencies	246.9306	271.98	309.0864	Frequencies	247.3271	272.1308	308.5166
Frequencies	339.0516	393.798	409.363	Frequencies	337.9978	393.2608	409.4263
Frequencies	419.4083	452.1118	465.9064	Frequencies	419.0981	448.2261	466.5651
Frequencies	484.2869	487.4335	492.2121	Frequencies	484.0148	488.1202	494.8789
Frequencies	507.5869	544.8928	562.8104	Frequencies	508.1825	545.6523	561.0584
Frequencies	585.1905	604.4516	620.9927	Frequencies	583.0917	604.5437	621.8459
Frequencies	682.9557	716.1388	787.6498	Frequencies	681.3543	715.5852	771.4616
Frequencies	808.2537	824.6696	825.507	Frequencies	808.3337	820.751	825.5721
Frequencies	851.1706	860.8486	877.1149	Frequencies	851.2016	857.6241	875.9484
Frequencies	913.7201	933.6243	946.5423	Frequencies	913.7452	926.0426	946.5999
Frequencies	965.0419	970.786	981.2598	Frequencies	961.3605	970.7449	981.4278
Frequencies	993.0501	1012.4809	1033.9963	Frequencies	993.2383	1011.8362	1020.3797
Frequencies	1046.1417	1050.7109	1055.4029	Frequencies	1031.3417	1047.1765	1049.7696
Frequencies	1067.5307	1079.1749	1081.9968	Frequencies	1074.9736	1079.8714	1081.2869
Frequencies	1111.3481	1122.1247	1141.3933	Frequencies	1110.7299	1122.3193	1141.3412
Frequencies	1190.2162	1195.4659	1197.9044	Frequencies	1179.0493	1194.066	1196.4741
Frequencies	1207.7327	1214.1356	1219.2215	Frequencies	1200.4041	1213.8093	1219.2234
Frequencies	1254.4648	1261.2582	1271.6238	Frequencies	1245.7272	1259.2771	1273.3937
Frequencies	1303.6424	1307.2212	1311.1366	Frequencies	1304.7423	1309.2355	1333.1164
Frequencies	1337.4497	1377.4838	1383.4438	Frequencies	1338.6701	1377.6266	1383.782
Frequencies	1402.3254	1404.2394	1416.2483	Frequencies	1402.3603	1403.7928	1413.3361
Frequencies	1433.4657	1442.3006	1456.816	Frequencies	1425.0594	1434.2404	1456.8334

Frequencies	1463.7095	1465.3853	1467.6656	Frequencies	1463.6119	1465.5268	1467.8456
Frequencies	1488.3917	1491.9632	1506.5977	Frequencies	1488.6206	1496.1345	1507.0012
Frequencies	1507.1375	1514.3208	1518.2449	Frequencies	1507.3082	1516.193	1529.1867
Frequencies	1529.1126	1532.5677	1543.7981	Frequencies	1532.6066	1533.7485	1543.8778
Frequencies	1573.2687	1664.8608	1682.7145	Frequencies	1567.64	1648.832	1692.6411
Frequencies	1707.0423	1724.4822	3073.3543	Frequencies	1707.21	1759.5058	3073.5149
Frequencies	3083.4564	3091.5311	3100.4535	Frequencies	3083.7496	3091.4885	3099.2057
Frequencies	3128.919	3129.7742	3163.8197	Frequencies	3100.0174	3129.0284	3129.8483
Frequencies	3172.3068	3179.0223	3190.6634	Frequencies	3164.0013	3171.7802	3179.2653
Frequencies	3195.7219	3205.6238	3215.6594	Frequencies	3190.9738	3195.9218	3205.7079
Frequencies	3220.2554	3225.529	3229.0174	Frequencies	3215.6733	3225.7009	3256.0371
Frequencies	3242.908	3255.3165	3256.118	Frequencies	3279.321	3280.2538	3306.7525
Frequencies	3279.647	3473.5926	3715.7811	Frequencies	3315.1513	3474.084	3715.2962
PRO (116) (GS)				PRO (116) (TS)			
Frequencies	37.7157	48.1287	57.3318	Frequencies	-46.4466	41.2591	43.0955
Frequencies	63.6572	90.7376	168.1079	Frequencies	58.8333	77.5335	171.9758
Frequencies	222.0311	229.2709	262.5555	Frequencies	220.0973	225.7221	229.5305
Frequencies	345.9043	365.5799	393.033	Frequencies	263.3963	345.5959	367.2955
Frequencies	411.8357	505.5657	526.6484	Frequencies	398.6442	498.6441	525.8064
Frequencies	703.0403	726.4956	816.8487	Frequencies	697.6482	726.3145	816.027
Frequencies	942.0748	944.6232	969.9805	Frequencies	936.4346	944.1342	969.7784
Frequencies	988.0621	996.8986	1020.3825	Frequencies	987.9203	999.0298	1023.5899
Frequencies	1118.778	1127.8659	1169.1327	Frequencies	1106.9776	1122.9833	1159.7548
Frequencies	1174.7711	1213.7436	1245.4757	Frequencies	1174.4243	1213.2052	1222.8515
Frequencies	1268.8497	1345.2983	1355.9739	Frequencies	1266.8564	1334.9373	1356.2415
Frequencies	1370.5221	1402.5502	1446.2907	Frequencies	1370.1751	1402.8774	1446.222
Frequencies	1453.1035	1464.3488	1493.6896	Frequencies	1453.8071	1464.3534	1487.2694
Frequencies	1515.0097	1516.7032	1528.6844	Frequencies	1514.9306	1516.8011	1528.7161
Frequencies	1538.164	1606.0794	1750.7656	Frequencies	1537.9339	1601.8518	1747.2343
Frequencies	1763.6733	2828.7965	3076.4334	Frequencies	1766.5529	2833.9069	3076.4891
Frequencies	3080.6483	3128.6246	3174.4242	Frequencies	3080.7018	3128.9846	3174.5523
Frequencies	3177.9239	3181.8941	3183.7385	Frequencies	3177.9842	3182.1411	3191.9217
Frequencies	3192.2053	3201.4679	3261.3634	Frequencies	3193.472	3193.9945	3271.6732
Frequencies	3308.8853	3321.2585	3791.6285	Frequencies	3314.0677	3325.1758	3900.7312
PRO (157) (GS)				PRO (157) (TS)			
Frequencies	45.3737	140.0143	175.3364	Frequencies	-182.1832	78.2049	119.0161
Frequencies	198.6368	246.0567	342.6124	Frequencies	167.8817	206.3271	318.1376
Frequencies	409.6152	452.1318	477.0952	Frequencies	333.9164	416.3383	438.4092
Frequencies	489.3008	517.6975	536.7737	Frequencies	459.0771	485.3768	520.6065
Frequencies	589.5062	618.4783	679.7152	Frequencies	544.283	568.681	619.6448
Frequencies	703.487	784.9786	806.088	Frequencies	637.4939	673.3326	712.6353
Frequencies	821.8014	849.2472	871.6396	Frequencies	755.9575	780.9156	807.6646
Frequencies	936.0996	977.7179	1000.5615	Frequencies	832.6578	850.9302	889.4918
Frequencies	1039.7058	1056.988	1062.9121	Frequencies	919.7299	966.9561	978.1736
Frequencies	1075.9001	1123.7452	1137.6867	Frequencies	1021.065	1051.5852	1072.9141
Frequencies	1185.3977	1212.71	1226.4943	Frequencies	1116.2516	1145.7157	1177.6112
Frequencies	1243.3874	1254.9714	1277.989	Frequencies	1180.7919	1215.3943	1251.3867
Frequencies	1320.4882	1419.8181	1428.9786	Frequencies	1260.725	1281.467	1347.754
Frequencies	1454.4958	1483.6534	1503.7615	Frequencies	1417.8753	1457.6803	1466.5645
Frequencies	1513.4011	1567.0593	1595.595	Frequencies	1492.806	1562.1413	1616.549
Frequencies	1654.4805	1670.3786	1716.769	Frequencies	1661.1536	1833.6867	1983.6089
Frequencies	3180.0036	3231.0543	3233.8304	Frequencies	3181.6119	3230.6888	3240.9275
Frequencies	3242.7144	3247.0916	3264.7128	Frequencies	3264.8459	3335.2194	3346.4008
Frequencies	3265.4914	3272.5052	3333.9216	Frequencies	3376.351	3535.1413	3658.8396
